# Knockdown of suppressor of glucose by autophagy (SOGA1) alleviates the progression of non-alcoholic steatohepatitis (NASH) by reducing hepatocyte senescence through regulating AMPK/mTOR-mediated mitochondrial homeostasis

**DOI:** 10.1186/s12896-026-01162-w

**Published:** 2026-05-23

**Authors:** Lijing Yan, Huanhuan Sun, Tianyu Wang, Yuling Chen, Peijie Li

**Affiliations:** 1 Department of Endocrinology, the Second Affiliated Hospital of Xi’an Jiaotong University, Xi’an, 710000 China; 2 Department of Gastroenterology, the First Affiliated Hospital of Xi’an Jiaotong University, 277 West Yanta Road, Xi’an, 710061 China; 3 Department of Cardiology, Guizhou Provincial People’s Hospital, Guiyang, 550000 China

**Keywords:** Nonalcoholic steatohepatitis, Hepatocyte senescence, Mitochondrial homeostasis, SOGA1, The AMPK/mTOR pathway

## Abstract

Suppressor of glucose by autophagy (SOGA1), a lipid regulator, reprograms energy metabolism in the liver. However, whether SOGA1 is involved in the progression of nonalcoholic steatohepatitis (NASH) remains unclear. In this study, mice were fed a high-fat diet to construct a NASH mouse model with liver fibrosis, and the results showed that SOGA1 was upregulated in liver tissues of NASH mice. Hepatocytes (NCTC1469 cells) were treated with palmitic acid (PA), and the supernatant was isolated as conditioned medium (CM) to culture hepatic stellate cells (JS1 cells), simulating NASH in vitro. SOGA1 loss of function experiments showed that silencing SOGA1 decreased the levels of inflammatory factors (TNF-α and IL-6), fibrosis markers (α-SMA, COL1A1 and TGF-β1), senescence markers (p53, p21, γ-H2AX) and mitochondrial fusion markers (OPA1, MFN1, and MFN2), and elevated the levels of mitochondrial fission marker DRP1 and autophagy related protein LC3-II/I in PA-treated NCTC1469 cells. Furthermore, CM from SOGA1 silenced NCTC1469 cells reduced the protein levels of collagen I, III and α-SMA and decreased the viability in JS1 cells. Mechanistic studies showed that SOGA1 inhibits the activation of AMPK/mTOR pathway by recruiting RNF41, reducing mitophagy and mitochondrial homeostasis, thereby accelerating hepatocyte senescence. Senescent hepatocytes promote fibrosis by secreting IL-6 to activate hepatic stellate cells. Finally, lentiviral vectors of sh-SOGA1 were injected into NASH mice and found that SOGA1 knockout alleviated NASH progression in mice. In conclusion, SOGA1 knockdown may inhibit hepatocyte senescence and hepatic stellate cell activation by enhancing AMPK/mTOR-mediated mitochondrial homeostasis, thereby alleviating NASH progression.

**Graphical Abstract:**

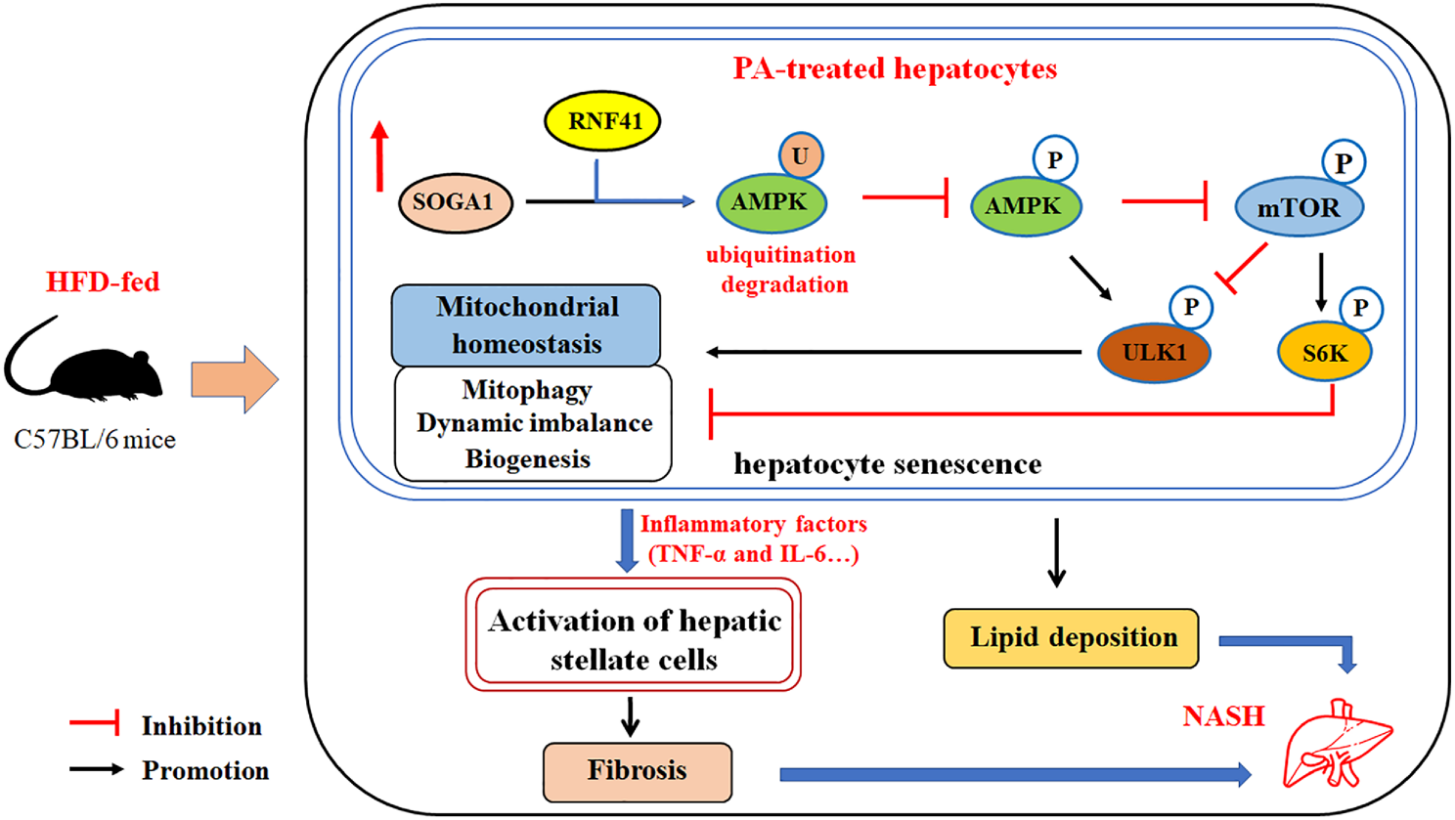

**Supplementary Information:**

The online version contains supplementary material available at 10.1186/s12896-026-01162-w.

## Introduction

Nonalcoholic fatty liver disease (NAFLD) is a chronic liver disease characterized by excessive liver fat accumulation in patients without alcohol abuse (< 20 g alcohol/day). It is characterized by liver fat accumulation, inflammation, and hepatocyte dysfunction and is closely related to obesity and metabolic syndrome [1, 2]. The late stage of NAFLD is nonalcoholic steatohepatitis (NASH), which is characterized by liver injury, inflammation and fibrosis, and further worsens to cirrhosis and end-stage liver failure, and the clinical cases of advanced cirrhosis are irreversible [3]. Currently, NAFLD is the most common chronic liver disease in developed countries. One quarter of the adult population worldwide is affected by NAFLD, and 30% of patients with NAFLD will progress to NASH [4]. Although there are many studies on NASH, its incidence continues to rise due to its complex pathogenesis and lack of effective treatment. Therefore, new and effective strategies are needed to accurately reproduce the pathogenesis of NASH to develop effective treatment strategies.

Mitochondria are highly dynamic organelles with a double-membrane structure that have important effects on basic biological functions, including energy production, metabolic regulation, calcium storage, and cell survival [5]. Mitochondrial abnormalities have been identified as early and widespread features of nonalcoholic simple fatty liver (NAFL), NASH, cirrhosis, and liver cancer, highlighting the fundamental role of mitochondria in the progression of liver disease [6, 7]. Maintaining the integrity and homeostasis of mitochondria is crucial for improving mitochondrial dysfunction, which depends on the dynamic balance of mitochondria, including the regulation of mitochondrial dynamics such as fusion and fission [5, 8]. In general, dysfunctional or damaged mitochondria are removed through a specific autophagy process called mitophagy, which is essential for maintaining mitochondrial mass and cellular homeostasis [8]. Mitophagy is involved in the pathological processes of many diseases including neurodegenerative diseases [9], atherosclerosis [10], metabolic diseases [11], and cancer [12]. However, the regulatory mechanism of mitochondrial homeostasis regulation and mitophagy in NASH remains to be further explored.

Suppressor of glucose by autophagy (SOGA1) is a relatively atypical protein that participates in the adiponectin-mediated inhibition of glucose production by inhibiting autophagy in hepatocytes in an insulin-dependent manner [13]. Moreover, SOGA1 plays a crucial role in spindle assembly checkpoint and corrects chromosome segregation [14]. Kruse et al. found that SOGA1 is a microtubule-associated protein that binds to glycogen synthase and glycogenin to directly and synergistically regulate glucose and glycogen metabolism [15]. SOGA1 is upregulated in liver cancer, which can be used as a diagnostic and prognostic biomarker for liver cancer patients [16]. In addition, a previous report showed that the mRNA level of SOGA1 was elevated in the liver tissues of obese male mice induced by bisphenol S [17]. However, whether SOGA1 is involved in NASH progression, and its mechanism of action are still unclear.

In this study, we constructed a NASH mouse model with liver fibrosis by feeding C57BL/6 mice a high-fat diet, and evaluated the expression of SOGA1 in hepatocytes were treated with palmitic acid (PA) and their supernatants were isolated as a conditioned medium (CM) to culture hepatic stellate cells to simulate NASH in vitro, which was used to explore the regulatory mechanism of SOGA1 in NASH. Our study further improves the regulatory network of NASH pathogenesis and attempts to provide a new perspective for the development of effective therapeutic strategies for NASH.

## Materials and methods

### Clinical samples

Serum samples were obtained from healthy volunteers (*N* = 48), patients with non-alcoholic simple fatty liver (NAFL, *N* = 45) and patients with non-alcoholic steatohepatitis (NASH, *N* = 51). The exclusion criteria were described as following: individuals with a history of excessive alcohol consumption (> 140 g for men or > 70 g for women, per week), drug or toxin use, viral infection or other liver diseases were excluded from the study. NAFLD activity score (NAS) was following the roles: cases with a NAS ≥ 3 or 5 were classified as NAFL and NASH respectively. Samples with NAS of 0 were treated as normal controls. The scores for steatosis (0–3), and fibrosis was staged on a 0–4 scale [18]. This study was performed with approval from the Ethics Committee at the Second Affiliated Hospital of Xi’an JiaoTong University (XJTU-2024027) and adhered to the principles of the Declaration of Helsinki. Informed written consent was obtained from all subjects.

### Establishment of a NASH mouse model with liver fibrosis

All experimental procedures were approved by the Second Affiliated Hospital of Xi’an Jiaotong University Animal Care and Use Committee (XJTU-2024027) and all animals received humane care in accordance with the guidelines of the National Institutes of Health. One hundred and twelve C57BL/6 mice aged 4 weeks (18–22 g) were purchased from the Experimental Animal Center of Xi’an Jiaotong University. Twelve mice were fed a high-fat diet (HFD) (carbohydrates accounted for 32.6% of total calories, fat 51.0%, and protein 16.4%. D12492, Research Diets) for 8 weeks to construct a NASH mouse model with liver fibrosis, whereas 12 mice in the Normal chow group were fed a normal chow (carbohydrates accounted for 62.3% of total calories, fat 12.5%, and protein 24.3%. D12450J, Research Diets) as control. In addition, 40 mice were randomly divided into four groups: normal chow, HFD, HFD + NC shRNA, and HFD + sh-SOGA1, with 10 mice in each group. The mice in the Normal chow and HFD groups were treated as described above. Mice in the HFD + NC shRNA, and HFD + sh-SOGA1 groups were fed with HFD for 8 weeks and were injected with lentiviral vectors of NC shRNA and sh-SOGA1, respectively (1 × 10^9^ TU/mL).

Model 2: Forty-eight mice were randomly divided into six groups: Normal chow, HFD, HFD + NC shRNA, HFD + sh-SOGA1, HFD + sh-SOGA1 + CQ, and HFD + sh-SOGA1 + CC, with 8 mice in each group. The mice in the Normal chow, HFD, HFD + NC shRNA, HFD + sh-SOGA1 groups were treated as described above. Mice in the HFD + sh-SOGA1 + CQ group were fed with HFD for 8 weeks and were daily injected (IP) with chloroquine (CQ, 20 mg/kg) for 1 week; and mice in the HFD + sh-SOGA1 + CQ group were fed with HFD for 8 weeks and were injected (IP) with Compound C (CC, 25 mg/kg).

At the end of the experiment, all mice were anesthetized with 150 mg/kg sodium pentobarbital by intraperitoneal injection. Blood samples and liver tissues were stored at -80 °C for subsequent experimental analysis.

### Cell culture

Mouse normal liver cell line (NCTC1469 cells) and hepatic stellate cell line (JS1 cells) were obtained from Cell Bioscience and cultured in DMEM (Thermo Fisher Scientific) with 10% fetal bovine serum (FBS, Thermo Fisher Scientific) and 1% penicillin and streptomycin (PS) in a humidified incubator with 5% CO_2_ at 37 °C. The cells were treated with 200 µM of palmitic acid (PA) for 3 h to simulate NASH in vitro. Meanwhile, the cells were pretreated with 10 µM CQ for 2 h to inhibitor autophagy, and were pretreated with 25 µM CC for 2 h to block the activation of AMP-activated protein kinase (AMPK) pathway.

### Cell transfection

SOGA1 shRNA (sh-RPS3A) and overexpression vectors (pcDNA-SOGA1), RNF41 shRNA (sh-RNF41) and overexpression vectors (pcDNA-RNS41), and control (NC shRNA and pcDNA-vector) were synthesized by GenScript Co., Ltd. (Beijing, China). At 70% confluence, the cells were transfected with the above vectors for 48 h using Lipofectamine^®^3000 (Thermo Fisher Scientific, Inc., USA), according to the manufacturer’s instructions. And then cells were harvested, and the overexpression and silencing efficiencies were evaluated using RT-qPCR. The following shRNA sequence of SOGA1 was used: 5’-GAGCTTTCACATTAAGGTAAA-3’.

### Haematoxylin and eosin staining

Liver tissues were fixed in 10% formalin solution, embedded routinely in paraffin, and cut into 5 μm-thick sections. After deparaffinization and hydration, the sections were deparaffinized with xylene, stained with hematoxylin and eosin (H&E), and observed under a light microscope (BX53, Olympus, Japan).

### Oil Red O staining

Liver tissues were fixed in 4% paraformaldehyde, embedded in paraffin, and cross-sectioned to obtain 5 μm thickness, which were observed by Oil Red O staining, according to the manufacturer’s protocol.

### Senescence-associated β-galactosidase staining

SA-β-gal staining kit was obtained from Beyotime Biotechnology (Shanghai, China). NCTC1469 cells were grown in 24-well plates and 0.6 mL of fixed buffer was added to each well for 15 min at room temperature. The staining mixture was added to sections of NCTC1469 cells and liver tissues, and then incubated overnight at 37 °C, and observed under a microscope (Nikon, Tokyo, Japan).

### Immunofluorescence staining

The sections were dewaxed, hydrated, antigen repaired, and blocked with 10% FBS for 30 min at room temperature. Sections were then incubated with BODIPY, MitoTracker, or LC3B antibodies overnight at 4 °C. Next, the sections were washed with PBS and incubated with secondary antibodies, donkey anti-mouse IgG (H + L) highly cross-absorbed Alexa Fluor Plus 647 (A32795, 1:500) for 45 min at room temperature. The sections were washed and the nuclei were stained with DAPI (Thermo Fisher Scientific) for 5 min at room temperature. Images were acquired using a Zeiss AxioImager Z1 epifluorescence microscope (Nikon, Tokyo, Japan) and analyzed using Zeiss ZEN Blue microscopy software.

### Masson’s trichrome staining

The procedure was performed according to the manufacturer’s protocol (Muto Pure Chemical Co., Ltd., Tokyo, Japan). The sections were deparaffinised and treated with the first mordant solution for 20 min. Next, the nuclei were stained with Weigert’s iron hematoxylin solution for 10 min. The sections were then washed under running tap water for a few minutes, treated with the second mordant for 30 s, 0.75% orange G solution for 1 min, and washed with 1% acetic acid solution. The nuclei were then immersed in Masson’s dye B for 20 min, washed with a 1% acetic acid solution, and immersed in a 2.5% phosphotungstic acid solution for 20 min. After washing again with 1% acetic acid solution, the sections were immersed in aniline blue dye for 10 min to stain the collagen fibers. Images were obtained using an optical microscope (Nikon, Tokyo, Japan). The blue-colored area was quantified using the ImageJ software.

### Transmission electron microscopy (TEM)

The cells were digested and fixed with 4% glutaraldehyde for 2 h. Next, cells were fixed with 1% osmium tetroxide at 4℃. After graded alcohol and acetone dehydration, cells were embedded in Epon 816 (Electron Microscopy Sciences, Hatfield, PA, USA). Sections were made using a Leica ultramicrotome, stained with uranyl acetate and citric acid. Finally mitochondrial morphology and autophagosomes in NCTC1469 cells were observed by JEM-1400Plus transmission electron microscope (Tokyo, Japan).

### Serum biochemical analysis

The plasma of the mice was collected and centrifuged to obtain serum. The levels of ALT, AST, TC, and TG were detected using assay kits according to the manufacturer’s protocol.

### Cell viability assay

JS1 cells growing in 96-well microplates after incubation for 48 h, 10 µL of Cell Counting Kit-8 (CCK-8) reagent (Sigma-Aldrich, St. Louis, MO, USA) was added to each well and incubated at 37 °C for another 2 h in a humidified atmosphere with 5% CO_2_. Absorbance was measured at 450 nm using a microplate reader (Molecular Devices).

### Quantitative reverse transcription PCR (RT-qPCR)

The total RNA of liver tissues, NCTC1469 and JC cells was extracted using TRIzol reagent (Invitrogen, Carlsbad, CA, USA). RNA (2 µg) was reverse-transcribed using the RevertAid First Strand cDNA Synthesis Kit (Thermo Scientific, USA). PCR amplification was performed using the fluorescent dye SYBR™ Green PCR Master Mix (Thermo Fisher, USA) according to the manufacturer’s protocol. Gene expression was determined using the 2^−ΔΔCt^ method by normalizing to the amount of the internal control (β-actin).

### Western blotting

Liver tissues and NCTC1469 and JC cells were homogenized in RIPA buffer (Beyotime, China), and protein concentration was measured using a BCA kit (Beyotime, China). Protein samples were separated on 12% SDS-polyacrylamide gradient gels (PAGE) and transferred onto polyvinylidene difluoride (PVDF) membranes. The membranes were blocked by the incubation with 5% non-fat dry milk for 1 h at room temperature, and then incubated with primary antibodies, including primary antibodies: anti-SOGA1, anti-LC3, anti-p62, anti-α-SMA, anti-COL1A1, anti-TGF-β1, anti-p53, anti-p21, anti-γ-H2AX, anti-Collagen Ⅰ, anti-Collagen Ⅲ, anti-LC3Ⅱ/Ⅰ, anti-p62, anti-PINK1, anti-COX Ⅳ, anti-TFAM, anti-NRF1, anti-NRF2, anti-OPA1, anti-MFN1, anti-MFN2, anti-DRP1, anti-AMPK, anti-p-AMPK, anti-mTOR, anti-p-mTOR, anti-COX Ⅳ, anti-RNF41 and anti-GAPDH antibodies (Dilution ratio: 1:500; All antibodies were purchased from Abcam), overnight at 4 °C. The following day, the membranes were incubated with HRP anti-rabbit IgG secondary antibody (1:500, Abcam) for 2 h at room temperature. Finally, the membranes were visualized using ECL reagent, and Fuji Image-Gauge software (Fujifilm North America Corporation, Valhalla, NY, USA) was used to measure band intensity.

### Co-immunoprecipitation (Co-IP) analysis

The cells were rinsed with PBS and lysed in lysis buffer. Cell lysates were cleared by centrifugation at 500 g for 10 min, and the supernatants were incubated overnight with anti-SOGA1 antibodies conjugated glutathione-agarose beads. After incubation, the proteins bound to the beads were dissociated with buffer solution and then detected by Western blotting.

### Measurement of the mitochondrial membrane potential (ΔΨm)

The changes in the mitochondrial membrane potential (MMP) were measured by MMP assay kit with JC-1 (Beyotime). The cells were harvested and resuspended in PBS and then incubated with JC‐1 for 20 min in 37 °C. After measuring by flow cytometry, the fluorescence intensity was analyzed.

### Measurement of ATP production

The cellular ATP levels were measured using an ATP bioluminescent assay kit (Beyotime, China). The assay was carried out in accordance with the manufacturer’s instruction.

### Measurement of reactive oxygen species (ROS) production

The cells were collected and the production of ROS was evaluated by ROS-sensitive fluorescent probe 5-(and-6)-chloromethyl-2’,7’-dichlorodihydrofluorescein, acetyl ester (CM-H2DCFDA) (Invitrogen, Life Technologies, Ltd.). Briefly, cells (2 × 10^5^ cell/well) were incubated with 2’,7’-Dichlorodihydrofluorescein diacetate (DCFH-DA) fluorescent probe (10 µM/well) for 20 min at room temperature in the dark, and ROS production was detected by measuring the fluorescence intensity recorded at 495 nm excitation and 527 nm emission, using a microplate reader (Varian Cary Eclipse Fluorescence Spectrophotometer).

### Statistical analysis

Quantitative data are presented as mean ± SD, and comparisons between two groups were performed using Student’s *t*-test. Statistical comparisons between multiple groups were performed using one-way or two-way analysis of variance (ANOVA) followed by the least significant difference (LSD) test. *P*-value < 0.05.

## Results

### SOGA1 was upregulated in liver tissues of HFD-induced NASH mice with fibrosis

**Fig. 1:**
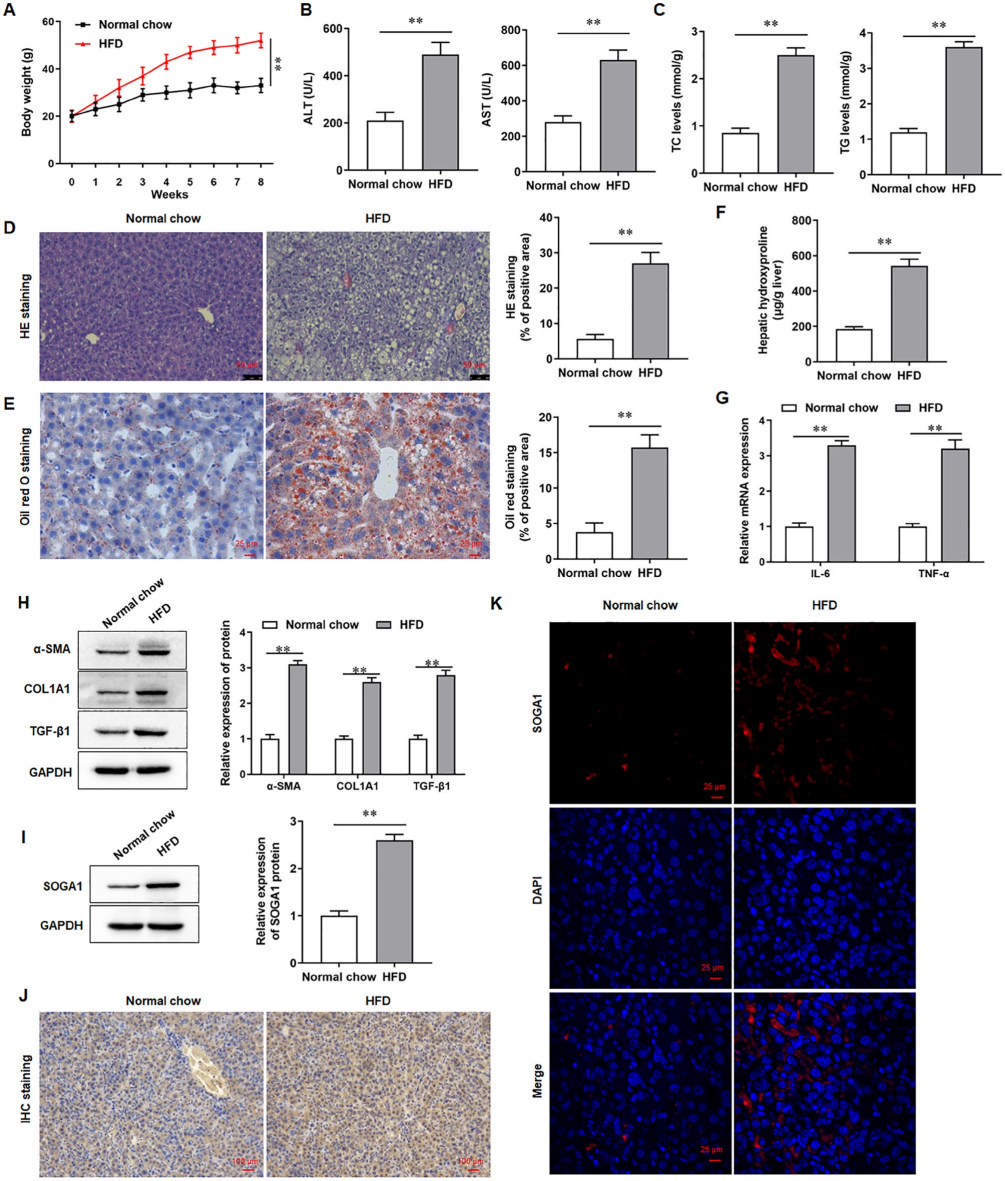
SOGA1 was upregulated in liver tissues of HFD-induced NASH mice with fibrosis. Twenty-four mice were randomly divided into the normal chow group and HFD group, with 12 mice in each group. Mice in HFD group were fed with high-fat diet for 8 weeks, while mice in the normal chow group were fed with standard diet. After 8 weeks, all mice were euthanized and weighed (**A**), and blood and liver tissues were collected. **B**. The activities of AST and ALT in serum were detected with biochemical kits; **C**. The contents of TC and TG in serum were detected with biochemical kits; **D**. Liver histopathological changes were observed by HE staining; **E**. Lipid deposition in liver tissues was analyzed by oil red O staining; **F**. Hydroxyproline assay kit was used to detect the hydroxyproline level in liver tissues of mice; **G**. The mRNA levels of IL-6 and TNF-α in liver tissues of mice were detected with RT-qPCR; **H.** The protein levels of fibrosis markers α-SMA, COL1A1 and TGF-β1 in liver tissues of mice were detected by Western blotting. The protein levels of SOGA1 in liver tissues of mice were detected by Western blotting (**I**), Immunohistochemical staining (**J**) and Immunofluorescence staining (**K**). Data shown are the mean ± SD, sample size (N) = 5. The statistical differences were evaluated by Student’s *t*-test. Compared to normal chow group, ** *P* < 0.01

To explore whether SOGA1 is involved in NASH occurrence and development, we fed C57BL/6 mice a high-fat diet to construct a NASH mouse model with fibrosis. The results showed that the body weight of the mice in the HFD group was significantly higher than that in the normal chow group (Fig. 1A). Compared with the normal chow group, the serum levels of ALT, AST, TC, and TG were higher in mice of the HFD group (Fig. 1B and C). HE staining results showed that the structure of the liver tissue of mice in the normal chow group was clear and complete, and the liver tissue of mice in the HFD group showed obvious histopathological changes, including liver tissue structure damage, local tissue necrosis, and a large amount of inflammatory cell infiltration (Fig. 1D). Oil Red O staining showed that lipid deposition was increased in the liver tissue of HFD group mice compared with that in the normal chow group (Fig. 1E). Moreover, we found that the level of hydroxyproline and inflammatory factors IL-6 and TNF-α in the liver tissue of mice in the HFD group was significantly higher than that in mice of the normal chow group (Fig. 1F and G). Western blotting results showed that compared with the normal chow group, the protein levels of the fibrosis markers α-SMA, COL1A1, and TGF-β1 were increased in the liver tissue of mice in the HFD group (Fig. 1H). Based on the above results, we concluded that the NASH mouse model with fibrosis was successfully constructed.

In addition, we detected the expression level of SOGA1 in the liver tissue of mice by using Western blotting and found that the protein level of SOGA1 in the liver tissue of mice in the HFD group was significantly higher than that in mice of the normal chow group (Fig. 1I). Similar phenomena were observed in IHC staining and immunofluorescence analysis, and SOGA1 was significantly upregulated in the liver tissue of mice in the HFD group (Fig. 1J and K). Based on this, we speculated that SOGA1 may be involved in the progression of NASH.

### Knockdown of SOGA1 attenuated PA-induced lipid deposition and fibrosis in NCTC1469 cells

**Fig. 2:**
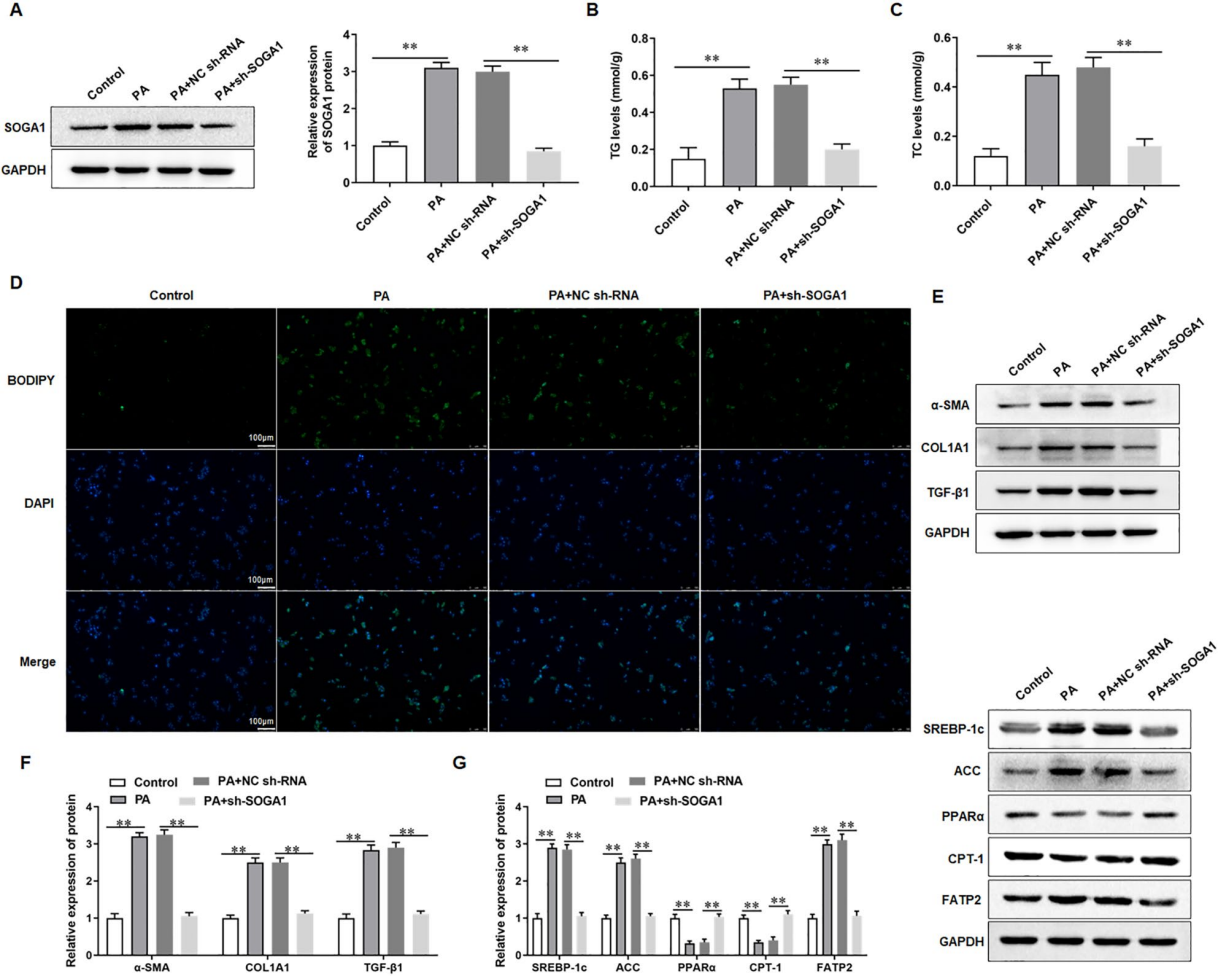
Knockdown of SOGA1 alleviated PA-induced lipid deposition and fibrosis in NCTC1469 cells. NCTC1469 cells pretreated with 200 µM PA were transfected with NC shRNA or sh-SOGA1 for 48 h. **A**. The protein level of SOGA1 was detected with Western blotting; **B** and **C**. The contents of TC and TG were detected with biochemical analysis kits; **D**. Lipid content was detected with BODIPY fluorescence staining; **E** and** F**. The protein expression levels of fibrosis markers α-SMA, COL1A1 and TGF-β1 were detected with Western blotting. **G.** The protein levels of fatty acid synthesis markers SREBP1c and ACC, fatty acid uptake marker FATP2, and fatty acid oxidation markers PPARα and CPT-1 were detected with Western blotting. *N* = 5, ** *P* < 0.01. Data shown are the mean ± SD, sample size (N) = 5. The statistical differences were evaluated by one-way ANOVA, and followed by LSD test. Compared to control group or PA + NC sh-RNA group, ** *P* < 0.01

Mouse hepatocytes (NCTC1469 cells) were treated with palmitic acid (PA) to simulate NASH in vitro. Western blotting results showed that PA treatment increased the protein level of SOGA1 in NCTC1469 cells, while silencing SOGA1 decreased SOGA1 protein levels (Fig. 2A). Furthermore, PA treatment increased the levels of TC and TG in NCTC1469 cells, and silencing of SOGA1 decreased TC and TG levels (Fig. 2B and C). BODIPY 493/503 is a lipophilic fluorescent probe that can be used to label the lipid content in cells. PA significantly enhanced BODIPY fluorescence intensity in NCTC1469 cells, whereas silencing SOGA1 decreased BODIPY fluorescence intensity (Fig. 2D). Meanwhile, PA treatment increased the protein levels of fatty acid synthesis markers SREBP-1c and ACC, as well as the protein level of fatty acid uptake marker FATP2, and decreased the protein levels of fatty acid oxidation markers PPARα and CPT-1, while silencing SOGA1 decreased SREBP-1c, ACC and FATP2 protein levels and increased PPARα and CPT-1 protein levels (Fig. 2G). In addition, PA treatment increased the protein levels of fibrosis markers α-SMA and COL1A1 and the potent pro-fibrotic cytokine TGF-β1 in NCTC1469 cells, while silencing SOGA1 reduced the protein levels of α-SMA, COL1A1, and TGF-β1 (Fig. 2E and F). These results suggested that SOGA1 may participate in NASH progression by promoting lipid deposition and fibrosis in hepatocytes.

### Knockdown of SOGA1 attenuated PA-induced hepatocyte senescence

**Fig. 3:**
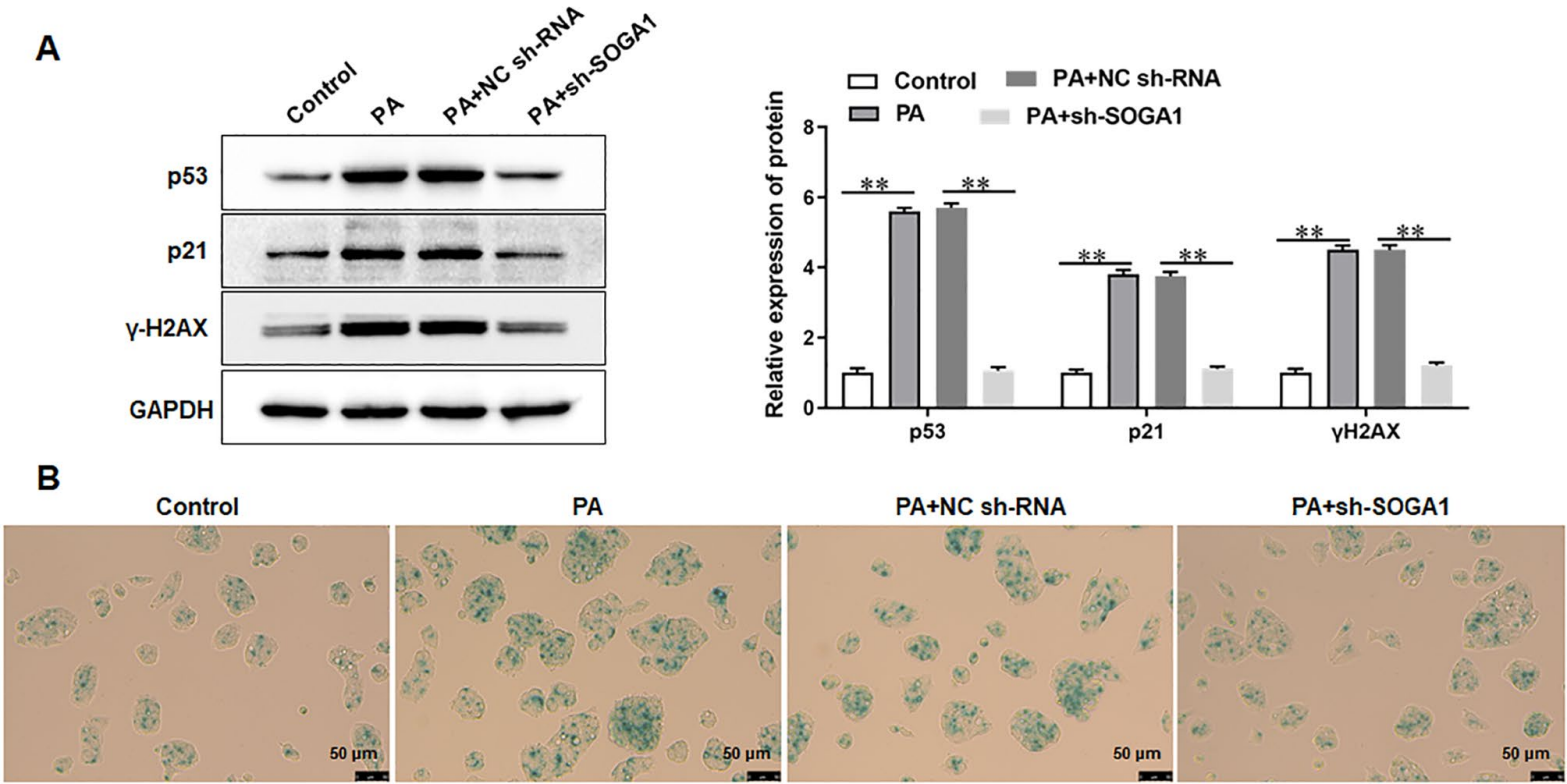
Knockdown of SOGA1 alleviated PA-induced hepatocyte senescence. NCTC1469 cells pretreated with 200 µM PA were transfected with NC shRNA or sh-SOGA1 for 48 h. **A**. The expression levels of senescence marker proteins p53, p21 and γ-H2AX were detected with Western blotting; **B**. Senescent hepatocytes were evaluated by β-galactosidase staining. Data shown are the mean ± SD, sample size (N) = 5. The statistical differences were evaluated by one-way ANOVA, and followed by LSD test. Compared to control group or PA + NC sh-RNA group, ** *P* < 0.01

The report showed that hepatocyte senescence promotes lipid deposition and steatosis in hepatocytes, which is one of the key mechanisms for NASH to develop into liver cancer [19, 20]. Western blotting results showed that PA treatment increased the levels of the senescence-related proteins p53, p21, and γ-H2AX in NCTC1469 cells, and silencing SOGA1 reduced the protein levels of p53, p21, and γ-H2AX (Fig. 3A). Interestingly, β-galactosidase staining showed that silencing SOGA1 reduced the intensity of senescence-associated β-galactosidase positive staining in PA-treated NCTC1469 cells (Fig. 3B). Taken together, these results indicated that SOGA1 knockdown attenuates PA-induced hepatocyte senescence in vitro.

### Knockdown of SOGA1 reduced hepatic stellate cell activation by inhibiting IL-6 secretion from senescent hepatocytes

**Fig. 4:**
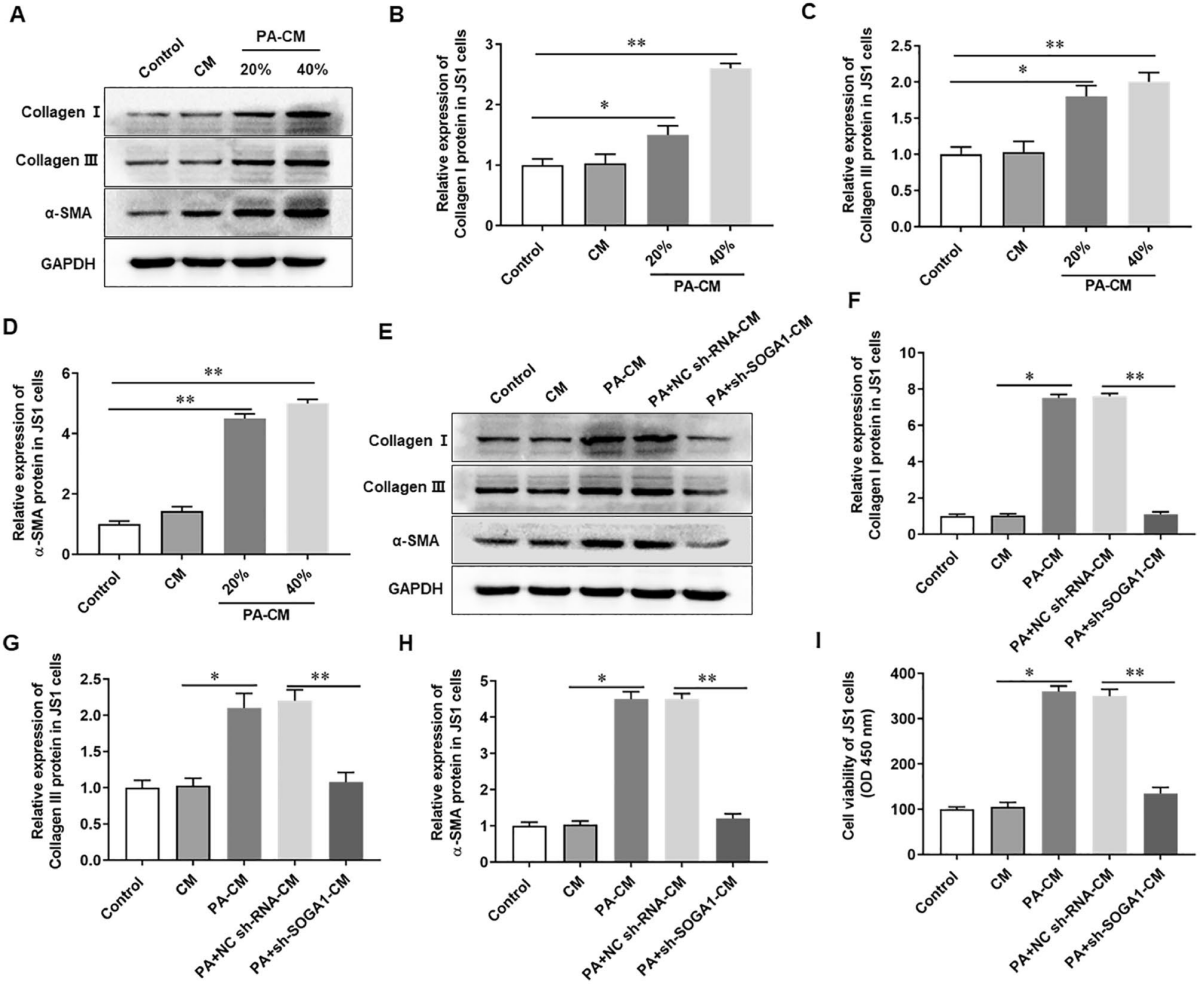
Knockdown of SOGA1 reduced the activation of hepatic stellate cells by inhibiting hepatocyte senescence. **A-D**. The supernatants of PA treated or untreated NCTC1469 cells were isolated as conditioned medium (CM) to culture JS1 cells for 48 h. The protein expression levels of collagen Ⅰ, collagen Ⅲ and α-SMA were detected with Western blotting. NCTC1469 cells pretreated with PA were transfected with NC shRNA and sh-SOGA1, and then the supernatant was isolated as CM to culture JS1 cells for 48 h. **E-H**. Western blotting was used to detect the protein levels of collagen Ⅰ, collagen Ⅲ and α-SMA; **I**. The viability of JS1 cells was detected with CCK-8. Data shown are the mean ± SD, sample size (N) = 5. CM: conditioned medium. The statistical differences were evaluated by one-way ANOVA, and followed by LSD test. Compared to control group, CM group, or PA + NC sh-RNA-CM group, * *P* < 0.05, ** *P* < 0.01

Hepatic stellate cells (HSCs) are the main effector cells involved in liver fibrosis. Existing reports show that factors secreted by senescent hepatocytes participate in liver fibrosis by activating hepatic stellate cells [21, 22]. To explore the effect of SOGA1 regulated hepatocyte senescence on hepatic stellate cell activation, we isolated the supernatant of PA-induced NCTC1469 cells as conditioned medium (CM) to culture hepatic stellate cells (JS1 cells). The results showed that compared with the control, untreated NCTC1469 cell-derived CM had no effect on the protein levels of extracellular matrix components collagen I and collagen III, while the CM of NCTC1469 cells induced by PA promoted the protein expression of collagen I and collagen III in a dose-dependent manner (Fig. 4A-C). In addition, compared with the control, the protein level of α-SMA, an activation marker of hepatic stellate cells, was increased in JS1 cells treated with CM from normal hepatocytes, but the difference was not statistically significant, whereas CM derived from NCTC1469 cells induced by PA promoted the protein expression of α-SMA in JS1 cells (Fig. 4A and D). The above results indicated that CM derived from senescent hepatocytes promotes the activation of hepatic stellate cells. At the same time, we also found that CM from NCTC1469 cells with SOGA1 knockdown significantly decreased the protein expression of collagen I, collagen III, and α-SMA in JS1 cells and counteracted the activation of PA-induced NCTC1469 cell-derived CM in JS1 cells (Fig. 4E-I).

In addition, we also identified the expression of specific senescence associated secretory phenotype (SASP) factors TNF-α, IL-1β, IL-6, PDGF, and TGF-β in senescent hepatocytes with SOGA1 knockdown, and found that the protein levels of TNF-α, IL-1β, IL-6, PDGF, and TGF-β were significantly decreased, especially IL-6 (Supplementary Fig. 1A). Therefore, we speculated that SOGA1 may promote hepatic stellate cell activation by regulating the secretion of IL-6 in senescent hepatocytes. To verify our hypothesis, hepatic stellate cells were treated with CM from senescent hepatocytes with SOGA1 knockdown, while simultaneously adding mouse IL-6 recombinant protein (rmIL-6). The results showed that rmIL-6 significantly increased the protein levels of collagen Ⅰ, Ⅲ, and α-SMA and enhanced the viability in JS1 cells. rmIL-6 significantly reversed the inhibitory effect of CM from senescent hepatocytes with SOGA1 knockdown on the activation of hepatic stellate cells (Supplementary Figs. 1B and C). The above results suggested that knockdown of SOGA1 may attenuate hepatic stellate cell activation by inhibiting the secretion of IL-6 by senescent hepatocytes, thereby alleviating the progression of liver fibrosis.

### Knockdown of SOGA1 alleviated hepatocyte senescence by enhancing mitophagy

**Fig. 5:**
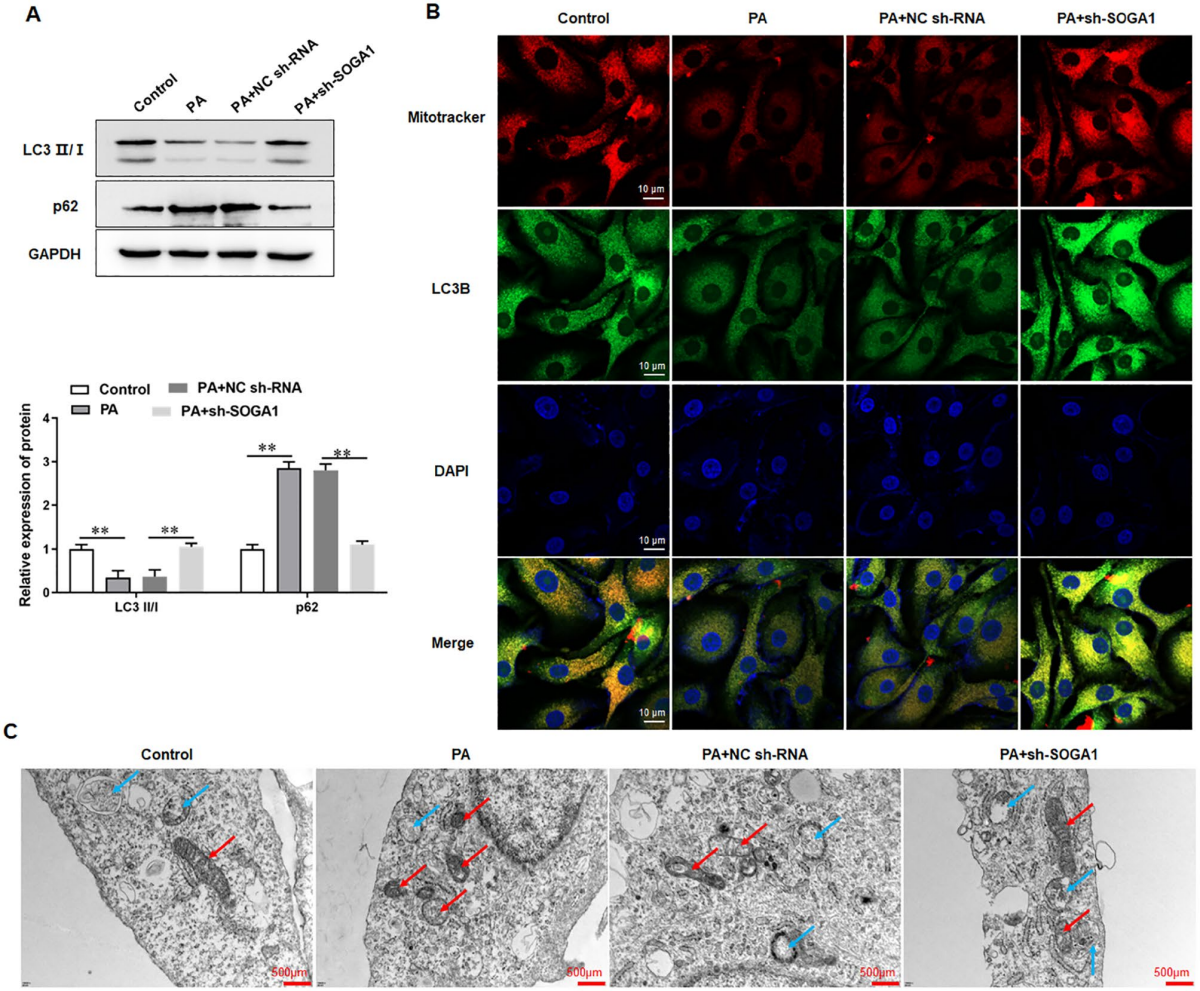
Knockdown of SOGA1 alleviated hepatocyte senescence by enhancing mitophagy. NCTC1469 cells pretreated with 200 µM PA were transfected with NC shRNA or sh-SOGA1 for 48 h. **A**. The protein levels of autophagy related proteins LC3-II/I and p62 were detected with Western blotting; **B**. The aggregation of autophagy protein LC3B on mitochondria was analyzed by dual fluorescence co-localization; **C.** Cell ultrastructure was observed by Transmission electron microscopy, red arrows indicate mitochondria and blue arrows indicate autophagosomes. Data shown are the mean ± SD, sample size (N) = 5. The statistical differences were evaluated by one-way ANOVA, and followed by LSD test. Compared to control group or PA + NC sh-RNA group, ** *P* < 0.01

Impaired autophagy is a characteristic feature of cellular senescence. To explore whether SOGA1 regulates hepatocyte senescence through the autophagy pathway, we examined the expression of autophagy-related proteins in PA-treated NCTC1469 cells. The results showed that PA treatment decreased the level of autophagy-related protein LC3-II/I and increased the protein level of p62, whereas silencing SOGA1 increased the protein level of LC3-II/I and decreased the protein level of p62 (Fig. 5A). Mitophagy, a selective autophagy process, is a key quality-control mechanism for maintaining liver homeostasis [23]. Therefore, we explored the effects of SOGA1 silencing on mitophagy. The double fluorescence co-localization results of Mitotracker (red fluorescence) and autophagy protein LC3B (green fluorescence) showed that PA treatment significantly reduced the fluorescence intensity of LC3B in mitochondria, while silencing SOGA1 enhanced the fluorescence intensity of LC3B in mitochondria and promoted the expression of LC3B in mitochondria (yellow fluorescence) (Fig. 5B). Meanwhile, we observed the ultrastructure of hepatocytes by using the transmission electron microscope, and found that many long-shaped mitochondria were observed in hepatocytes of the control group, while mitochondria swelling with disappearance of cristae were observed in hepatocytes of the PA group and the PA + NC shRNA group. Furthermore, massive mitophagy was observed in the hepatocytes with SOGA1 knockdown (Fig. 5C).

To further determine whether SOGA1 is involved in autophagy regulation in NASH, we treated SOGA1 shRNA transfected NCTC1469 cells with chloroquine (CQ), an autophagy inhibitor. The results showed that CQ treatment significantly reduced the LC3-II/I ratio and increased p62 protein levels in NCTC1469 cells treated with PA (Supplementary Fig. 2A). Additionally, CQ treatment reversed the autophagy activation induced by SOGA1 silencing and counteracted the effects of SOGA1 silencing on NCTC1469 cell senescence and fatty acid metabolism, as well as JS1 cell activation and fibrosis (Supplementary Figs. 2A-F). These results suggested that SOGA1 knockdown may alleviate hepatocyte senescence by inducing mitophagy.

### Knockdown of SOGA1 inhibited PA-induced hepatocyte senescence by enhancing mitochondrial homeostasis

**Fig. 6:**
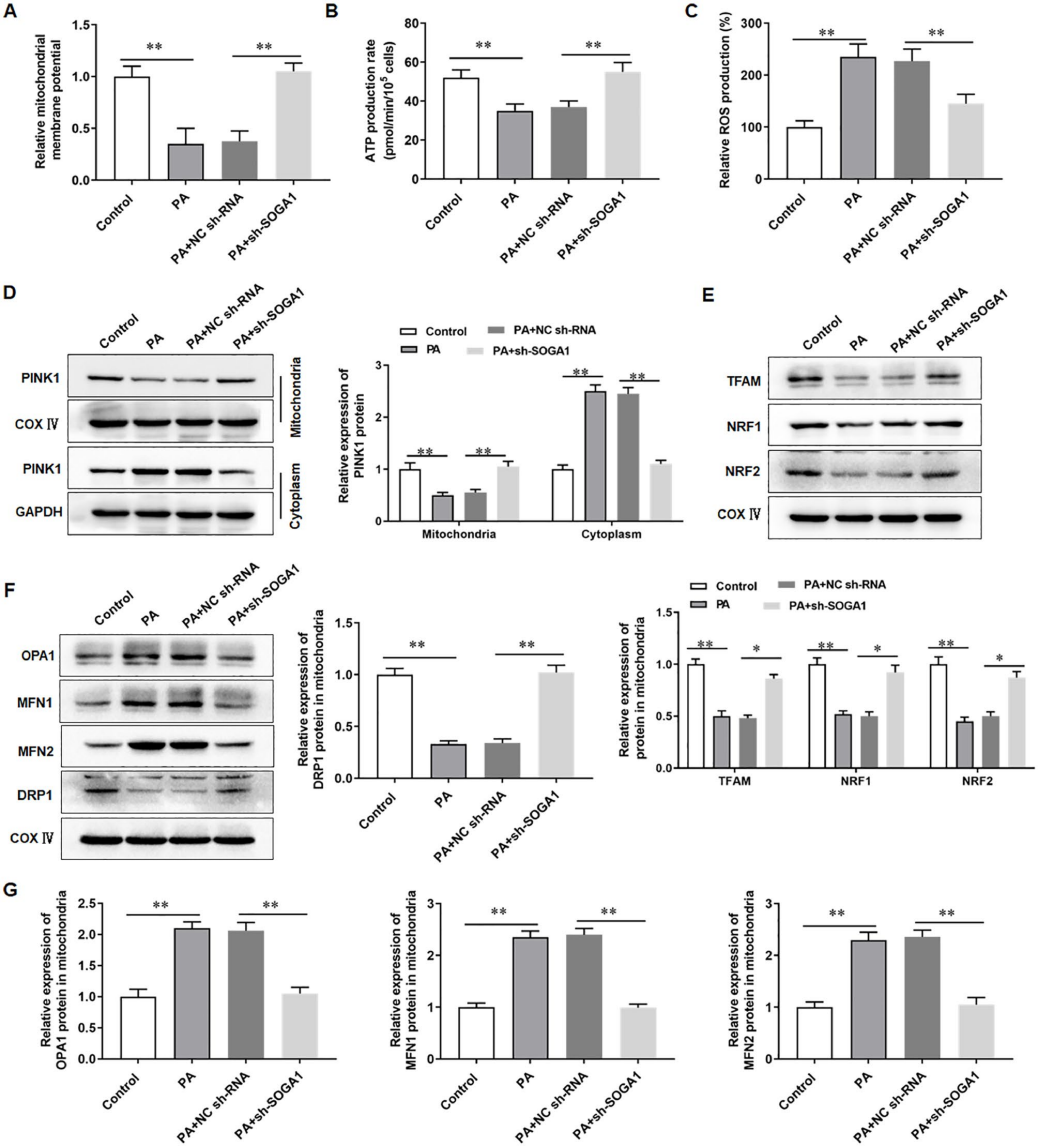
Knockdown of SOGA1 inhibited PA-induced hepatocyte senescence by enhancing mitochondrial homeostasis. NCTC1469 cells pretreated with 200 µM PA were transfected with NC shRNA or sh-SOGA1 for 48 h. **A.** The mitochondrial membrane potential (MMP) was measured by flow cytometry; **B.** ATP levels were measured by ATP bioluminescent assay kits; **C.** ROS production levels were measured by flow cytometry; **D**. The protein expression level of PINK1 in the cytoplasm and mitochondria was detected with Western blotting; **E**. The expression levels of mitochondrial biogenesis related proteins TFAM, NRF1 and NRF2 in mitochondria were detected with Western blotting; **F **and** G**. The expression levels of fusion proteins OPA1, MFN1, MFN2 and fission protein DRP1 in the of NCTC1469 cells were detected with Western blotting. Data shown are the mean ± SD, sample size (N) = 5. The statistical differences were evaluated by one-way ANOVA, and followed by LSD test. Compared to control group or PA + NC sh-RNA group, * *P* < 0.05, ** *P* < 0.01

Existing reports shown that mitochondria are interconnected and regulated by mitophagy, mitochondrial biogenesis and mitochondrial dynamic balance to jointly maintain mitochondrial function, homeostasis, quality control and cell survival [24, 25]. The results showed that PA treatment significantly reduced the mitochondrial membrane potential and ATP production levels and increased ROS production levels in NCTC1469 cells, whereas SOGA1 silencing increased the mitochondrial membrane potential and ATP production levels and decreased ROS production levels (Fig. 6A-C). Next, we explored the effect of SOGA1 silencing on PA-induced mitochondrial biogenesis and mitochondrial dynamic balance in hepatocytes. The results showed that PA treatment decreased the protein level of PINK1 in the mitochondria and increased the protein level of PINK1 in the cytoplasm, while silencing SOGA1 promoted the aggregation of PINK1 protein in the mitochondria (Fig. 6D). Moreover, PA treatment decreased the levels of the mitochondrial biogenesis-related proteins TFAM, NRF1, and NRF2, while silencing SOGA1 partially increased the protein levels of TFAM, NRF1, and NRF2 (Fig. 6E). Meanwhile, PA treatment increased the levels of mitochondrial fusion proteins OPA1, MFN1 and MFN2, and decreased the levels of mitochondrial fission protein DRP1, while silencing SOGA1 decreased the levels of fusion proteins OPA1, MFN1 and MFN2, and increased the levels of mitochondrial fission protein DRP1 (Fig. 6F and G). These results suggested that SOGA1 knockdown may inhibit hepatocyte senescence by enhancing hepatocyte mitochondrial homeostasis through inhibiting PA-induced mitochondrial dynamics imbalance and mitochondrial biogenesis.

### Knockdown of SOGA1 inhibited hepatocyte senescence by enhancing mitophagy through the AMPK/mTOR signaling pathway

**Fig. 7:**
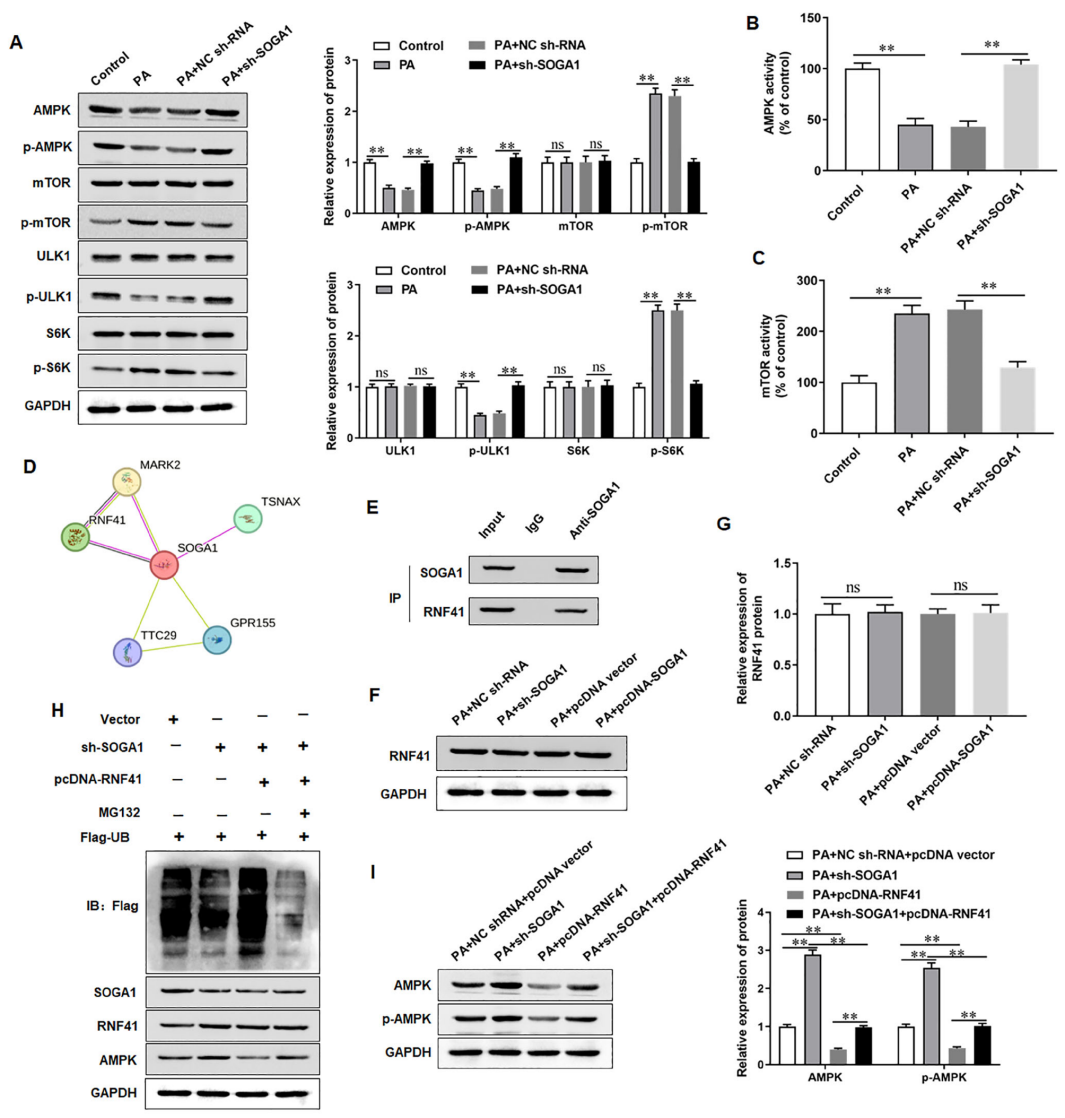
Knockdown of SOGA1 inhibited hepatocyte senescence by enhancing mitophagy through the AMPK/mTOR signaling pathway. NCTC1469 cells pretreated with 200 µM PA were transfected with NC shRNA or sh-SOGA1 for 48 h. **A**. The protein expression levels of AMPK, p-AMPK, mTOR, p-mTOR, ULK1, p-ULK1, S6K, and p-S6K were detected with Western blotting; The activity of AMPK (**B)** and mTOR (**C**) was detected with kits; D. STRING database was used to predict the interaction proteins of SOGA1; **E.** Interaction between SOGA1 and RNF41 proteins was verified by Co-IP analysis; **F** and** G.** RNF41 protein levels were detected with Western blotting; **H.** Ubiquitination levels of AMPK were assessed with Western blot. **I.** The protein levels AMPK and p-AMPK were detected with Western blotting. Data shown are the mean ± SD, sample size (N) = 5. The statistical differences were evaluated by one-way ANOVA, and followed by LSD test. Compared to control group, PA + NC sh-RNA group, PA+pcDNA vector group, PA + NC sh-RNA+pcDNA vector group, PA + sh-SOGA1 group, or PA+pcDNA-RNF41 group, ns *P* > 0.05, * *P* < 0.05, ** *P* < 0.01

Activation of AMP-dependent protein kinase (AMPK) and inhibition of mammalian target of rapamycin (mTOR) are the main mechanisms of autophagy activation. Wei et al.’s report [26] showed that SOGA1 promotes AMPKα1, β1, γ1, and p-AMPK ubiquitination degradation by binding to the AMPK catalytic subunits α, β, and γ, as well as phosphorylated AMPK (p-AMPK). Therefore, we explored the effect of SOGA1 silencing on the activation of AMPK/mTOR signaling pathway. The results showed that PA treatment reduced the protein levels of AMPK and p-AMPK, increased mTOR and S6K phosphorylation levels, and decreased ULK1 phosphorylation levels, but had no effect on total protein levels of mTOR, ULK1, and S6K. Silencing SOGA1 increased AMPK, p-AMPK, and p-ULK1 levels and decreased p-mTOR and p-S6K levels (Fig. 7A). Furthermore, SOGA1 silencing counteracted the inhibitory effect of PA on AMPK enzyme activity and its promotional effect on mTOR enzyme activity (Figs. 7B and C). Next, we predicted the interacting proteins of SOGA1 by using the STRING database and found that Ring Finger Protein 41 (RNF41), an E3 ubiquitin ligase, may interact with SOGA1 (Fig. 7D). Co-IP analysis revealed that the RNF41 protein was significantly enriched in the protein complex pulled down by the anti-SOGA1 antibody (Fig. 7E). Surprisingly, neither overexpression nor silencing of SOGA1 affected the protein level of RNF41 (Figs. 7F and G). Detection of AMPK ubiquitination levels showed that silencing SOGA1 reduced AMPK ubiquitination levels, while overexpression of RNF41 significantly promoted AMPK ubiquitination. The proteasome inhibitor MG132 reversed the AMPK ubiquitination induced by RNF41 overexpression (Fig. 7H). Additionally, overexpression of RNF41 counteracted the promoting effect of SOGA1 silencing on the protein levels of AMPK and p-AMPK (Fig. 7I). At the same time, we found that silencing RNF41 increased AMPK and p-AMPK protein levels and decreased p-mTOR protein levels (Supplementary Fig. 3A). Furthermore, silencing RNF41 increased the LC3-II/I ratio, decreased p62, p53, p21, γ-H2AX protein levels, and mRNA levels of SREBP-1c, ACC, and FATP2, increased mRNA levels of PPARα and CPT-1, and decreased collagen Ⅰ, Ⅲ, and α-SMA protein levels and JS1 cell viability (Supplementary Fig. 3B-F). Silencing RNF41 counteracted the effects of SOGA1 overexpression on hepatocyte senescence, fatty acid metabolism, autophagy, hepatic stellate cell activation, and fibrosis (Supplementary Fig. 3A-F). Therefore, we speculated that SOGA1 may promote the ubiquitin-mediated degradation of AMPK through the proteasome pathway by recruiting RNF41 in PA-treated hepatocytes.

To further confirm the role of the AMPK/mTOR pathway in SOGA1-mediated inhibition of autophagy in hepatocytes, SOGA1 silenced NCTC1469 cells were treated with compound C (CC), an AMPK inhibitor. The results showed that CC treatment significantly counteracted the promoting effect of SOGA1 silencing on the activation of the AMPK/mTOR pathway and the inhibitory effect on autophagy activation in PA-treated NCTC1469 cells (Supplementary Figs. 4 A and B).

### Knockdown of SOGA1 inhibited NASH progression in mice

**Fig. 8:**
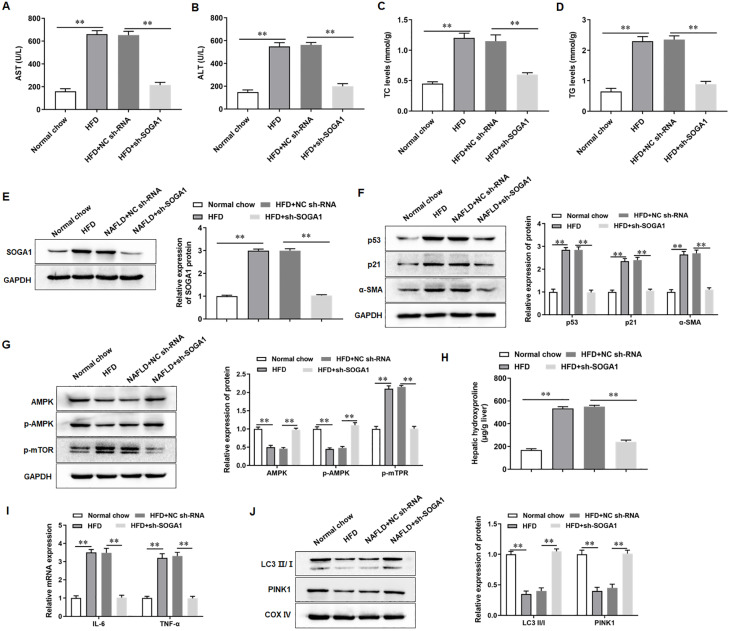
Knockdown of SOGA1 inhibited HFD-induced NASH progression in mice. Forty C57BL/6 mice were randomly divided into four groups, normal chow, HFD, HFD + NC shRNA, and HFD + sh-SOGA1 groups, with 10 mice in each group. After 8 weeks of feeding, all mice were euthanized, and blood and liver tissues were collected. **A** and **B**. The activities of AST and ALT in serum were detected with biochemical kits; **C** and **D**. The contents of TC and TG in serum were detected with biochemical kits; **E**. The protein level of SOGA1 in liver tissues was detected with Western blotting; **F**. The protein levels of p53, p21 and α-SMA in liver tissues were detected with Western blotting; **G**. The protein levels of AMPK, p-AMPK, and p-mTOR in liver tissues were detected with Western blotting; **H**. Hydroxyproline assay kit was used to detect the hydroxyproline level in liver tissues; **I**. The mRNA levels of IL-6 and TNF-α in liver tissues were detected with RT-qPCR; **J.** The protein levels of LC3-II/I and PINK1 in liver tissues were detected with Western blotting. Data shown are the mean ± SD, sample size (N) = 5. The statistical differences were evaluated by one-way ANOVA, and followed by LSD test. Compared to normal chow group or HFD + NC sh-RNA group, ** *P* < 0.01

**Fig. 9:**
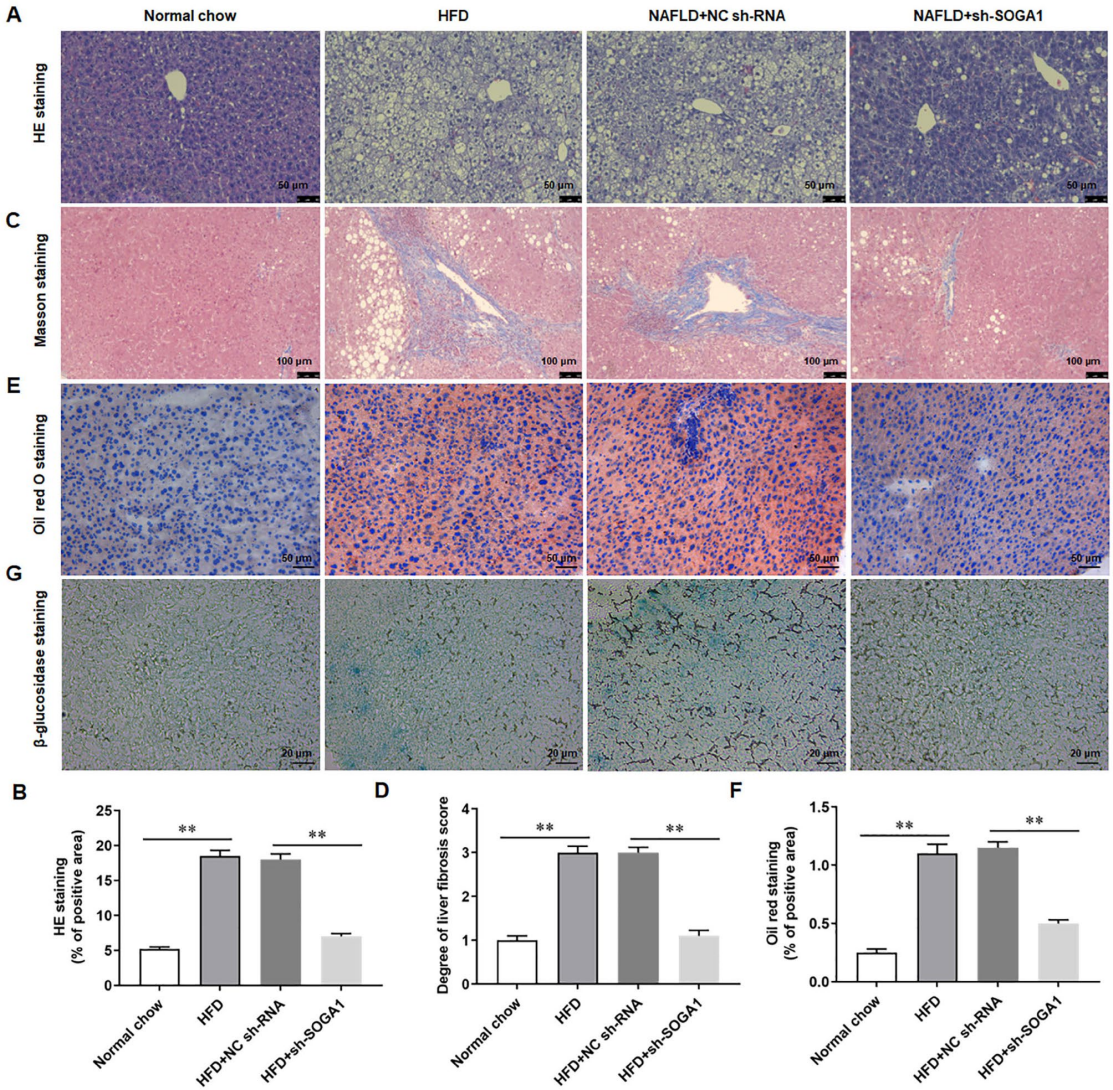
Knockdown of SOGA1 ameliorated liver histopathological damage in HFD-induced NASH mice with fibrosis. Forty C57BL/6 mice were randomly divided into four groups, normal chow, HFD, HFD + NC shRNA, and HFD + sh-SOGA1 groups, with 10 mice in each group. After 8 weeks of feeding, all mice were euthanized and liver tissues were collected for histopathological analysis. **A **and **B**. HE staining was used to observe the histopathological changes of mouse liver; **C **and** D.** Masson staining was used to observe liver fibrosis in mice; **E **and** F**. Oil red O staining was used to analyze the lipid deposition in liver tissues of mice; **G**. β-galactosidase staining was used to observe cellular senescence in liver tissues of mice. Data shown are the mean ± SD, sample size (N) = 5. The statistical differences were evaluated by one-way ANOVA, and followed by LSD test. Compared to normal chow group or HFD + NC sh-RNA group, ** *P* < 0.01

NASH mice with fibrosis were injected with lentiviral vectors of sh-SOGA1 via the tail vein to explore the effect of SOGA1 knockdown on the progression of NASH in mice. The results showed that compared with NASH mice injected with NC shRNA, knockdown of SOGA1 reduced AST and ALT activities, TC, and TG contents in the serum (Fig. 8A-D), as well as the protein level of SOGA1 in the liver tissue of NASH mice (Fig. 8E). HE staining results showed that knockdown of SOGA1 alleviated the pathological changes and reduced the infiltration of inflammatory cells and tissue necrosis in liver tissues of NASH mice (Fig. 9A and B). Masson’s staining showed that SOGA1 knockdown inhibited liver fibrosis in NASH mice (Fig. 9C and D). Oil Red O staining showed that knockdown of SOGA1 reduced lipid deposition in the liver tissue of NASH mice (Fig. 9E and F). The results of β-galactosidase staining showed that knockdown of SOGA1 reduced the intensity of senescence-related β-galactosidase positive staining in the liver tissue of NASH mice (Fig. 9G). Meanwhile, Western blotting results showed that SOGA1 knockdown reduced the protein levels of senescence-related genes p53, p21, and α-SMA in the liver tissue of NASH mice (Fig. 8F). In addition, SOGA1 knockdown increased the protein levels of AMPK, p-AMPK, LC3-II/I, and PINK1 in the liver tissue of NASH mice and decreased the levels of p-mTOR, hydroxyproline, and inflammatory factors IL-6 and TNF-α (Fig. 8G-J).

To further validate the involvement of SOGA1 in NASH progression through the regulation of AMPK/mTOR pathway-mediated mitochondrial autophagy, we treated SOGA1-knockdown NASH mice with autophagy inhibitor CQ and AMPK inhibitor CC, respectively. The results showed that compared with SOGA1-knockdown NASH mice, both CQ and CC treatment significantly increased the levels of AST, ALT, TC, TG in serum and levels of hydroxyproline and inflammatory factors IL-6 and TNF-α in liver tissues (Supplementary Fig. 5A-F). Additionally, CC treatment reduced AMPK and p-AMPK levels, increased p-mTOR levels, decreased the LC3-II/I ratio, mitochondrial membrane potential, and ATP production levels, and increased ROS production levels, but had no effect on SOGA1 protein levels. In contrast, CQ treatment reduced the LC3-II/I ratio in the liver tissue of NASH mice (Supplementary Figs. 6 A-E). Meanwhile, both CQ and CC treatment reversed the effects of SOGA1 knockdown on the expression levels of senescence marker proteins, fibrosis marker proteins, fatty acid metabolism key proteins, and inflammatory factors in the liver tissue of NASH mice (Supplementary Fig. 6F and G). In summary, these results indicated that SOGA1 knockdown inhibits cellular senescence and fibrosis and promotes fatty acid metabolism through AMPK/mTOR-mediated autophagy, thereby alleviating the inflammatory injury and fibrotic damage, as well as lipid deposition in liver tissues of NASH mice.

To investigate whether SOGA1 can be used as a marker of NASH patients in the clinic, we assessed the expression of SOGA1 in human liver tissues through single-cell sequencing in the Human Protein Atlas database and found that SOGA1 is primarily expressed in endothelial cells, smooth muscle cells, fibroblasts, macrophages, T cells, B cells, and hepatocytes in human liver tissue (Supplementary Fig. 7). Additionally, we assessed the correlation between disease severity in NAFLD patients and SOGA1 expression levels in their serum. The results showed that SOGA1 expression levels in serum were elevated in NAFL patients compared to healthy volunteers (controls), and further elevated in NASH patients (Supplementary Fig. 8A). Furthermore, SOGA1 expression levels in the serum of NAFLD patients increased with the progression of steatosis and fibrosis (Supplementary Fig. 8B and C).

Subsequently, we explored whether SOGA1 enters the circulation from cells via active secretion or extracellular vesicle transport pathway. Firstly, we isolated exosomes from the serum of healthy volunteers and NASH patients, and identified SOGA1 protein levels in serum, exosome and exosome-free serum. The results showed that SOGA1 in the serum of NASH patients was mainly enriched in exosomes compared with the serum without exosomes (Supplementary Fig. 8E). In addition, compared with healthy volunteers, the SOGA1 protein level in serum exosomes from NASH patients was significantly increased, while there was no significant change in the level of SOGA1 protein in serum without exosomes (Supplementary Fig. 8F). Therefore, we speculated that SOGA1 may enter the blood circulation system mainly through the extracellular vesicle secretion pathway, thereby regulating the progression of NASH. SOGA1 may have potential as a biological target for the treatment of NASH.

## Discussion

NASH is an inflammatory liver injury disease caused by excessive lipid deposition in the liver, which is often accompanied by metabolic disorders such as obesity, diabetes, dyslipidemia, and abnormal blood pressure [2, 3]. Dietary and lifestyle changes are recognized as non-drug treatment strategies; however, due to the complex pathogenesis of NASH, current drug treatment is mainly focused on its pathogenic factors, key links in the pathogenesis, and related metabolic disorders. Currently, there is a lack of specific drugs available. In clinical studies, common treatments for NASH include regulating glucose and lipid metabolism, protecting the liver, and anti-inflammatory effects [27]. With the rise of biological targeted therapies, the development of biological targets for NASH has attracted the attention of researchers. In this study, we found for the first time that SOGA1, a marker of NASH, was upregulated in the liver tissue of NASH mice with fibrosis and PA-induced hepatocytes. Mechanistic studies showed that knockdown of SOGA1 may activate mitophagy by enhancing the balance of mitochondrial dynamics mediated by AMPK/mTOR pathway, maintaining mitochondrial homeostasis, inhibiting hepatocyte senescence and hepatic stellate cell activation, thereby alleviating lipid accumulation, inflammation and fibrosis in liver tissues, and inhibiting NASH progression. SOGA1, also known as an autophagy inhibitor. Interestingly, a report showed that the mRNA level of SOGA1 was increased in liver tissues of obese male mice induced by bisphenol S [17]. Our study results are consistent with this finding that SOGA1 was significantly upregulated in the liver tissue of HFD-induced NASH mice with fibrosis and PA-induced hepatocytes. In addition, clinical data showed that compared with healthy volunteers (controls), NAFL patients had elevated SOGA1 protein levels in serum, and NASH patients had further elevated SOGA1 protein levels in serum. The above studies confirmed that SOGA1 may be involved in NASH progression.

NASH may progress to irreversible liver damage (fibrosis) and organ failure, and in some cases may develop into hepatocellular carcinoma (HCC) [28]. Liver fibrosis is the strongest predictor of long-term clinical outcomes in patients with NASH [29]. Lipotoxicity and damaged hepatocytes drives the progression of NASH. The inflammatory environment in the liver further activates hepatic stellate cells (HSCs), which is a key step in the development of fibrosis [30]. Liver fibrosis is driven by the activation and proliferation of HSCs. Normally, HSCs are quiescent, non-proliferating cells, and their activation leads to the synthesis and production of extracellular matrix (ECM) proteins. These activated HSCs also promote the secretion of pro-inflammatory cytokines to maintain an inflammatory environment and further promote a fibrotic environment [28]. As collagen deposition in liver tissue becomes more pronounced, the condition of NASH patients may have progressed to cirrhosis [29]. In this study, we found that in patients with NASH, SOGA1 mainly enters the blood circulation system through extracellular vesicle transport, and its protein levels increased with the progression of steatosis grade and fibrosis stage. Furthermore, previous reports shown that cellular senescence is a key factor in NASH progression. Senescent cells are unable to proliferate, but retain metabolic activity and communicate with surrounding cells, thereby transmitting senescence and inducing damage through senescence-associated phenotypes [20, 22, 31]. Some studies have suggested that hepatocyte senescence may be a central pathological mechanism for the progression ofNASH, which promotes intracellular fat accumulation, fibrosis, and inflammation, as well as the secretion of inflammatory mediators associated with senescence [20, 32]. HFD-induced lipotoxicity leads to hepatocyte damage and death, which in turn activates immune cells and stellate cells, leading to fibrosis and NASH progression [33, 34]. Zhou et al. reported that the secretion of senescent hepatic stellate cells promotes the malignant transformation of NASH for hepatocellular carcinoma [35]. Zhang et al. reported that inflammatory factors and fibrosis markers were significantly upregulated, and lipid accumulation was significantly increased in HFD-induced NASH mice [36]. Consistent with the above reports, our study found that lipid content was increased, and the levels of the fibrosis marker proteins α-SMA, COL1A1, and TGF-β1 were significantly increased in PA-treated hepatocytes. Moreover, the protein levels of the senescence markers p53, p21, and γ-H2AX were increased, and the number of β-galactosidase-positive cells was increased in PA-treated hepatocytes. We also found that CM derived from PA-treated hepatocytes increased the protein level of α-SMA, an activation marker of hepatic stellate cells, and the protein levels of the extracellular matrix components collagen I and III in a dose-dependent manner. In addition, some reports shown that in mild NAFLD, there is only a large accumulation of lipids and a small amount of pro-inflammatory factor secretion, without fibrosis [37–39]. This differs from our findings, which may be due to differences in modeling time and the dosage of HFD used, resulting in varying degrees of damage in NASH mice.

The inhibition of hepatocyte senescence has been reported to effectively alleviate NASH progression. For example, Park et al. reported that TTP activation inhibits cell senescence by downregulating PAI-1 and alleviating age-dependent hepatic steatosis [40]. Zhang et al. reported that the COX-2/SHE dual inhibitor PTUPB alleviated hepatocyte senescence through the SIRT1/PI3K/AKT/MTR pathway to restore autophagy, thereby inhibiting the progression of NAFLD in mice [41]. Our study showed that knockdown of SOGA1 decreased the expression of senescence related markers and inflammatory factors, reduced the number of β-galactosidase positive cells in PA-treated hepatocytes, and inhibited cell activation and fibrosis marker expression in hepatic stellate cells; IL-6 recombinant protein (rmIL-6) reversed the inhibitory effect of CM from SOGA1 knockdown hepatocytes on hepatic astrocyte activation. In vivo experiments further confirmed that knockdown of SOGA1 inhibited lipid deposition, fibrosis, and inflammatory factor secretion and reduced cellular senescence in the liver tissue of NASH mice with fibrosis, and this alleviating the progression of NASH. These results indicated that hepatocyte senescence is a key process in the progression of NASH, and inhibiting hepatocyte senescence may represent an effective approach to alleviate the progression of NASH.

Autophagy is a cell degradation process that is a key regulator of cellular energy production and stress relief [42]. Previous studies have shown that HFD causes dysregulation of autophagy in the liver [43]. The expression of autophagy-related genes was significantly reduced in NASH, and autophagy activity was inhibited. The ratio of LC3II/LC3I and the expression of the mitophagy protein Fundc1 were significantly decreased in PA-treated AML12 cells, while the autophagy substrate protein p62 was increased, indicating that autophagy was impaired [41]. Transmission electron microscopy revealed a significant reduction in the number of autophagosomes in the liver tissue of HFD-fed mice compared to that in standard diet-fed mice. In addition, the protein levels of LC3-II and ATG7 in the liver tissue of NASH mice were significantly decreased, and the level of p62 protein was increased [44]. The same phenomenon was observed in our study, where the ratio of LC3II/LC3I was decreased, the protein level of p62 was increased, mitochondria swelled with cristae disappeared, and autophagosomes was reduced in PA-induced hepatocytes. The same changes were also observed in the liver tissue of HFD-fed mice, that is, autophagy activity in NASH was inhibited. However, SOGA1 knockdown increased the ratio of LC3II/LC3I, reduced p62 protein levels, promoted autophagy activation, and alleviating the damage of liver in NASH mice with fibrosis. This is consistent with previous reports [44–46] that autophagy activation effectively alleviates NASH progression.

In addition, our study also found that knockdown of SOGA1 significantly promoted the co-localization of autophagy protein LC3B and mitochondria in PA-treated hepatocytes, suggesting that mitophagy may be involved in the occurrence and development of NASH. Mitophagy, a process that selectively removes damaged mitochondria, is essential for maintaining liver homeostasis [23]. Chen et al. found that the mitochondrial membrane potential was decreased and mitophagy was disordered in PA-treated hepatocytes and liver tissues of HFD-fed mice. While promoting mitophagy in hepatocytes inhibits NASH progression in mice [47]. Our results showed that SOGA1 knockdown reversed the inhibitory effect of PA treatment on mitophagy in hepatocytes. Mitochondria regulate each other through mitophagy, mitochondrial biogenesis, and mitochondrial dynamics balance to jointly maintain mitochondrial homeostasis and function [24, 25]. Our results found that knockdown of SOGA1 promoted mitochondrial fission, reduced mitochondrial fusion, improved PA-induced imbalance of mitochondrial dynamics in hepatocytes, partially counteracted PA-induced mitochondrial biogenesis in hepatocytes, activated mitophagy, thereby enhancing mitochondrial homeostasis in hepatocytes and alleviating hepatocyte injury. This is consistent with previous reports that improving the balance of mitochondrial dynamics and activating mitophagy cloud enhance mitochondrial homeostasis and improve mitochondrial function, and alleviating NASH progression. For example, Chen et al. reported that hesperetin alleviated NASH progression by promoting mitochondrial dynamics balance and activating mitophagy [48].

The AMPK/mTOR signaling pathway is the main mechanism of autophagy activation [49]. The report showed that SOGA1 promotes the ubiquitination and degradation of AMPK α1, β1, γ1, and p-AMPK by binding to AMPK catalytic subunits α, β, and γ, as well as phosphorylated AMPK [26]. In this study, SOGA1 was found to promote the ubiquitination and degradation of AMPK by recruiting RNF41. Silencing SOGA1 promoted autophagy in PA-treated hepatocytes by activating the AMPK/mTOR pathway. Our study is the first to confirm the involvement of SOGA1 in NASH progression through in vivo and in vitro experiments, and explained its molecular mechanism. However, our study has some limitations. Firstly, the molecular mechanism by which senescent hepatocytes promote hepatic stellate cell activation is not fully understood; Second, how activated hepatic stellate cells in turn affect hepatocytes is unclear; Third, whether the molecular regulation mechanism of SOGA1 is consistent across different stages of NAFLD, including simple hepatic steatosis, NASH, fibrosis and cirrhosis, which remain to be verified by subsequent studies; Finally, chloroquine and compound C may produce off target effects despite their established uses. Therefore, the evidence chain that SOGA1 promotes hepatocyte senescence by regulating mitophagy through the AMPK/mTOR signaling pathway remains to be further verified by genetic manipulation.

In summary, our study showed that SOGA1 as a biomarker of NASH, and its expression was upregulated in liver tissues of NASH mice with fibrosis and PA-induced hepatocytes. Knockdown of SOGA1 may reduce hepatocyte senescence and inhibit hepatic stellate cell activation by enhancing mitochondrial homeostasis through AMPK/mTOR-mediated mitophagy, thereby alleviating the progression of NASH. This study further improves the regulatory network of the pathogenesis of NASH and provides a new perspective for the development of specific targets for NASH.

## Electronic Supplementary Material

Below is the link to the electronic supplementary material.

Supplementary Material 1

Supplementary Material 2

## Data Availability

The data and materials used and analyzed during the current study are available from the corresponding author upon reasonable request.

## Abbreviations

HSCs: Hepatic stellate cells
SOGA1: Suppressor of glucose by autophagy
NAFLD: Nonalcoholic fatty liver disease
NAFL: Non-alcoholic simple fatty liver
NASH: Non-alcoholic steatohepatitis
NAS: NAFLD activity score
HFD: High-fat diet
PA: Palmitic acid
CM: Conditioned medium
CQ: Chloroquine
CC: Compound C
FBS: Fetal bovine serum
SA-β-gal: Senescence-Associated β-Galactosidase
PAGE: Polyacrylamide gradient gels
PVDF: Polyvinylidene difluoride
Co-IP: Co-immunoprecipitation
MMP: Mitochondrial membrane potential
ROS: Reactive Oxygen Species
DCFH-DA: 2’,7’-Dichlorodihydrofluorescein diacetate
ECM: Extracellular matrix
AMPK: AMP-activated protein kinase
ULK1: Unc-51 Like Autophagy Activating Kinase 1
S6K: p70 S6 Kinase
RNF41: Ring Finger Protein 41
AST: Aspartate Aminotransferase
ALT: Alanine Aminotransferase
TC: Total Cholesterol
TG: Triglycerides
α-SMA: Alpha-Smooth Muscle Actin
COL1A1: Collagen Type I Alpha 1 Chain
TGF-β1: Transforming Growth Factor-Beta 1
SREBP1c: Sterol Regulatory Element-Binding Protein 1c
ACC: Acetyl-CoA Carboxylase
PPARα: Peroxisome Proliferator-Activated Receptor Alpha
CPT-1: Carnitine Palmitoyltransferase 1
FATP2: Fatty Acid Transport Protein 2
PINK1: PTEN-induced putative kinase 1
TFAM: Transcription Factor A
NRF1: Nuclear Respiratory Factor 1
NRF2: Nuclear factor erythroid 2-related factor 2
OPA1: Optic Atrophy 1
MFN1: Mitofusin 1
MFN2: Mitofusin 2
DRP1: Dynamin-related protein 1
γ-H2AX: phosphorylated histone 2AX
SASP: Senescence-Associated Secretory Phenotype
TNF-α: Tumor necrosis factor-alpha
IL-6: Interleukin-6
