# Supplementary material for: Knockdown of suppressor of glucose by autophagy (SOGA1) alleviates the progression of non-alcoholic steatohepatitis (NASH) by reducing hepatocyte senescence through regulating AMPK/mTOR-mediated mitochondrial homeostasis
Source: BMC Biotechnol. 2026 May 23;26:90. doi: 10.1186/s12896-026-01162-w (PMC13410608; doi:10.1186/s12896-026-01162-w)
Supplement: Supplementary file 1 — Supplementary Material 1 [file 12896_2026_1162_MOESM1_ESM.docx]

**Supplementary Figure Legends**

**
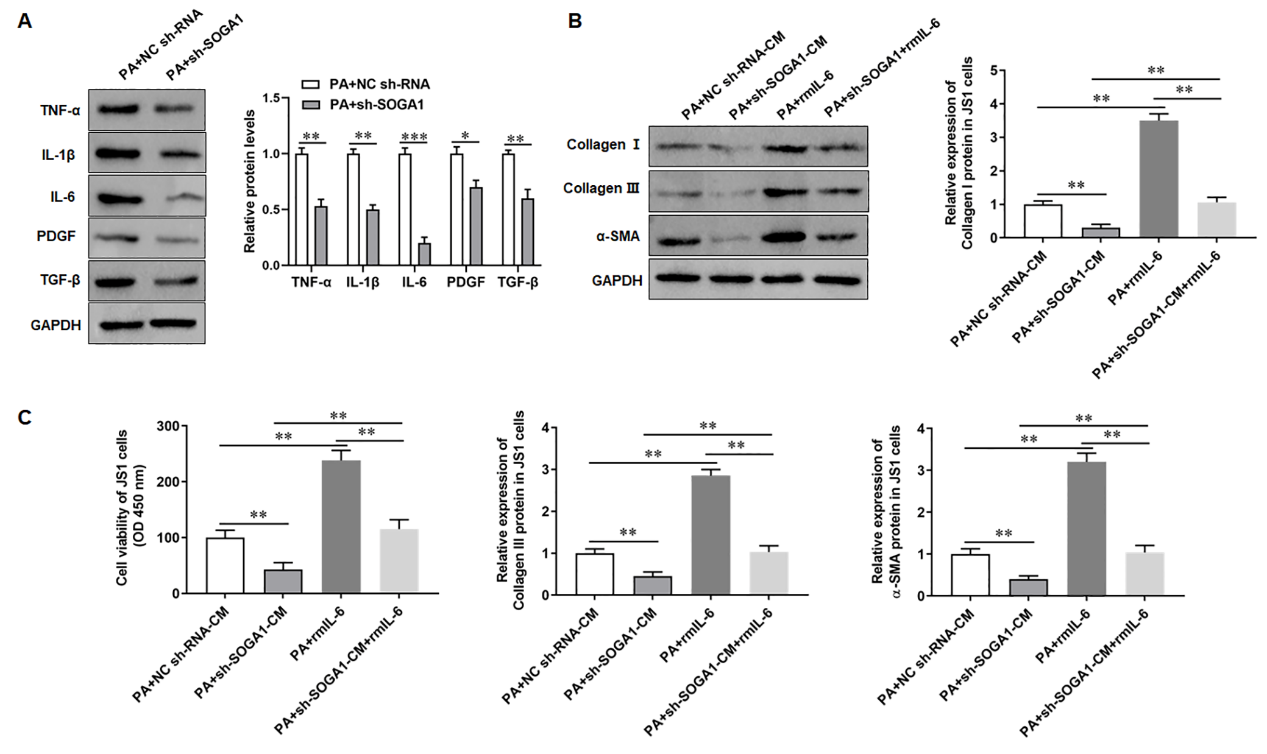
**

**Supplementary Figure 1. Knockdown of SOGA1 reduced hepatic stellate cell activation by inhibiting IL-6 secretion from senescent hepatocytes. A**. NCTC1469 cells pretreated with PA were transfected with NC shRNA and sh-SOGA1, and the protein levels of TNF-α, IL-1β, IL-6, PDGF, and TGF-β were detected with Western blotting. NCTC1469 cells pretreated with PA were transfected with NC shRNA and sh-SOGA1, and then the supernatant was isolated as CM to culture JS1 cells for 48 h. **B**. Western blotting was used to detect the protein levels of collagen Ⅰ, collagen Ⅲ and α-SMA; **C**. The viability of JS1 cells was detected with CCK-8. Data shown are the mean ± SD, sample size (N) = 5. CM: conditioned medium. The statistical differences were evaluated by one-way ANOVA, and followed by LSD test. Compared to PA+NC sh-RNA group, PA+NC sh-RNA-CM group, PA+sh-SOGA1-CM group, or PA+rmIL-6 group, * *P* <0.05, ** *P* <0.01.

**
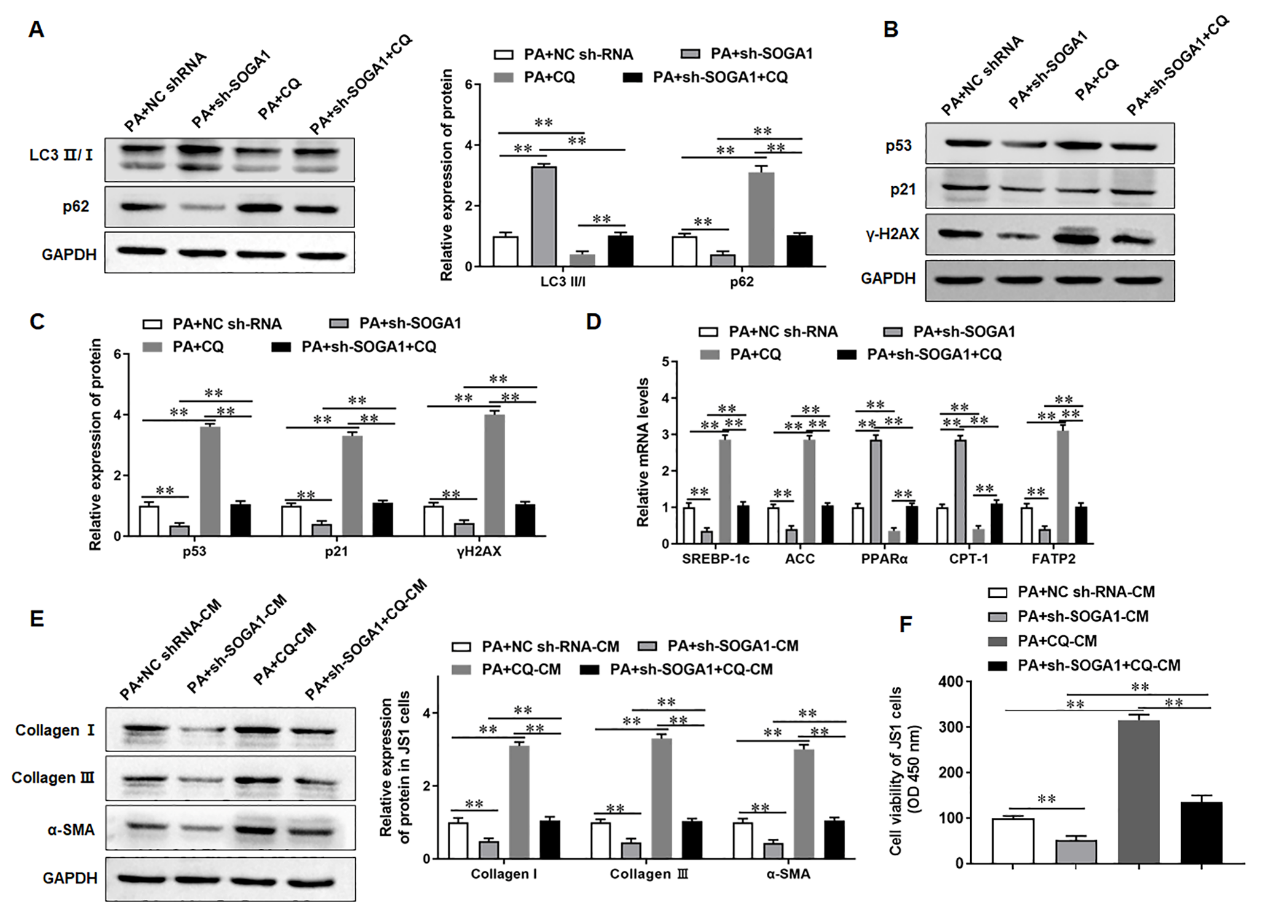
**

**Supplementary Figure 2. Knockdown of SOGA1 alleviated** **hepatic cell senescence** **and hepatic stellate cell activation by enhancing autophagy.** NCTC1469 cells pretreated with 200 μM PA were transfected with NC shRNA or sh-SOGA1 for 48 h, and then treated with 10 μM chloroquine (CQ) for 2 h. **A.** The protein levels of autophagy related proteins LC3-II/I and p62 were detected with Western blotting; **B and C**. The expression levels of senescence marker proteins p53, p21 and γ-H2AX were detected with Western blotting; **G.** The mRNA levels of fatty acid synthesis markers SREBP1c and ACC, fatty acid uptake marker FATP2, and fatty acid oxidation markers PPARα and CPT-1 were detected with RT-qPCR. NCTC1469 cells pretreated with 200 μM PA were transfected with NC shRNA or sh-SOGA1 for 48 h, and then treated with 10 μM chloroquine (CQ) for 2 h. Next, the supernatants of NCTC1469 cells were isolated as conditioned medium (CM) to culture JS1 cells for 48 h. **E.** The protein expression levels of collagen Ⅰ, collagen Ⅲ and α-SMA were detected with Western blotting. F. The viability of JS1 cells were detected with CCK-8. Data shown are the mean ± SD, sample size (N) = 5. The statistical differences were evaluated by one-way ANOVA, and followed by LSD test. Compared to PA+NC shRNA group, PA+sh-SOGA1 group, PA+CQ group, PA+NC shRNA-CM group, PA+sh-SOGA1-CM group, or PA+CQ-CM group, * *P* <0.05, ** *P* <0.01.


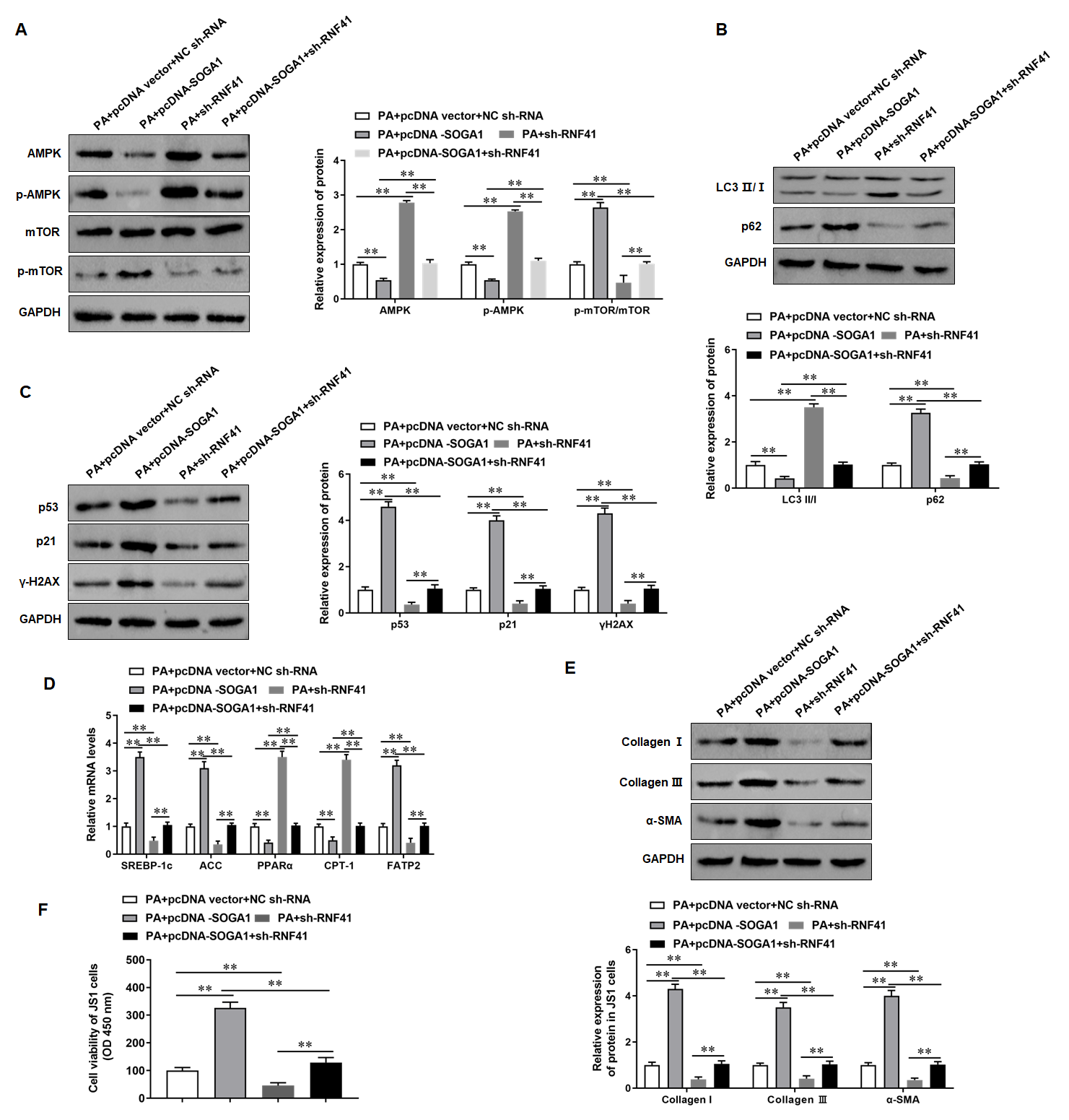


**Supplementary Figure 3. SOGA1** **inhibited hepatocyte senescence and hepatic stellate cell activation through RNF41 regulated AMPK/mTOR pathway.** NCTC1469 cells pretreated with 200 μM PA were transfected with pcDNA vector + NC shRNA, pcDNA-SOGA1, or/and sh-RNF41 for 48 h. **A**. The protein expression levels of AMPK, p-AMPK, mTOR, and p-mTOR were detected with Western blotting; **B**. The protein levels of autophagy related proteins LC3-II/I and p62 were detected with Western blotting; **C**. The protein expression levels of p53, p21 and γ-H2AX were detected with Western blotting; **D.** The mRNA levels of fatty acid synthesis markers SREBP1c and ACC, fatty acid uptake marker FATP2, and fatty acid oxidation markers PPARα and CPT-1 were detected with RT-qPCR. NCTC1469 cells pretreated with PA were transfected with pcDNA-SOGA1 or/and sh-RNF41, and then the supernatant was isolated as CM to culture JS1 cells for 48 h. **E**. Western blotting was used to detect the protein levels of collagen Ⅰ, collagen Ⅲ and α-SMA; **F**. The viability of JS1 cells was detected with CCK-8. Data shown are the mean ± SD, sample size (N) = 5. The statistical differences were evaluated by one-way ANOVA, and followed by LSD test. Compared to PA+pcDNA vector+NC shRNA group, PA+pcDNA-SOGA1 group, or PA+sh-RNF41 group, * *P* <0.05, ** *P* <0.01.


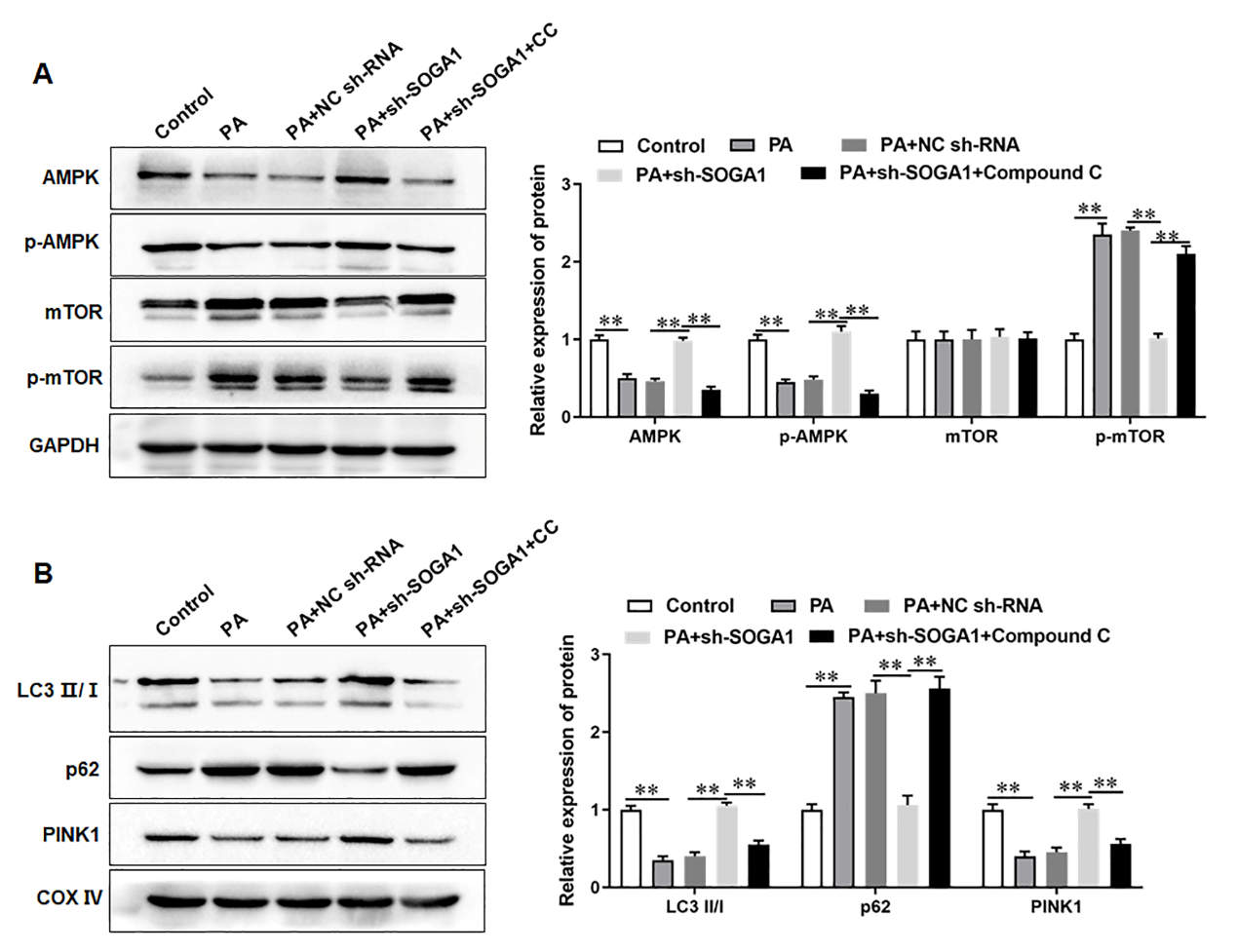


**Supplementary Figure 4. Knockdown of SOGA1 promoted autophagy in hepatocytes through the AMPK/mTOR signaling pathway.** NCTC1469 cells pretreated with 200 μM PA were transfected with NC shRNA or sh-SOGA1 for 48 h, and then treated with compound C for another 2 h. **A.** The protein expression levels of AMPK, p-AMPK, mTOR, and p-mTOR were detected with Western blotting; **B.** The protein levels of autophagy related proteins LC3-II/I and p62 were detected with Western blotting. Data shown are the mean ± SD, sample size (N) = 5. The statistical differences were evaluated by one-way ANOVA, and followed by LSD test. Compared to control group, PA group, PA+NC shRNA group, or PA+sh-SOGA1 group, * *P* <0.05, ** *P* <0.01.


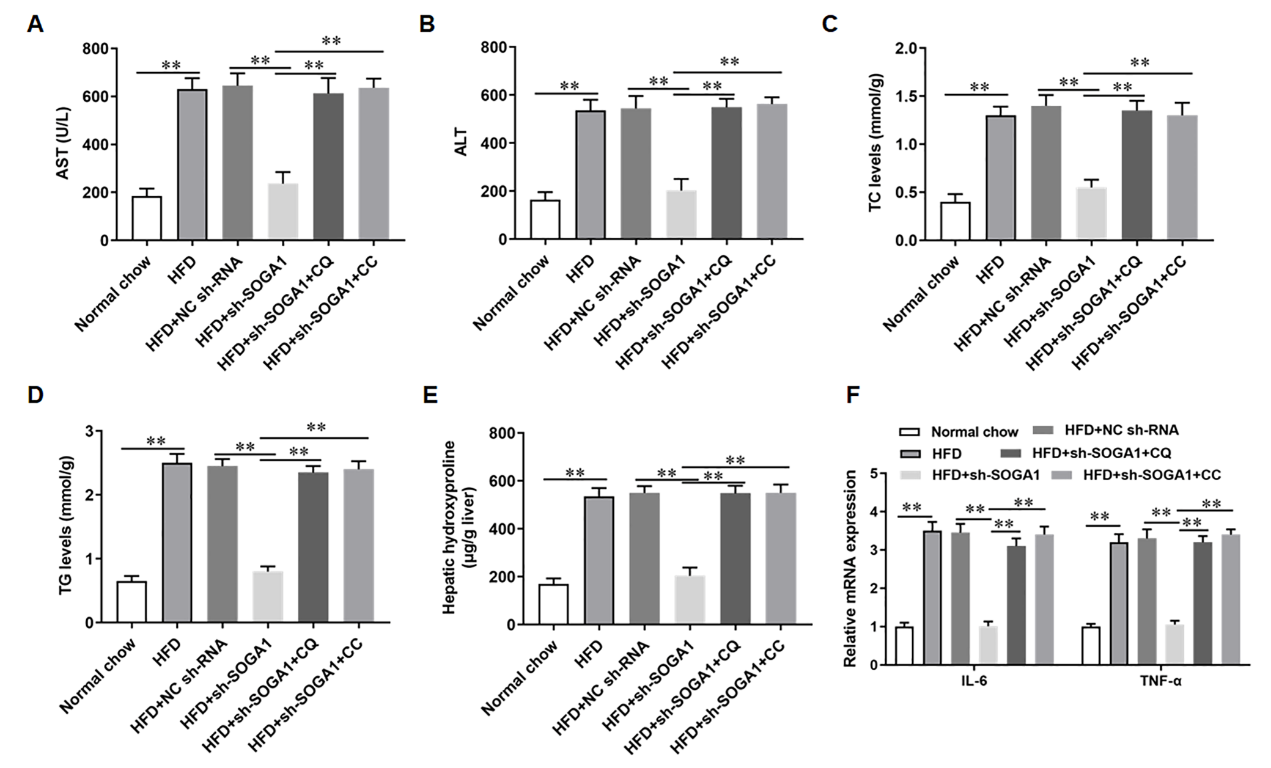


**Supplementary Figure 5. Knockdown of SOGA1 inhibited liver damage in NAFLD mice by promoting AMPK/mTOR-mediated autophagy.** Forty-eight mice were randomly divided into six groups: Normal chow, HFD, HFD+NC shRNA, HFD+sh-SOGA1, HFD+sh-SOGA1+CQ, and HFD+sh-SOGA1+CC, with 8 mice in each group. After 8 weeks of feeding, all mice were euthanized, and blood and liver tissues were collected. **A** and **B**. The activities of AST and ALT in serum were detected with biochemical kits; **C** and **D**. The contents of TC and TG in serum were detected with biochemical kits; **E**. Hydroxyproline assay kit was used to detect the hydroxyproline level in liver tissues; **F.** The mRNA levels of IL-6 and TNF-α in liver tissues of mice were detected with RT-qPCR. Data shown are the mean ± SD, sample size (N) = 5. The statistical differences were evaluated by one-way ANOVA, and followed by LSD test. Compared to normal chow group, HFD group, HFD+ NC shRNA group, or HFD+sh-SOGA1 group, * *P* <0.05, ** *P* <0.01.

**
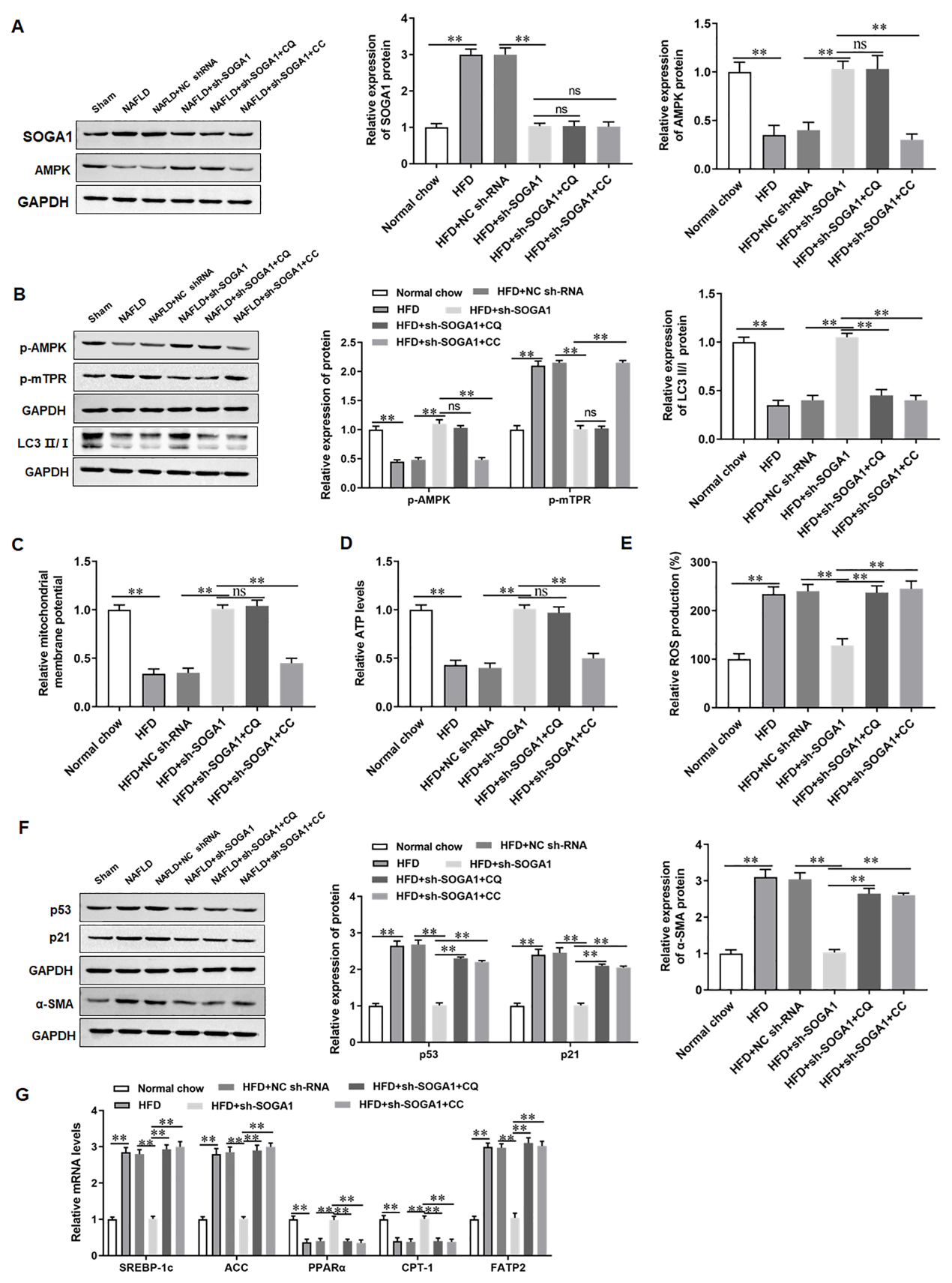
**

**Supplementary Figure 6. Knockdown of SOGA1 inhibited hepatic cell senescence and hepatic stellate cell activation in NAFLD mice through** **promoting AMPK/mTOR-mediated autophagy.** Forty-eight mice were randomly divided into six groups: Normal chow, HFD, HFD+NC shRNA, HFD+sh-SOGA1, HFD+sh-SOGA1+CQ, and HFD+sh-SOGA1+CC, with 8 mice in each group. After 8 weeks of feeding, all mice were euthanized, and blood and liver tissues were collected. **A and B.** The protein expression levels of SOGA1, AMPK, p-AMPK, mTOR, p-mTOR, and LC3-II/I were detected with Western blotting; **C.** The mitochondrial membrane potential (MMP) was measured by flow cytometry; **D.** ATP levels were measured by ATP bioluminescent assay kits; **E.** ROS production levels were measured by flow cytometry; **F.** The expression levels of senescence marker proteins p53, p21 and α-SMA were detected with Western blotting; **G.** The mRNA levels of SREBP1c, ACC, FATP2, PPARα, and CPT-1 were detected with RT-qPCR. Data shown are the mean ± SD, sample size (N) = 5. The statistical differences were evaluated by one-way ANOVA, and followed by LSD test. Compared to normal chow group, HFD group, HFD+ NC shRNA group, or HFD+sh-SOGA1 group, ns *P*>0.05, * *P* <0.05, ** *P* <0.01.


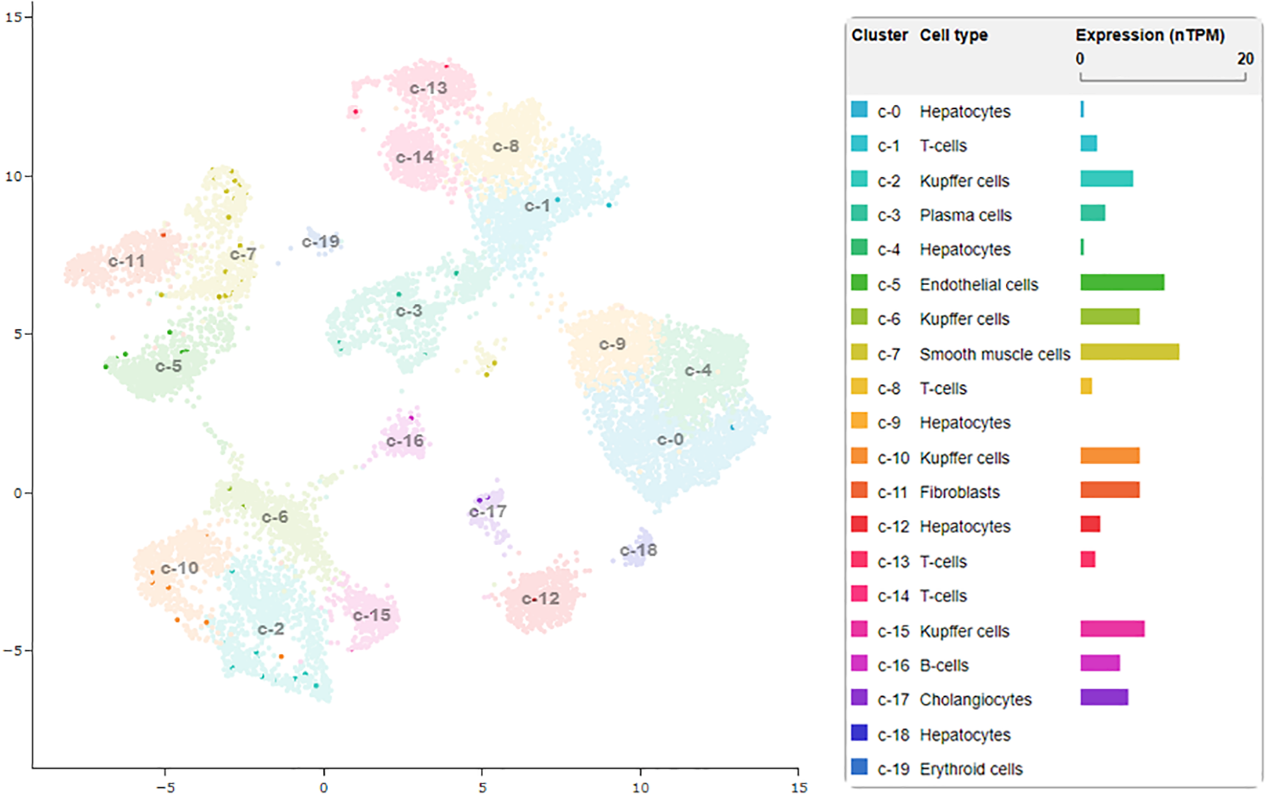


**Supplementary Figure 7. The expression of SOGA1 in human liver tissues was evaluated** **through single-cell sequencing in the Human Proteome Organization database.**

**
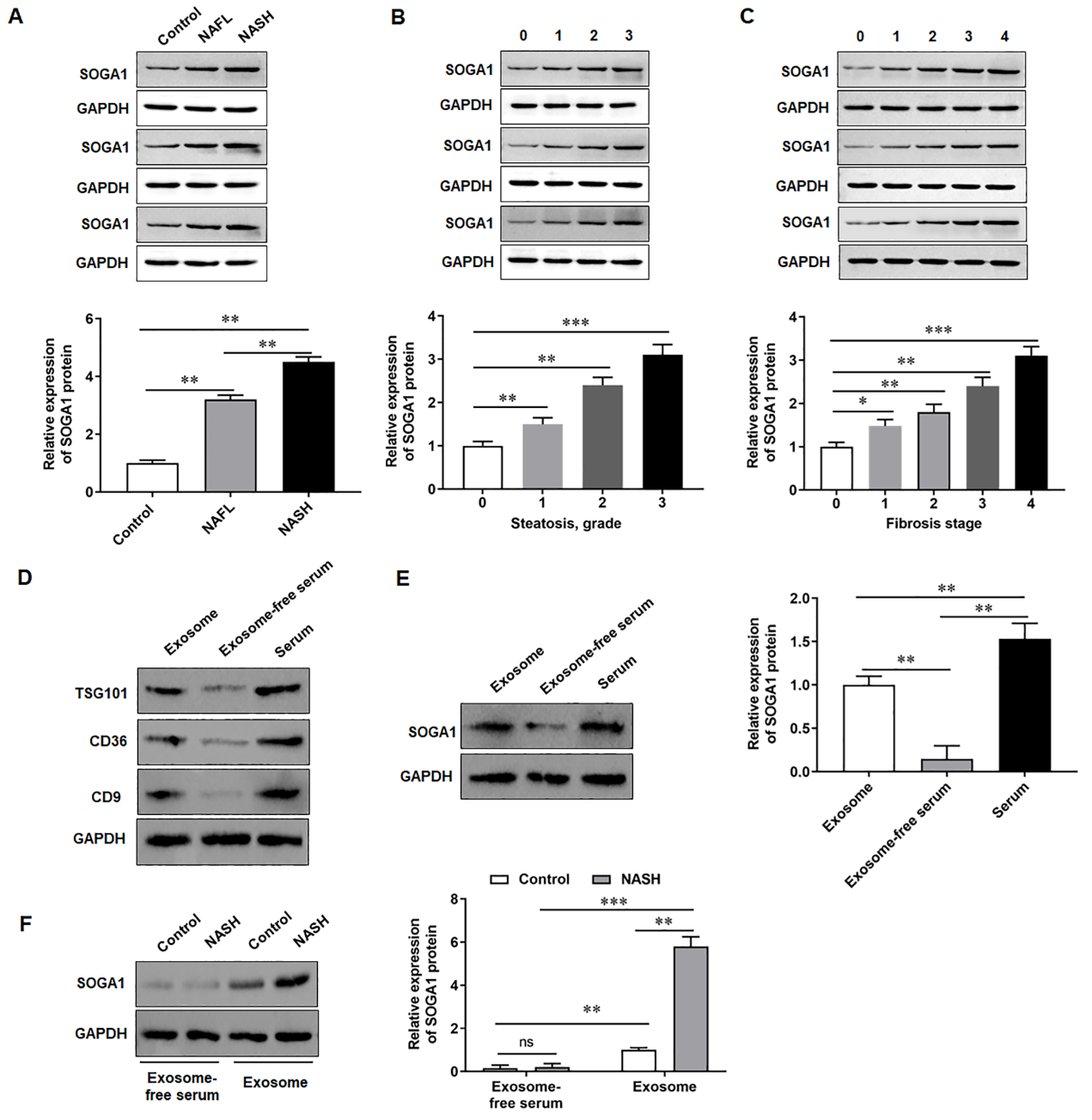
**

**Supplementary Figure 8. The expression of SOGA1 in NAFLD patients with varying degrees of disease severity. A.** The protein levels of SOGA1 in the serum of healthy volunteers (control, N = 48) and NAFLD patients with non-Alcoholic Fatty Liver (NAFL, N = 45) and non-Alcoholic SteatoHepatitis (NASH, N = 51) were detected with Western blotting; **B.** The protein levels of SOGA1 in the serum of NAFLD patients with different steatosis grades were detected with Western blotting; **C.** The protein levels of SOGA1 in the serum of NAFLD patients with different fibrosis stage were detected with Western blotting; **D.** The protein levels of TSG101, CD36 and CD9 were detected with Western blot; **E.** The protein level of SOGA1 in exosome, exosome-free serum, and serum from NASH patients were detected with Western blotting; **F.** The protein level of SOGA1 in exosome and exosome-free serum from healthy volunteers and NASH patients were detected with Western blotting. Data shown are the mean ± SD, sample size (N) = 5. The statistical differences were evaluated by one-way ANOVA, and followed by LSD test. Compared to control group, NAFL group, exosome group, or exosome-free serum group, ns *P*>0.05, * *P* <0.05, ** *P* <0.01, *** *P* <0.001.
