# Supplementary material for: Knockdown of suppressor of glucose by autophagy (SOGA1) alleviates the progression of non-alcoholic steatohepatitis (NASH) by reducing hepatocyte senescence through regulating AMPK/mTOR-mediated mitochondrial homeostasis
Source: BMC Biotechnol. 2026 May 23;26:90. doi: 10.1186/s12896-026-01162-w (PMC13410608; doi:10.1186/s12896-026-01162-w)

Figure 1H

α-SMA

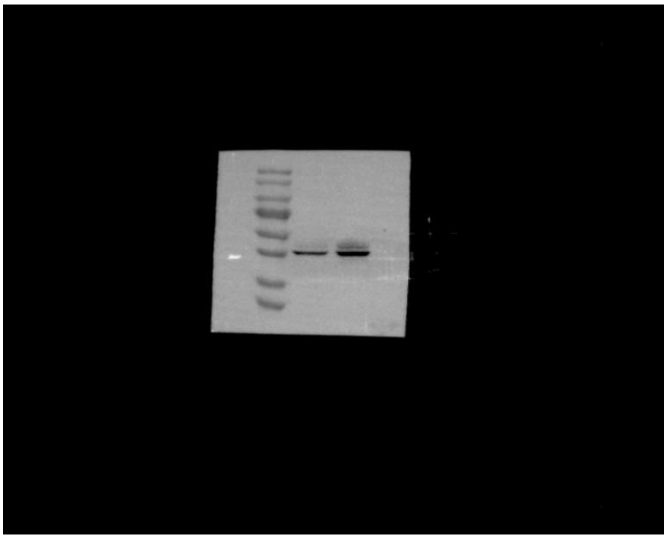

COL1A1

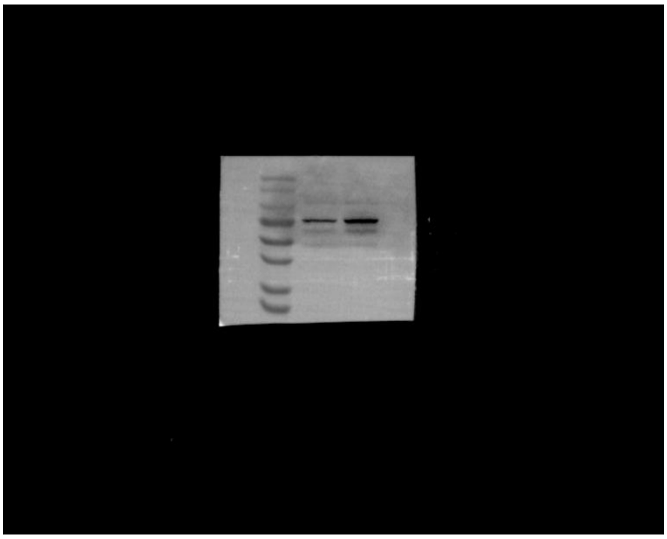

TGF-β1

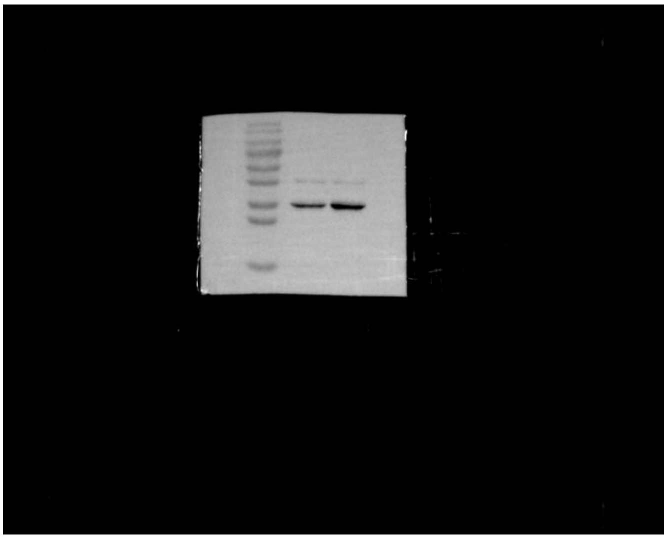

Figure 1I
SOGA1

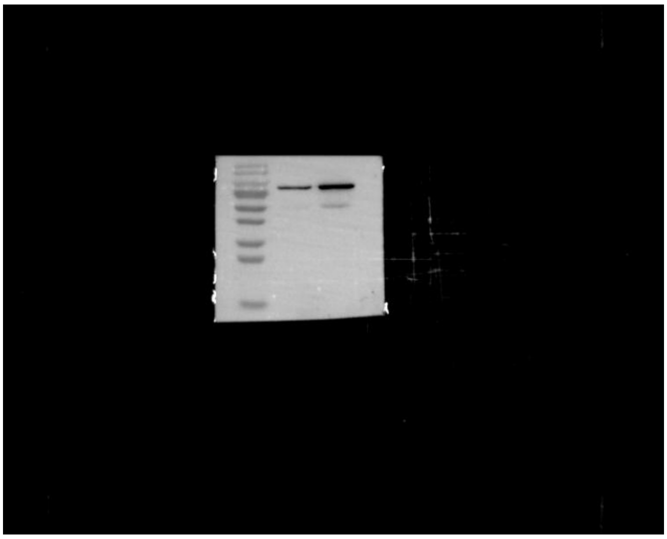

Figure 2A
SOGA1

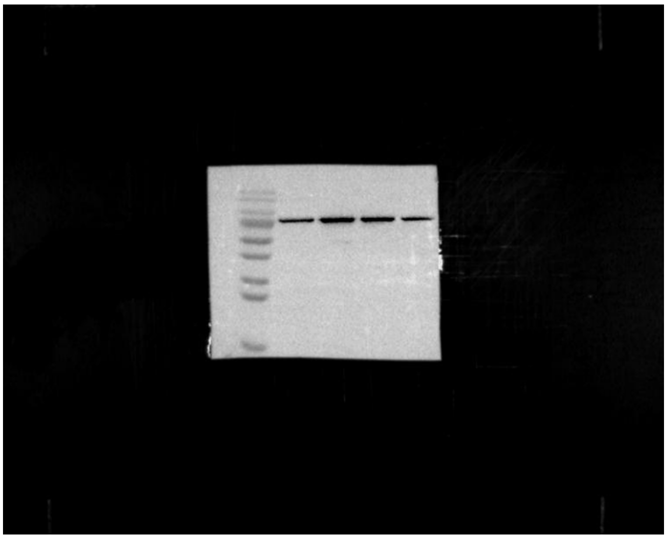

Figure 2E

α-SMA

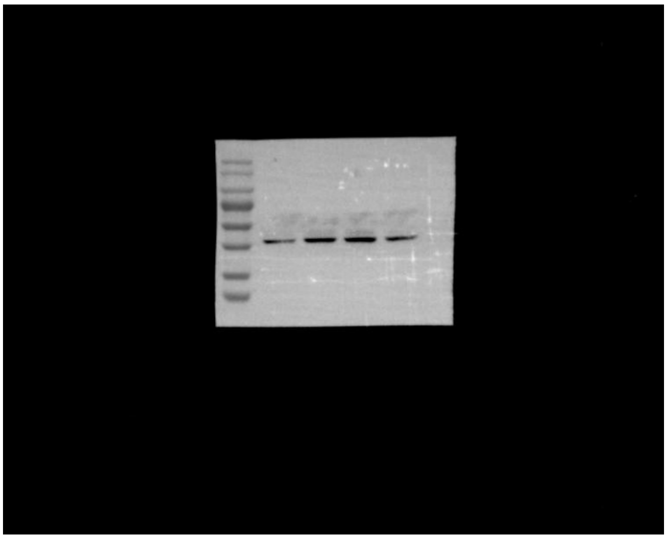

COL1A1

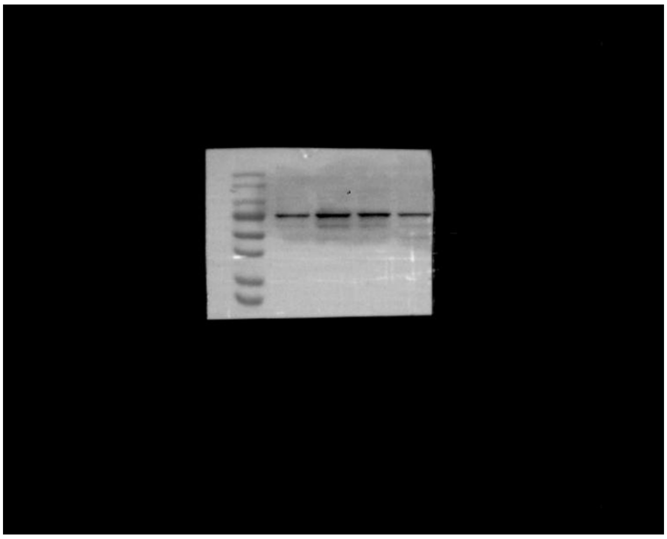

TGF-β1

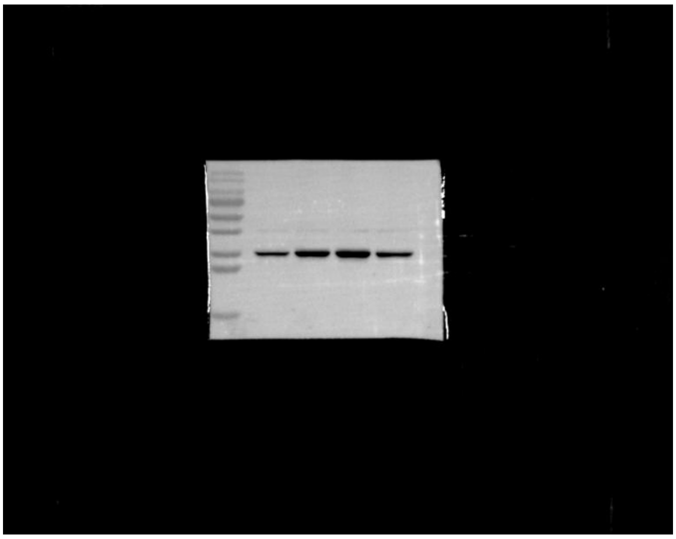

Figure 2G

SREBP1c

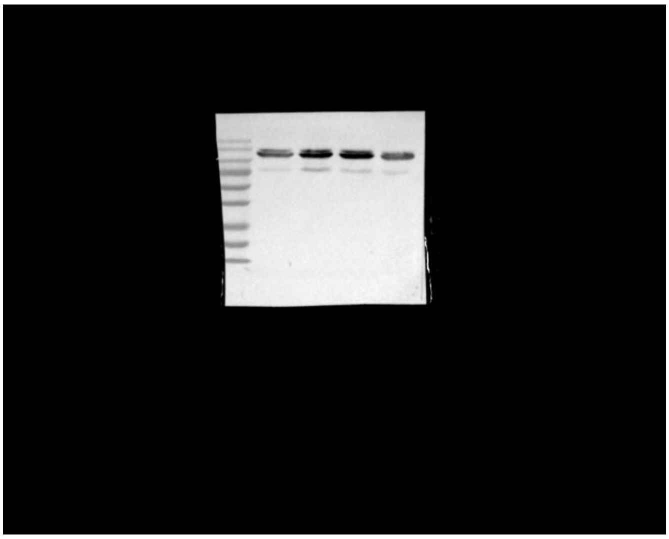

ACC

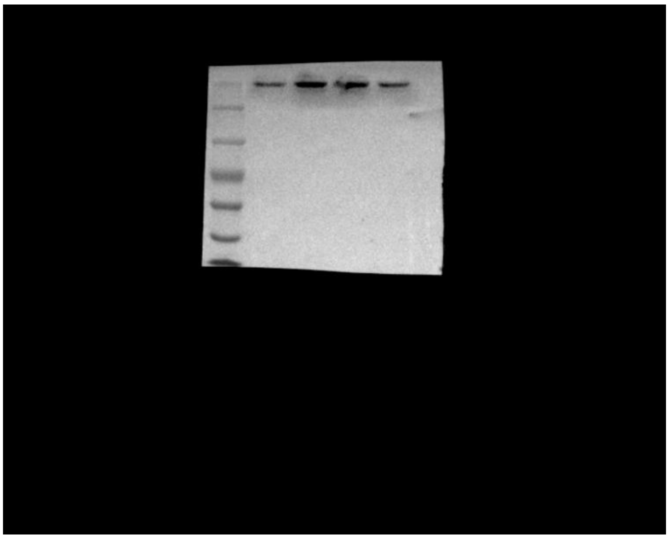

PPARα

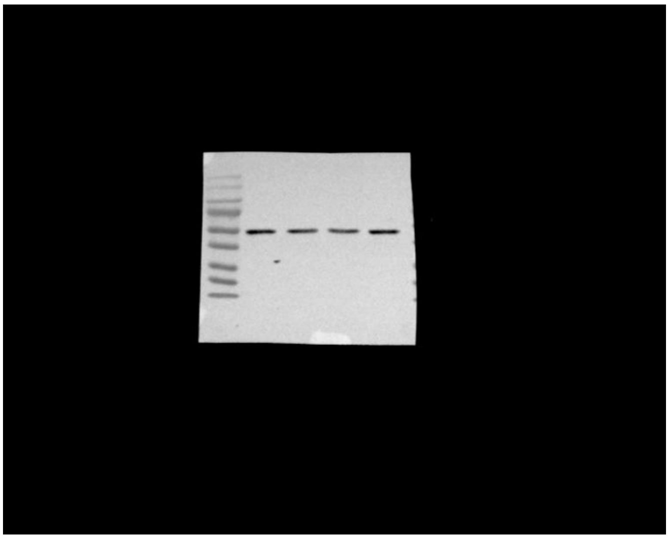

CPT-1

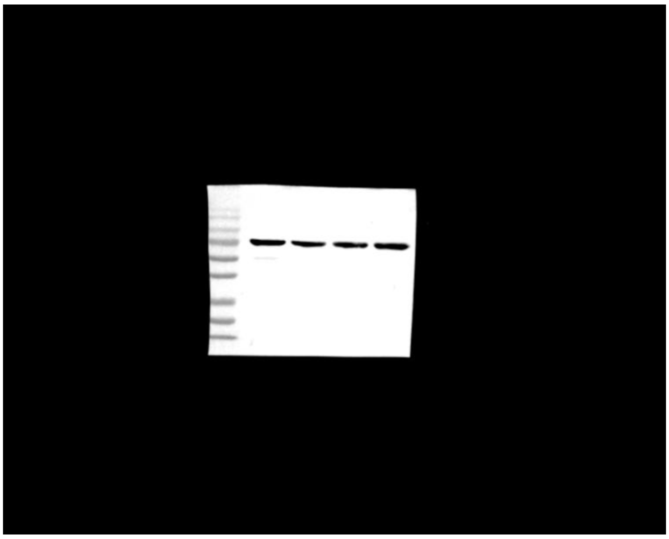

FATP2

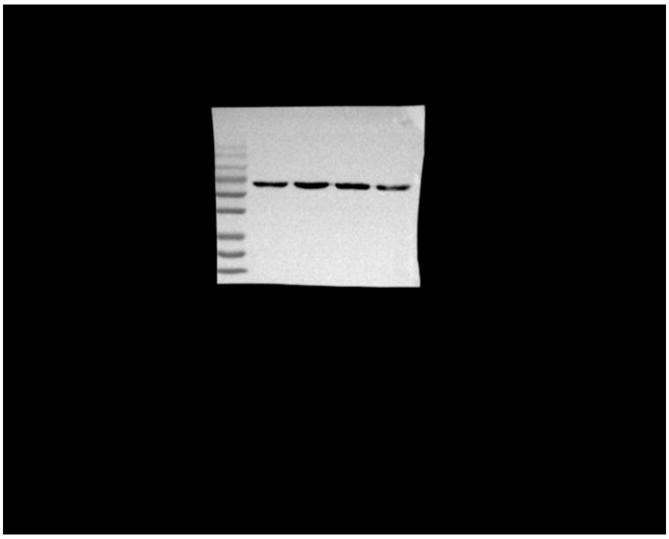

GAPDH

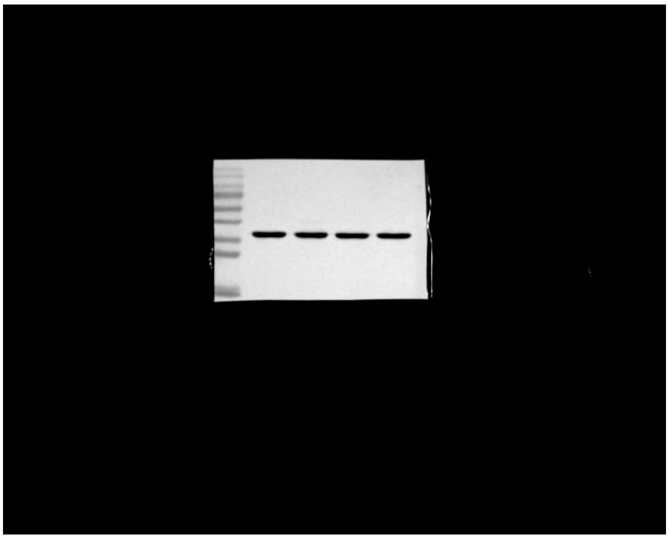

Figure 3A

P53

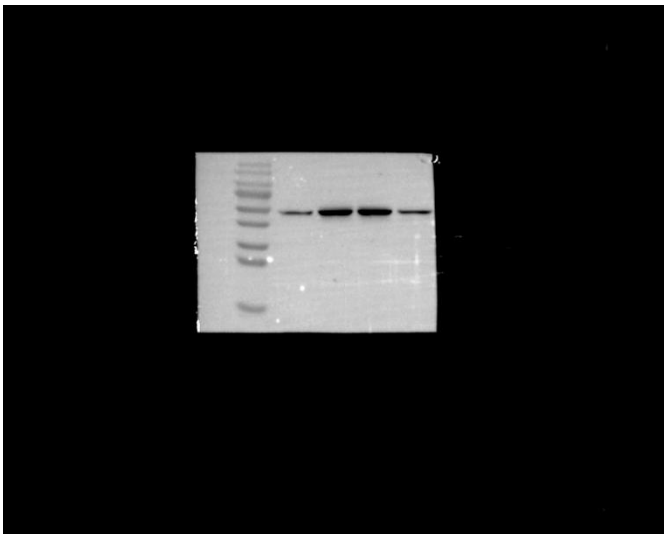

P21

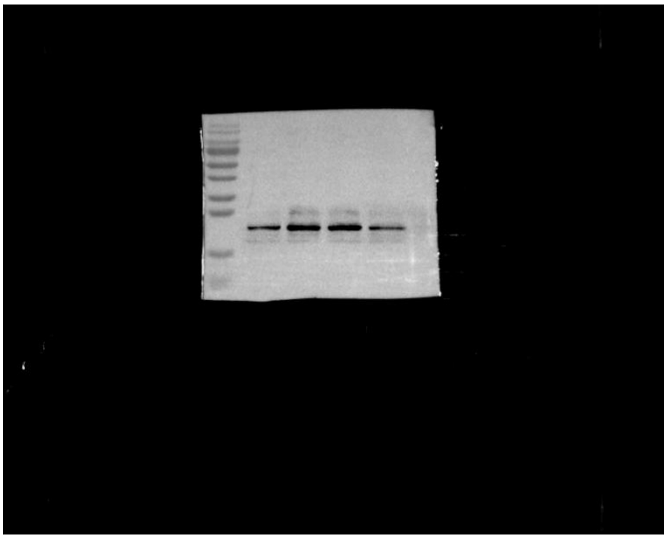

γ-H2AX

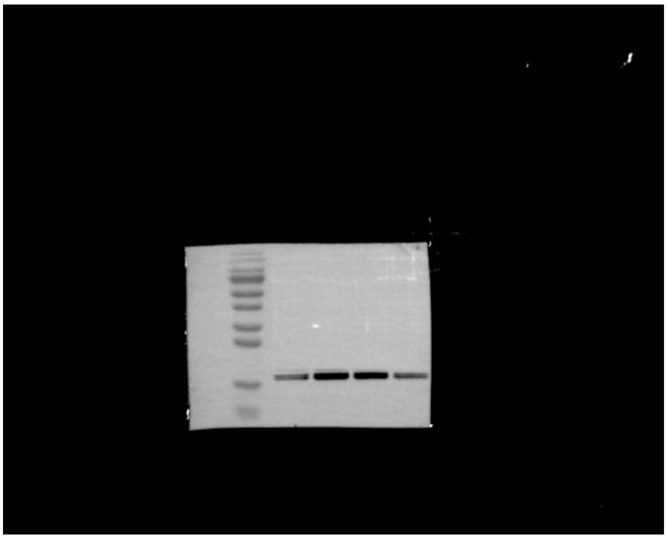

Figure 4A

Collagen I

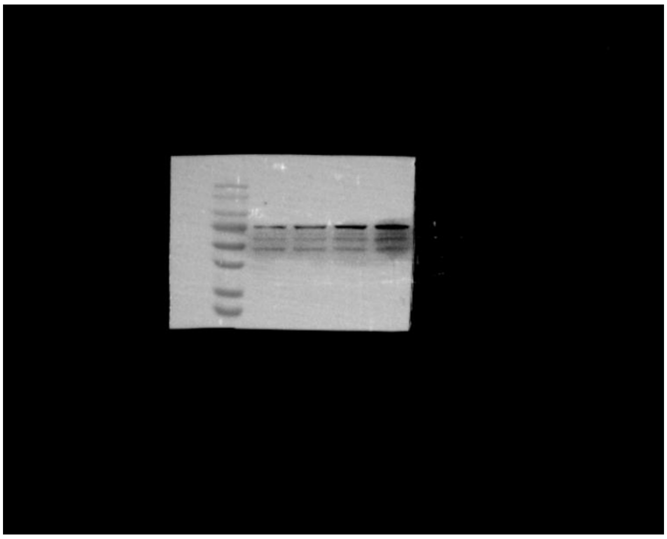

Collagen III

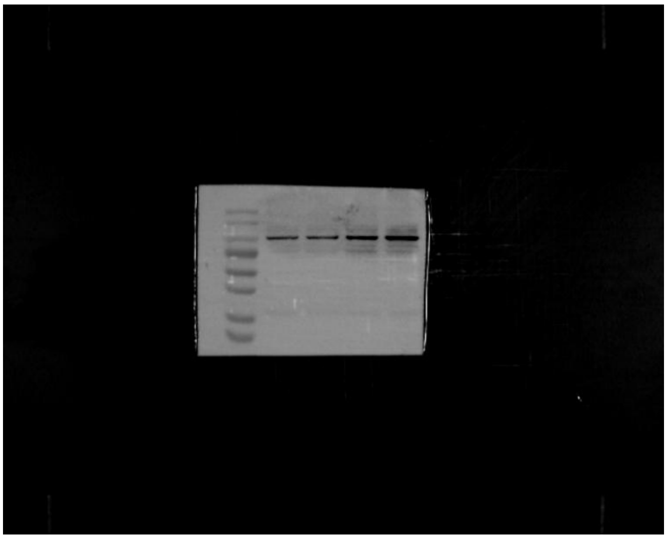

α-SMA

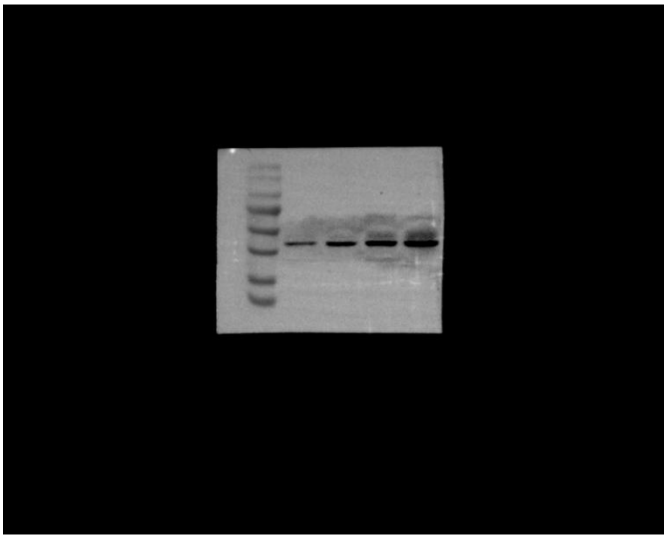

Figure 4E

Collagen I

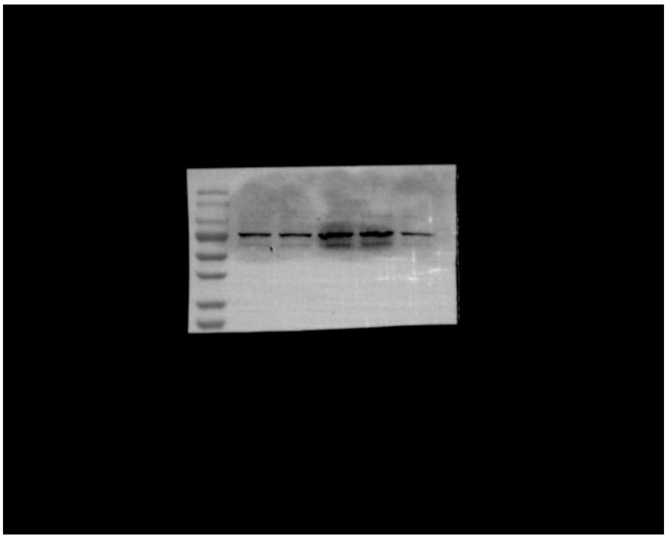

Collagen III

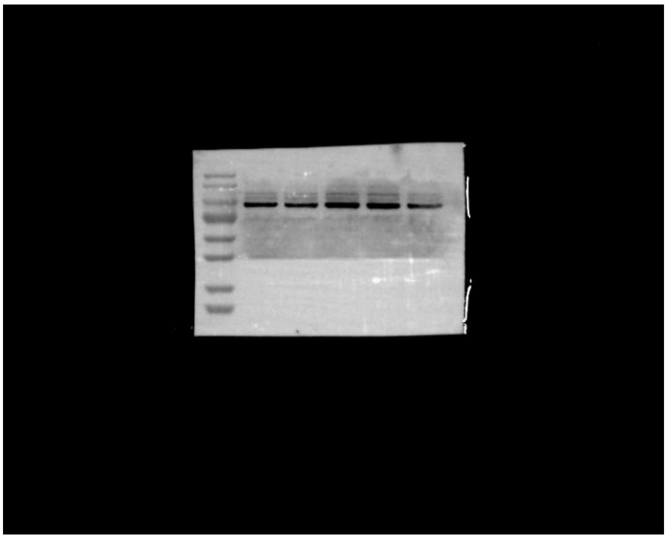

α-SMA

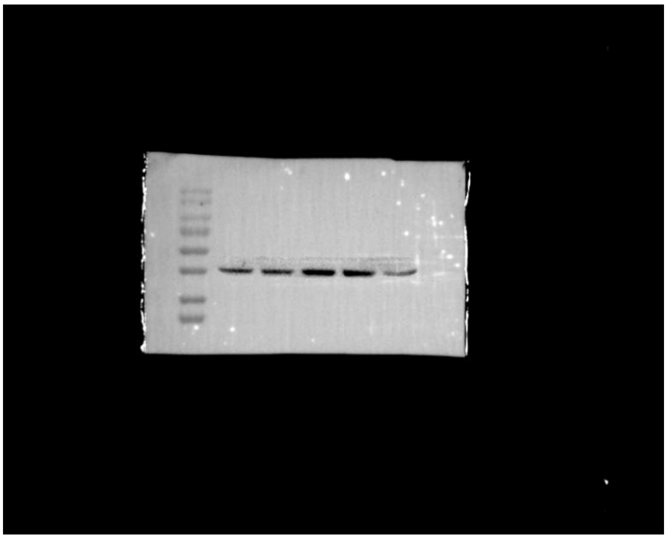

Figure 5A

LC3 II/I

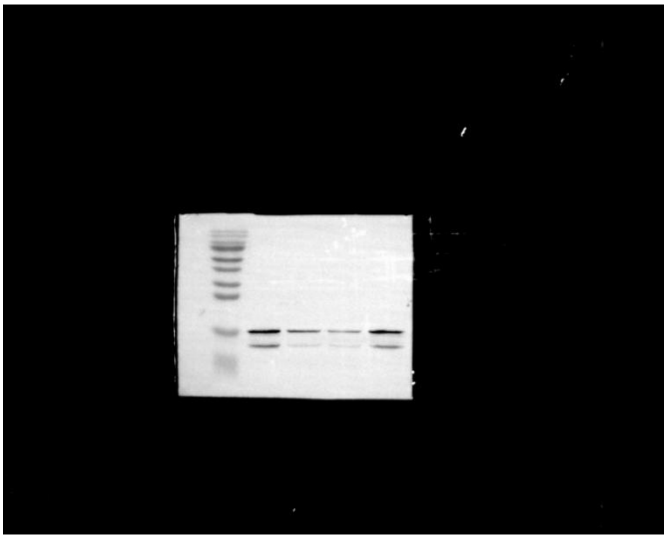

P62

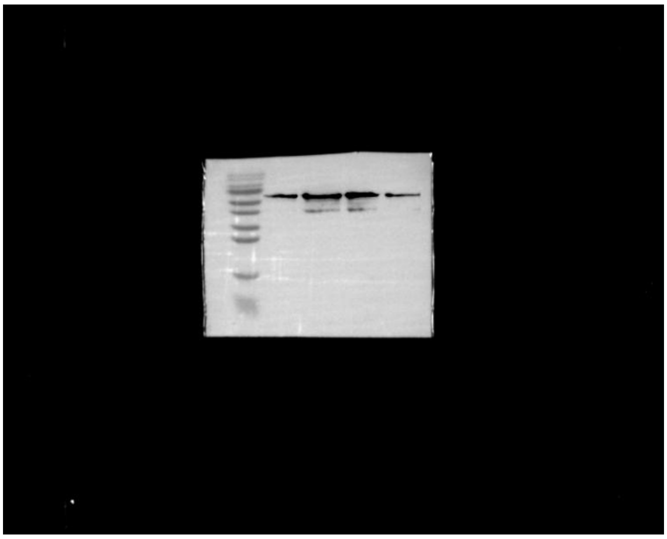

Figure 6D

PINK1(Mito)

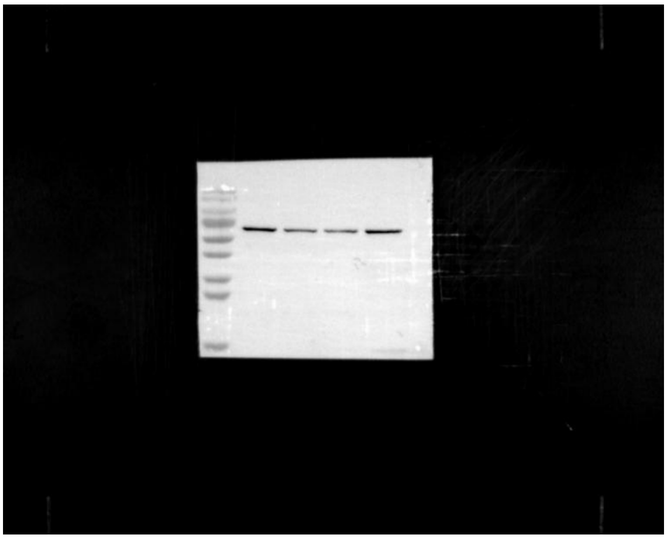

PINK1(Cyto)

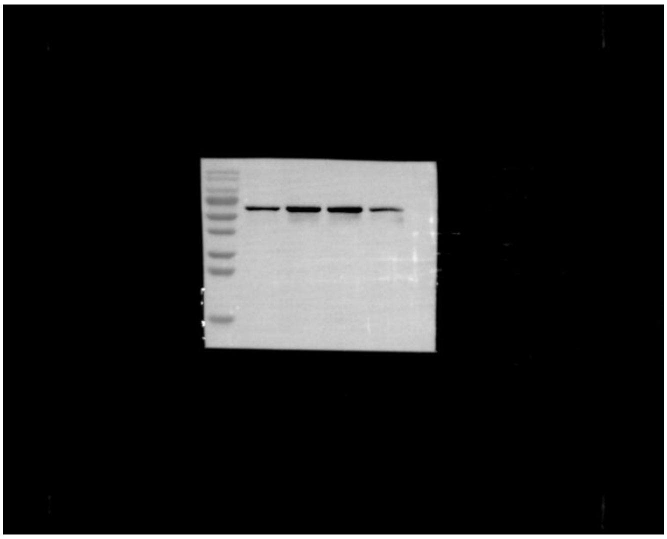

Figure 6E

TFAM

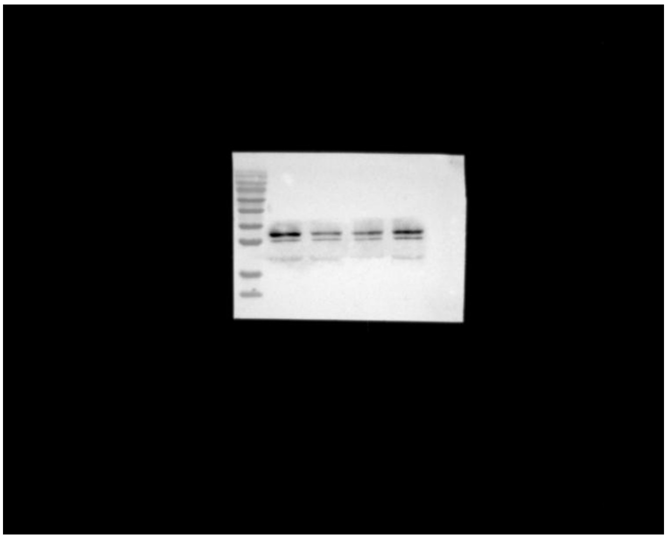

NRF1

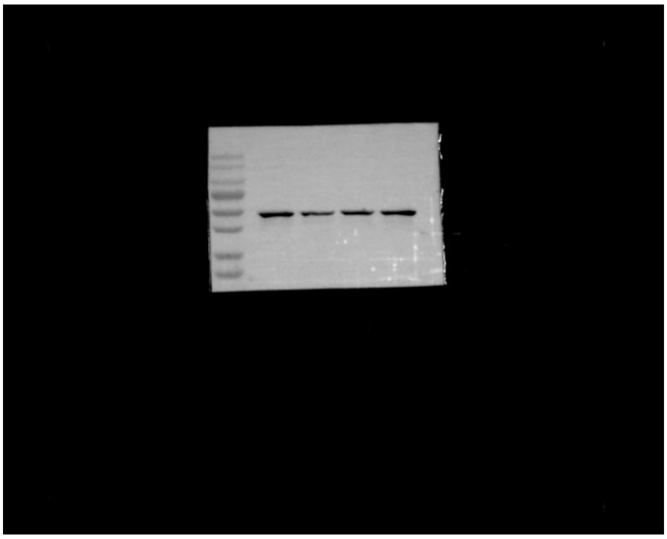

NRF2

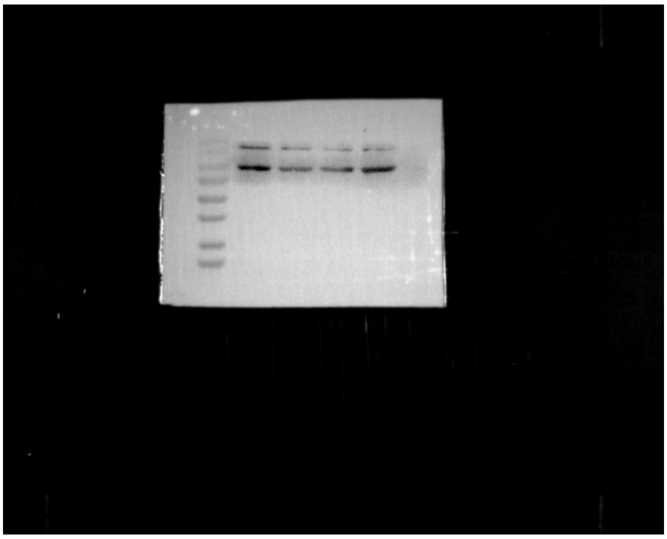

Figure 6F

OPA1

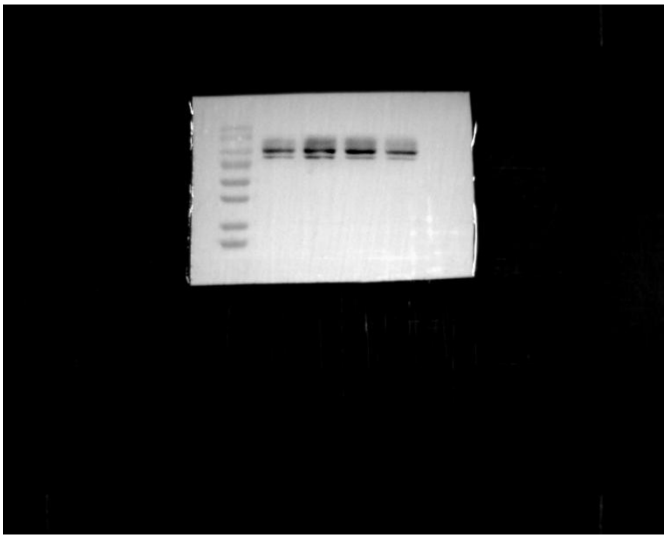

MFN1

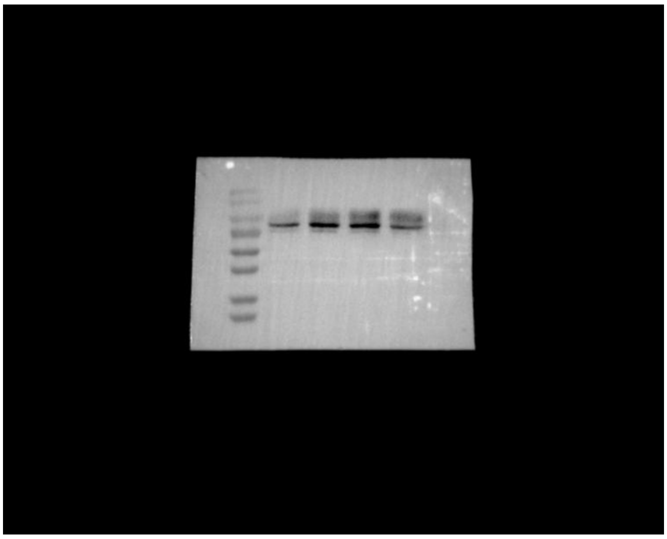

MFN2

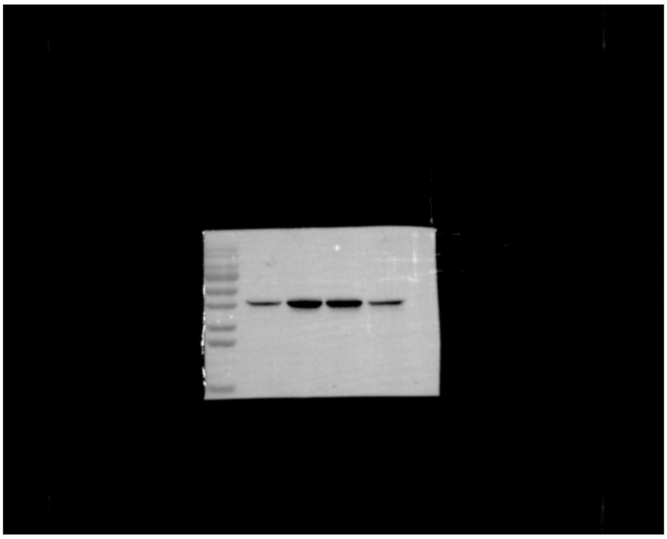

DRP1

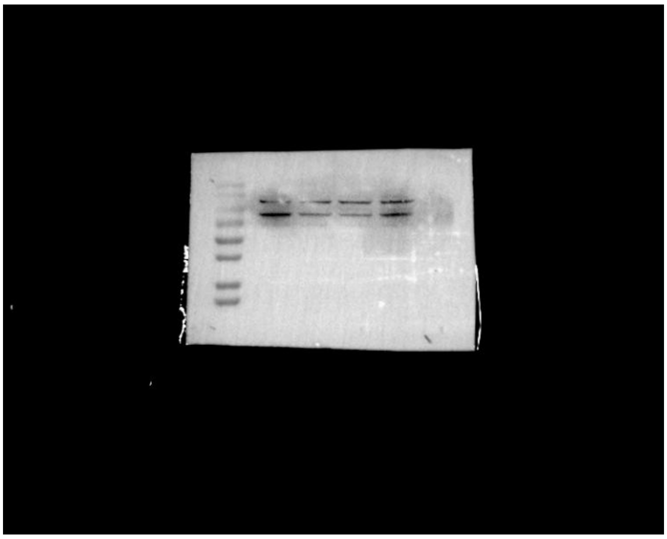

Figure 7A
AMPK

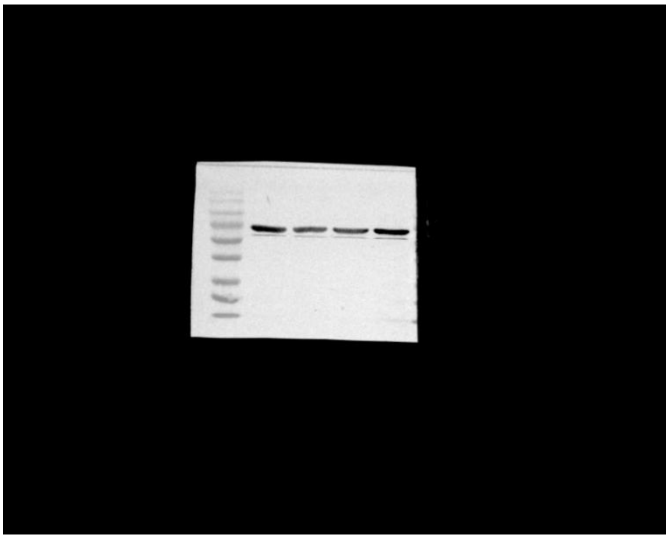

p-AMPK

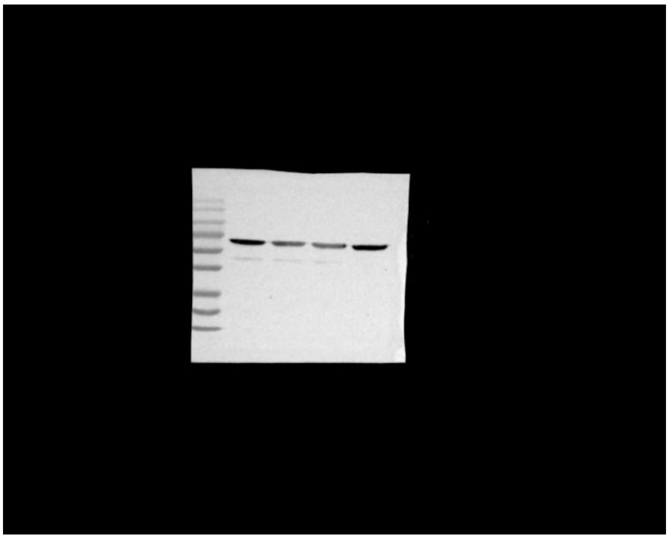

mTOR

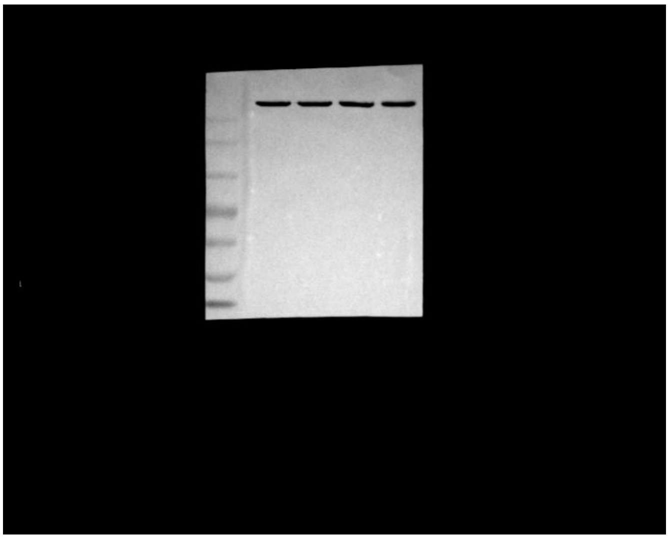

p-mTOR

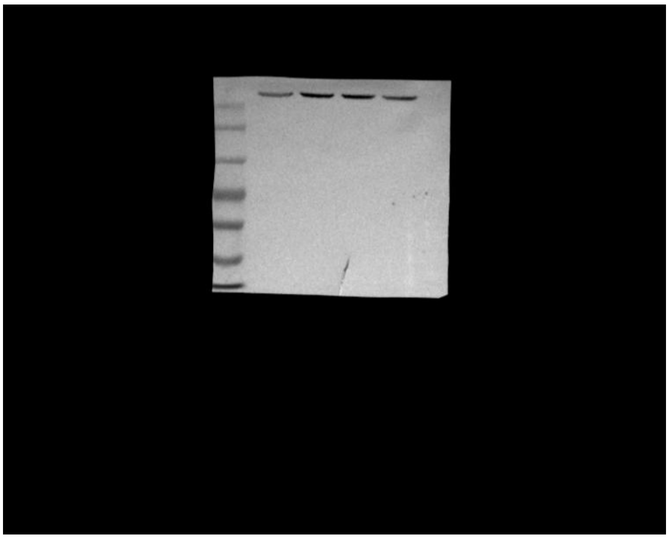

ULK1

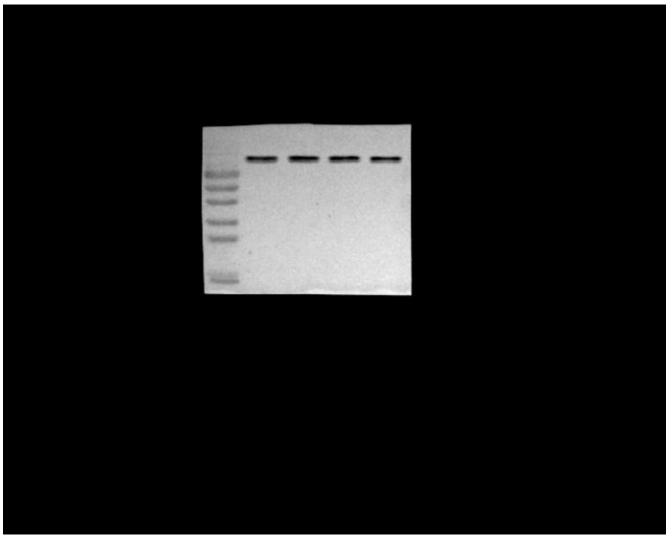

p-ULK1

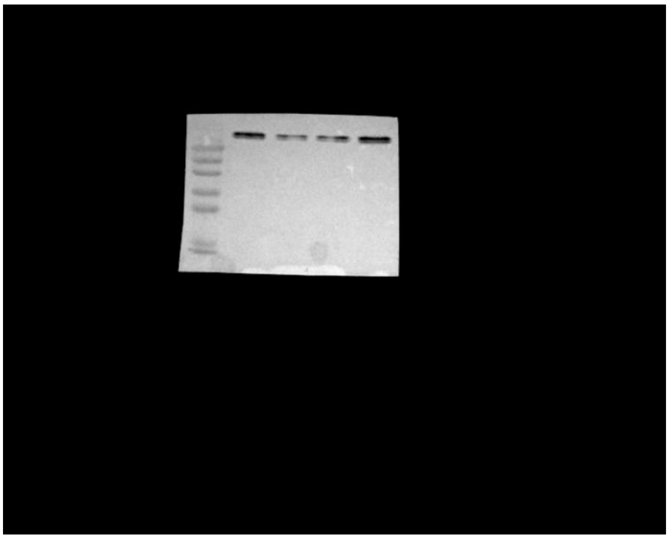

S6K

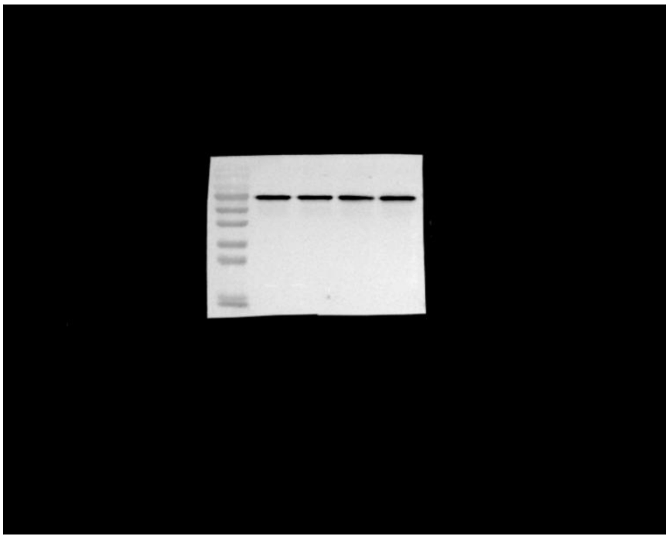

p-S6K

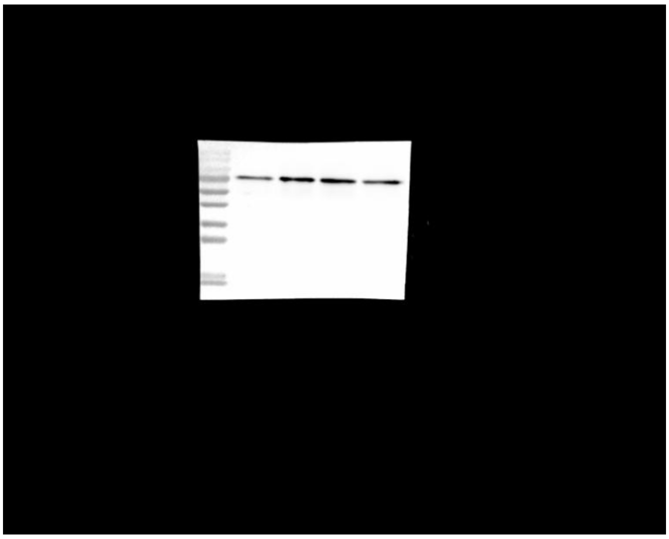

GAPDH

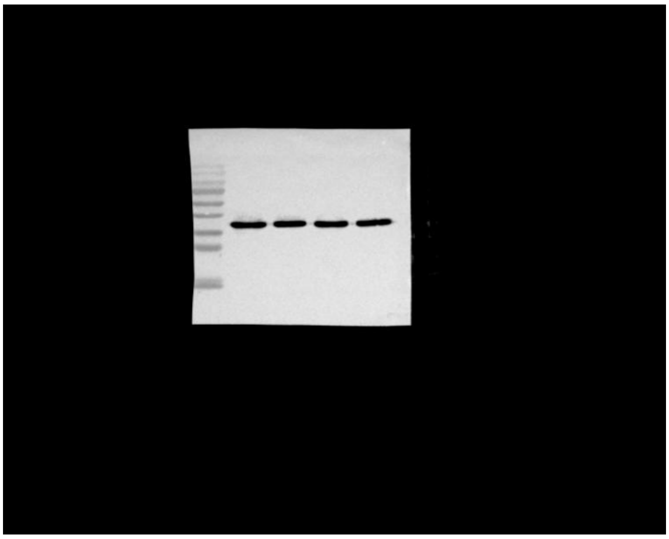

Figure 7E

SOGA1

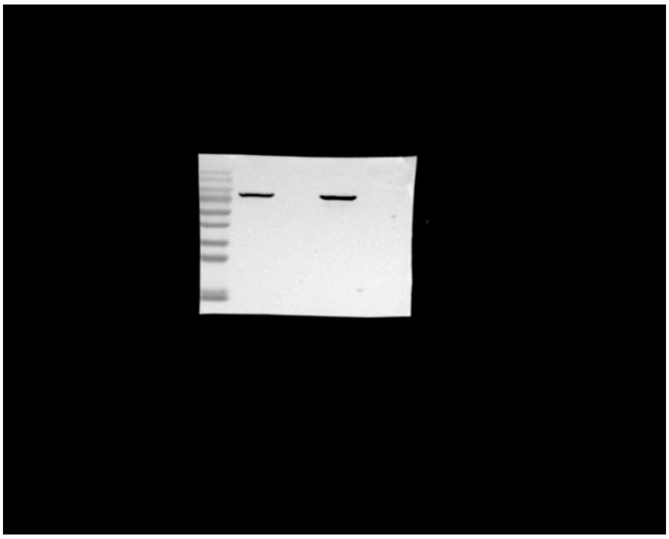

RNF41

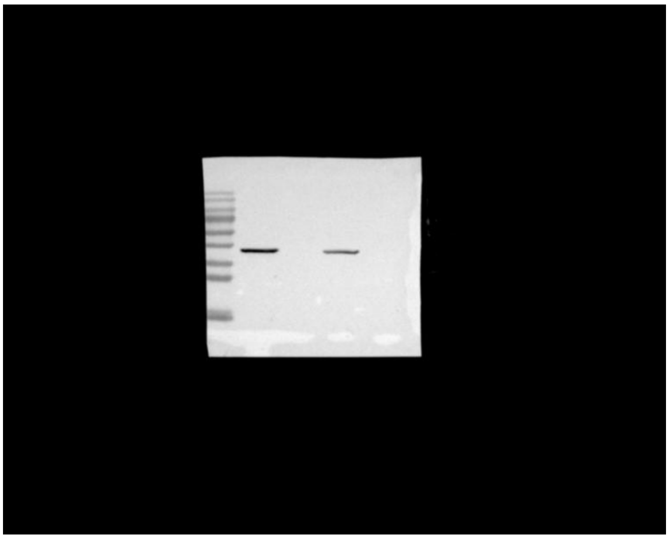

Figure 7F

RNF41

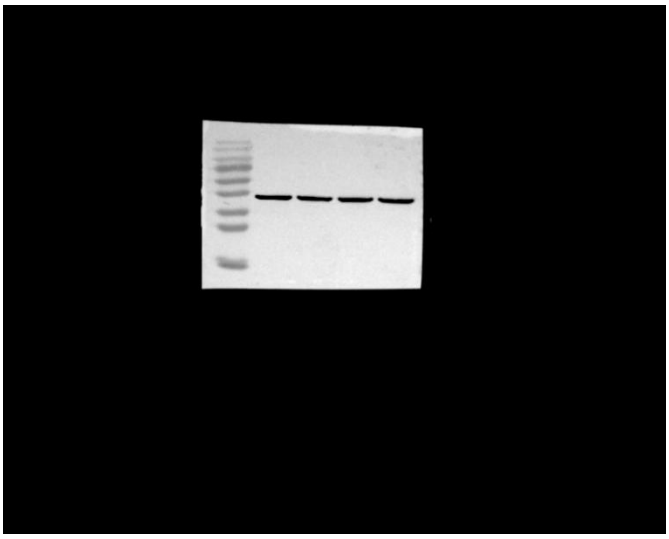

GAPDH

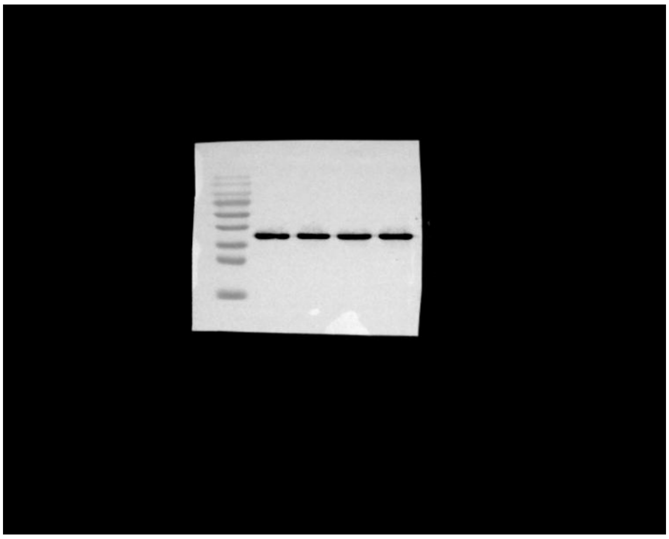

Figure 7H

Ub

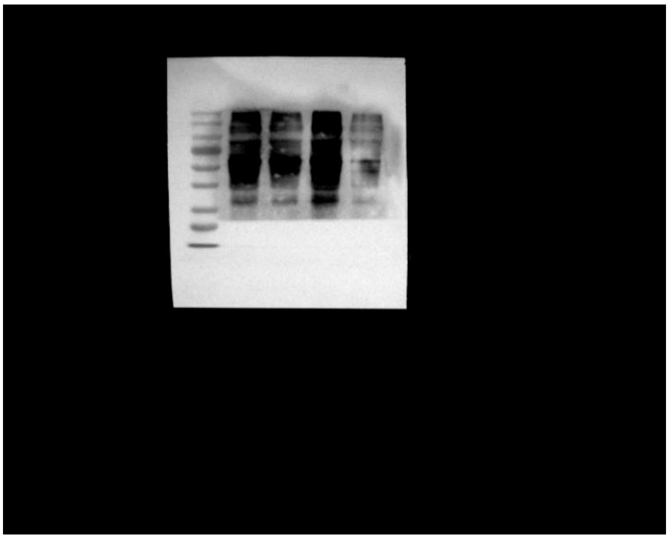

SOGA1

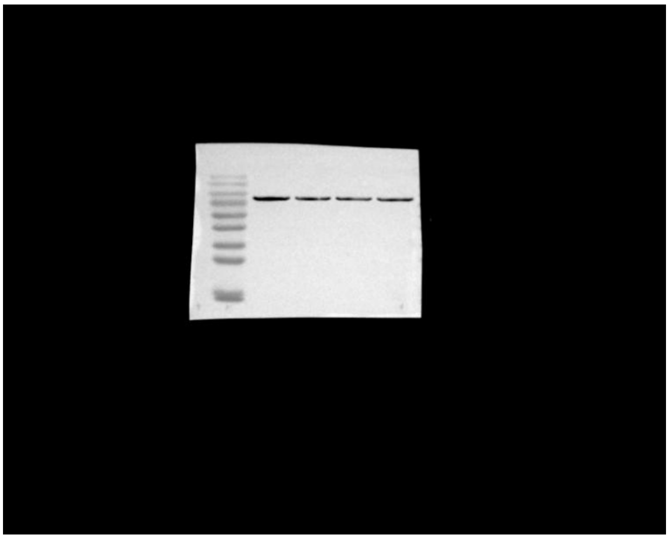

RNF41

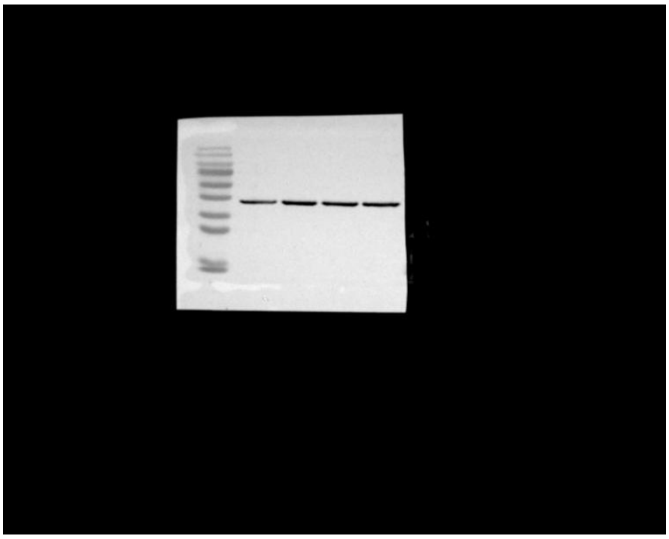

AMPK

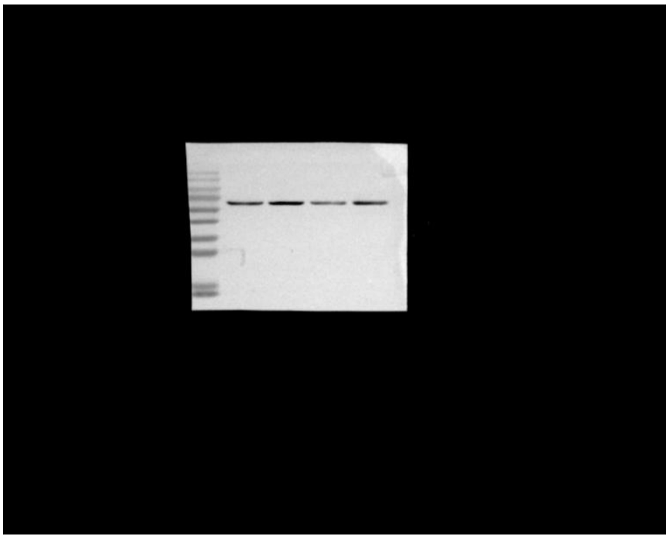

GAPDH

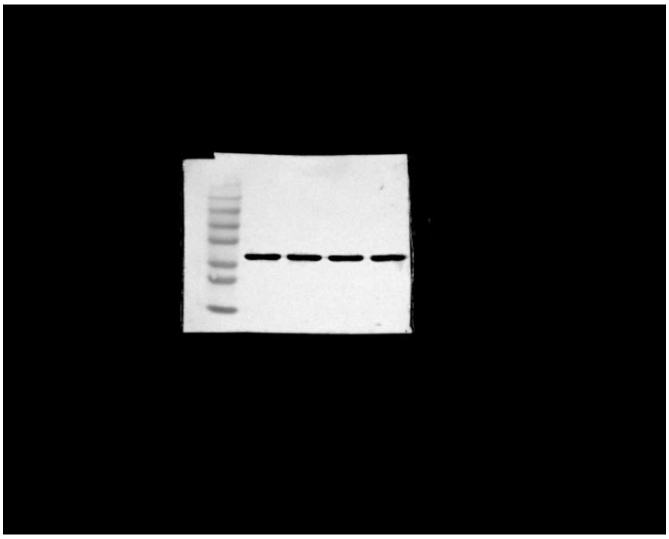

Figure 7I

AMPK

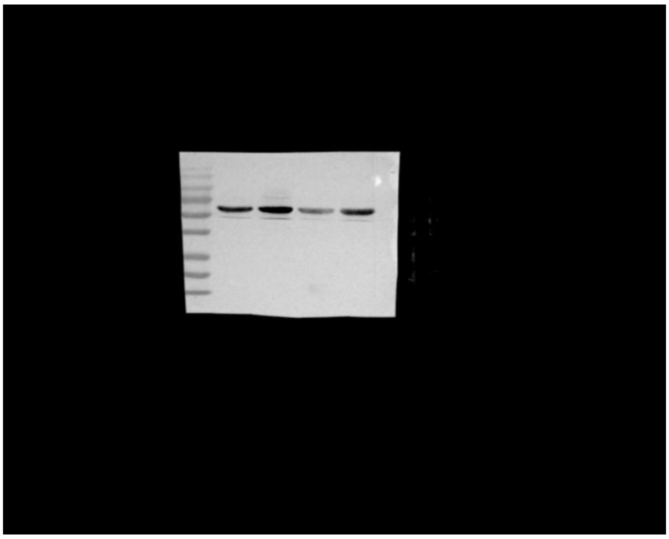

p-AMPK

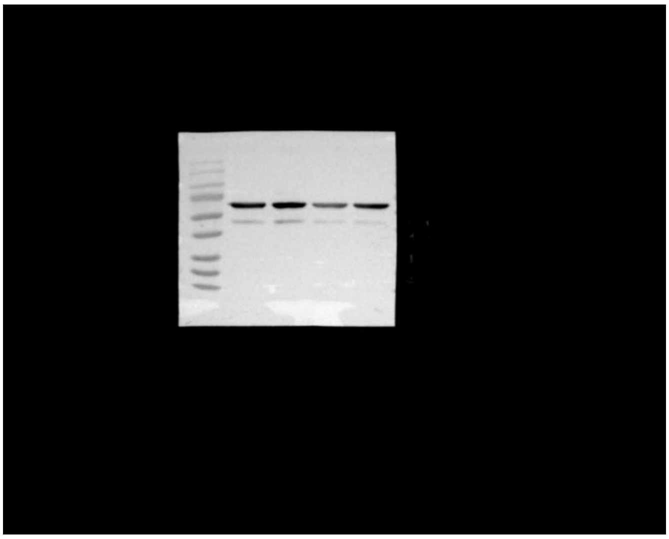

GAPDH

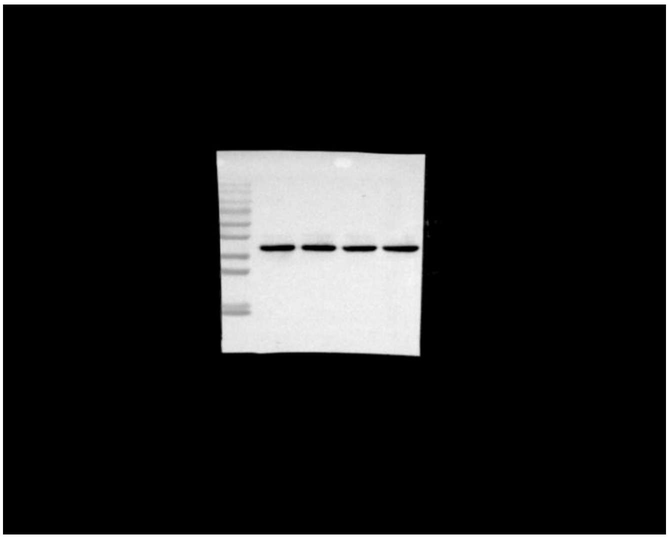

Figure 8E
SOGA1

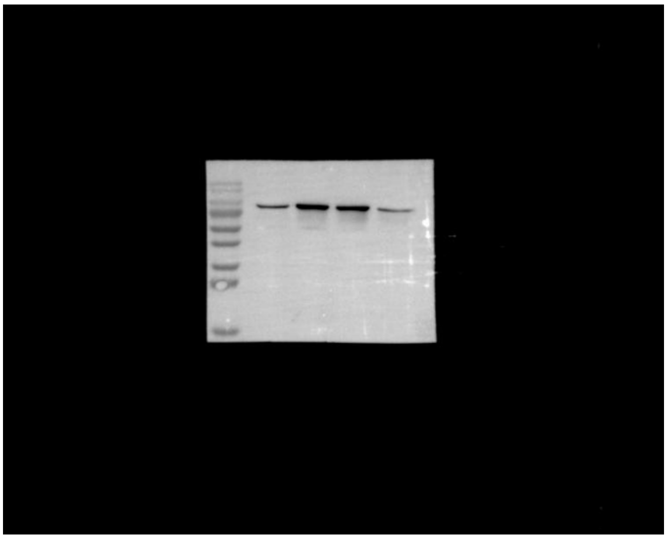

Figure 8F
P53

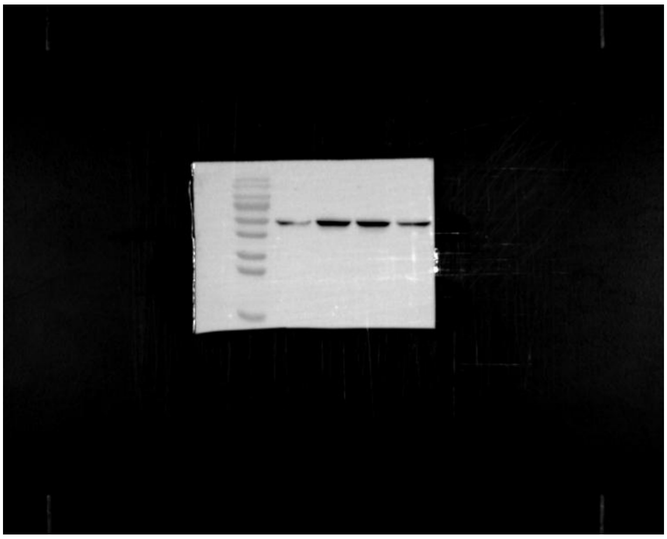

P21

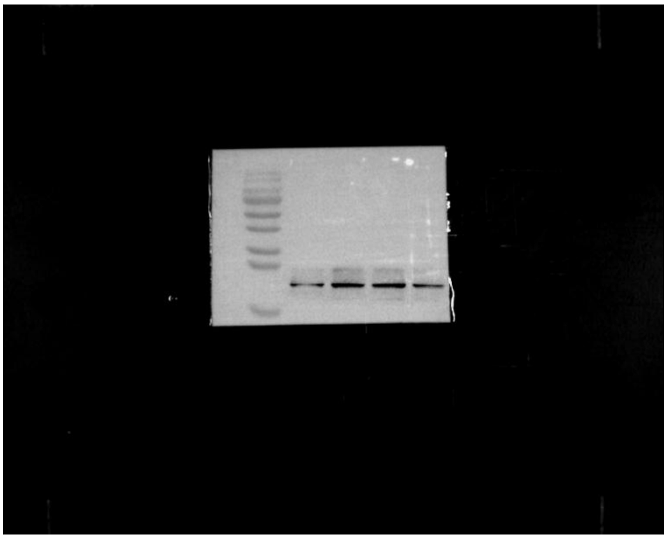

α-SMA

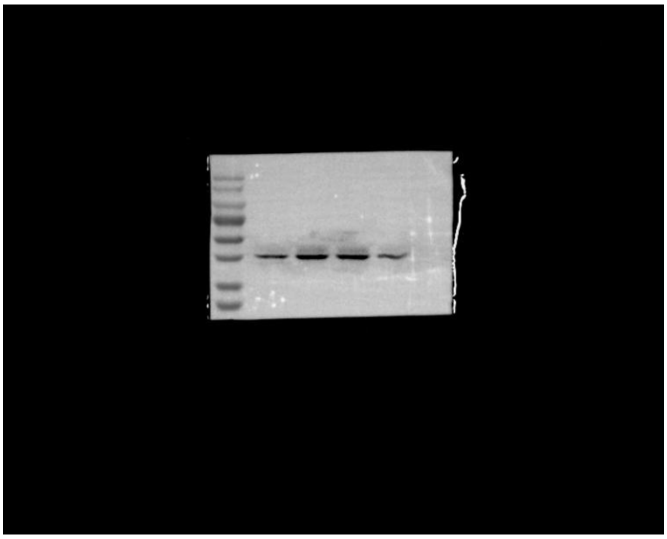

Figure 8G

AMPK

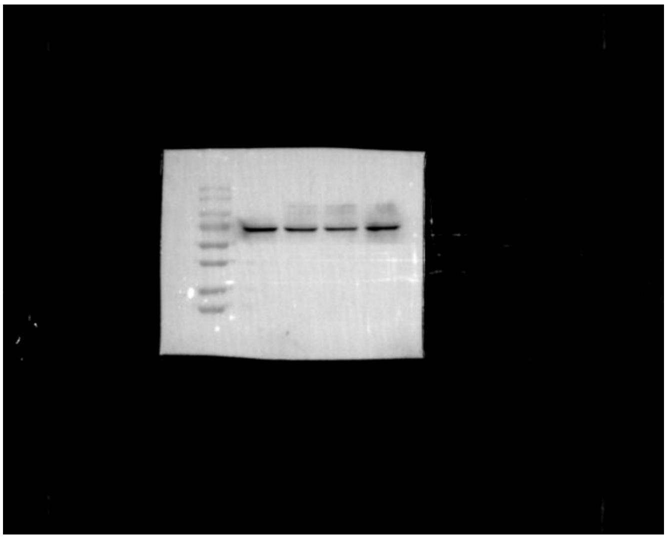

p-AMPK

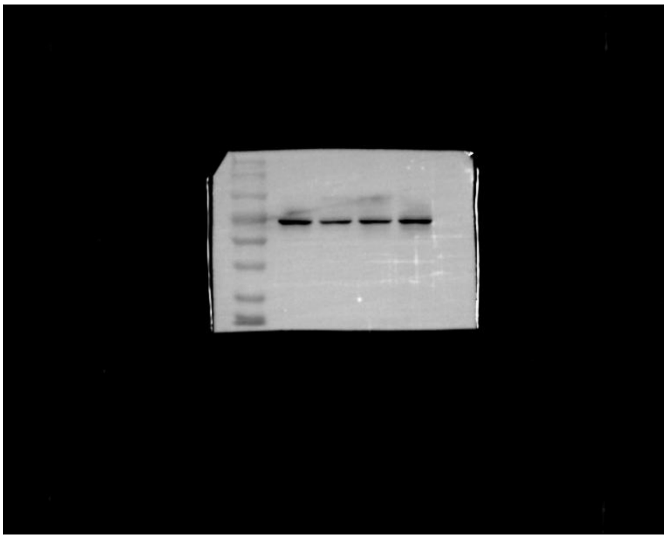

p-mTOR

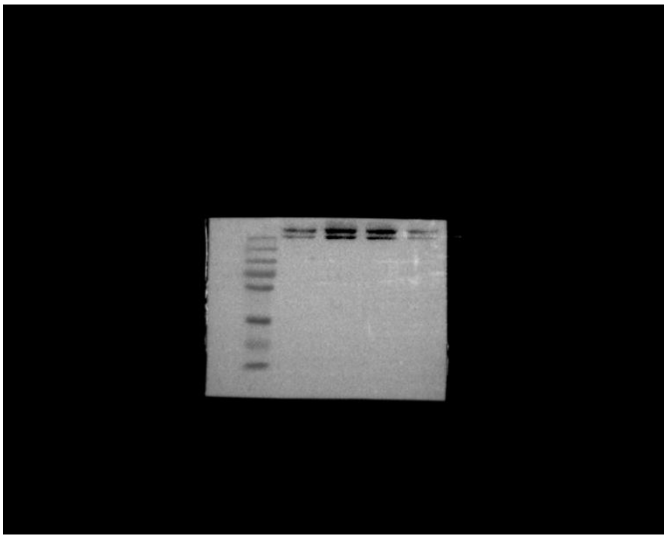

Figure 8I

LC3 II/I

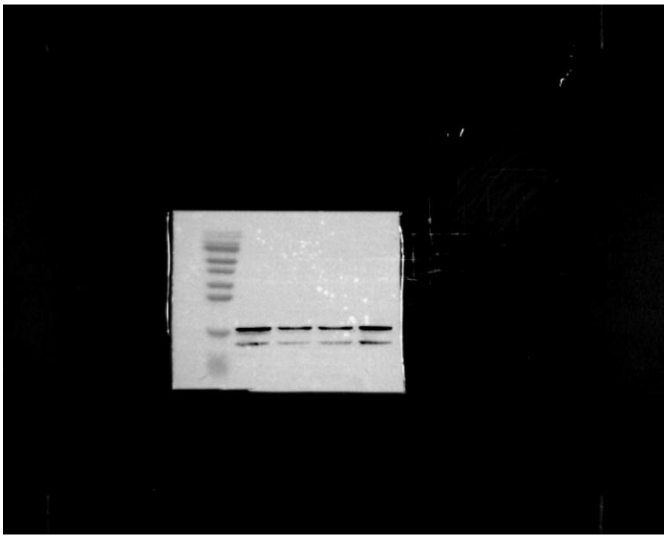

PINK1

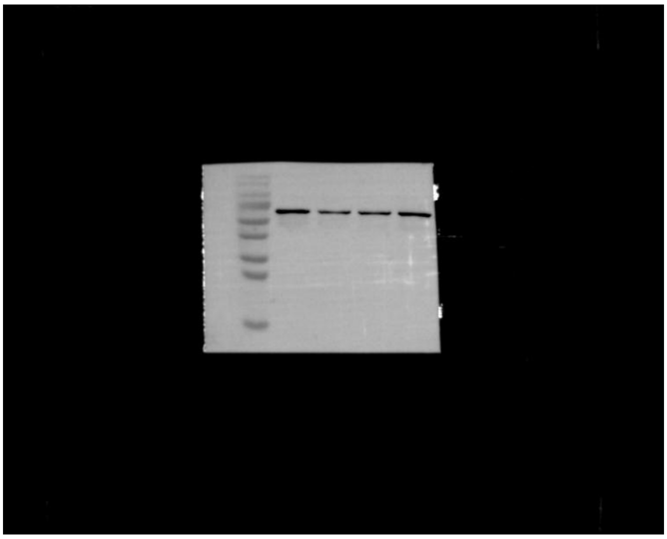

S-Figure 1A

TNF-α

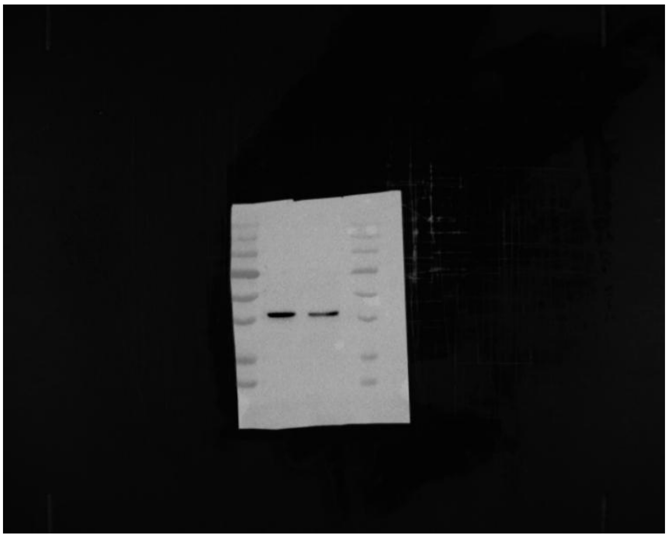

IL-1β

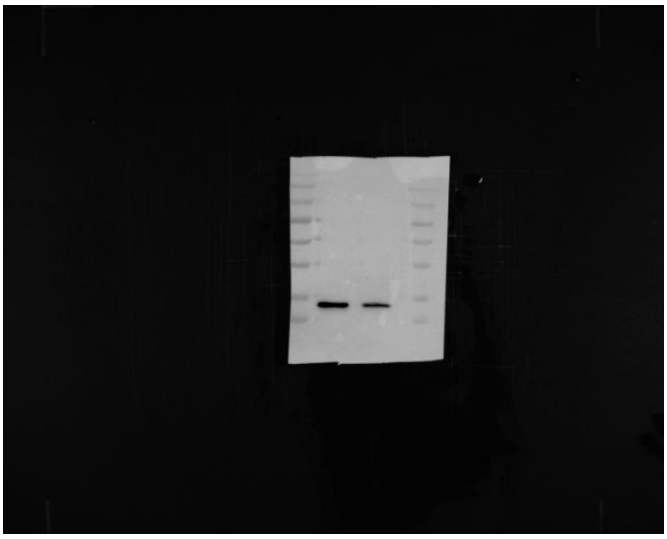

IL-6

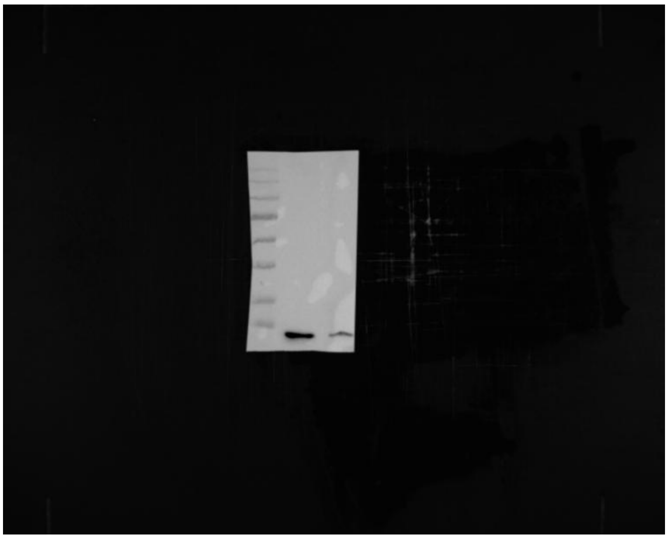

PDGF

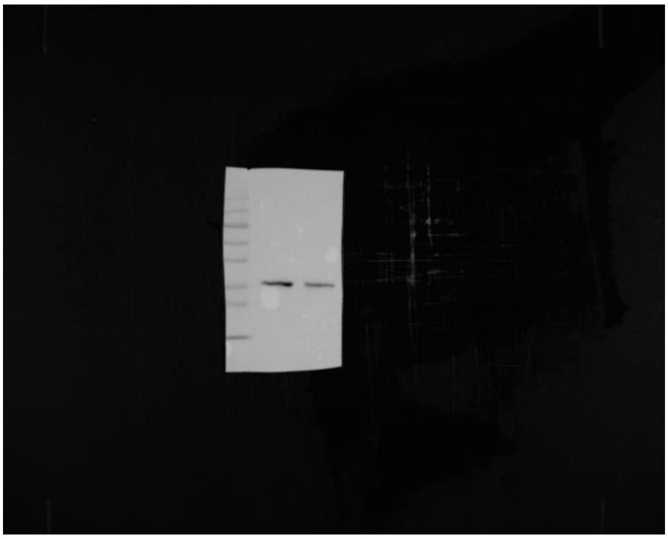

TGF-β

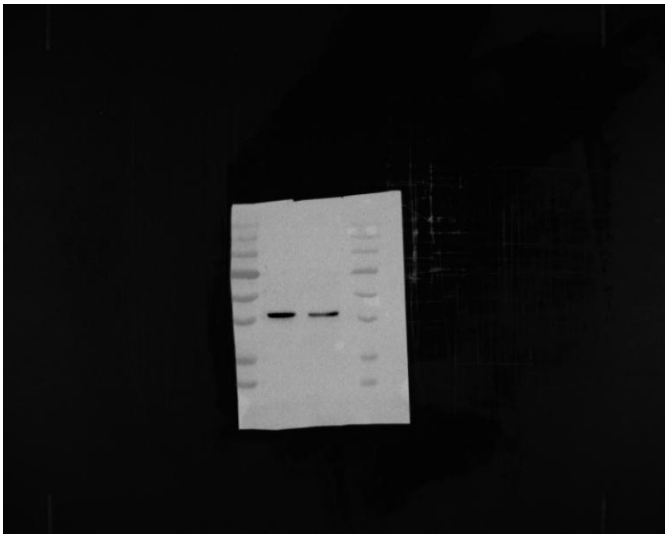

GAPDH

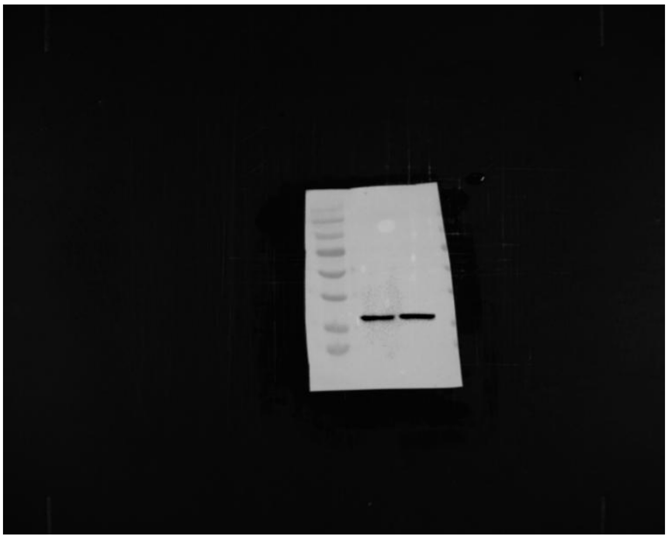

S-Figure 1B

Collagen I

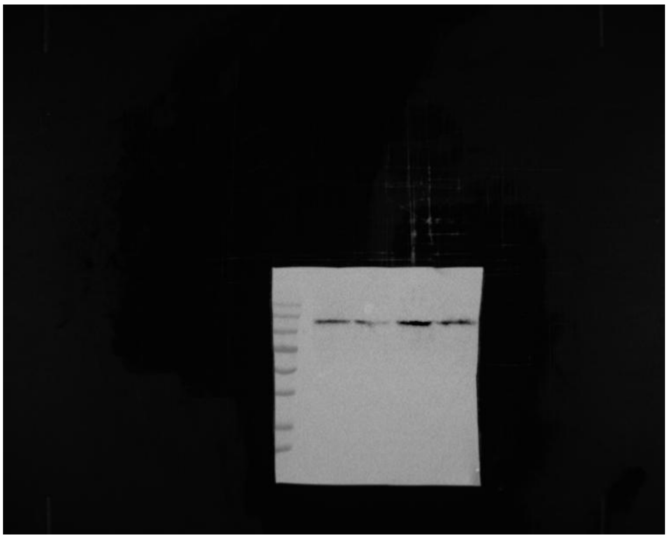

Collagen III

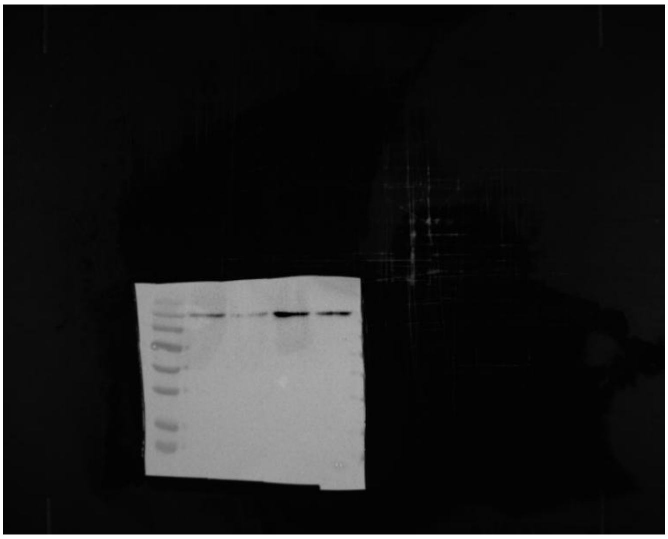

α-SMA

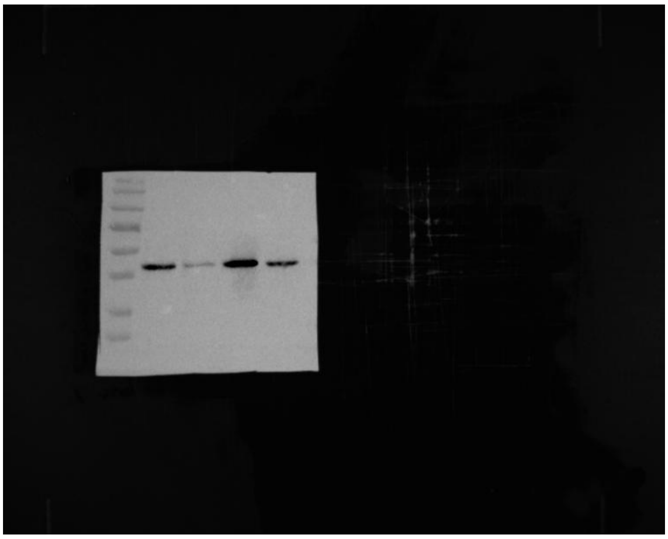

GAPDH

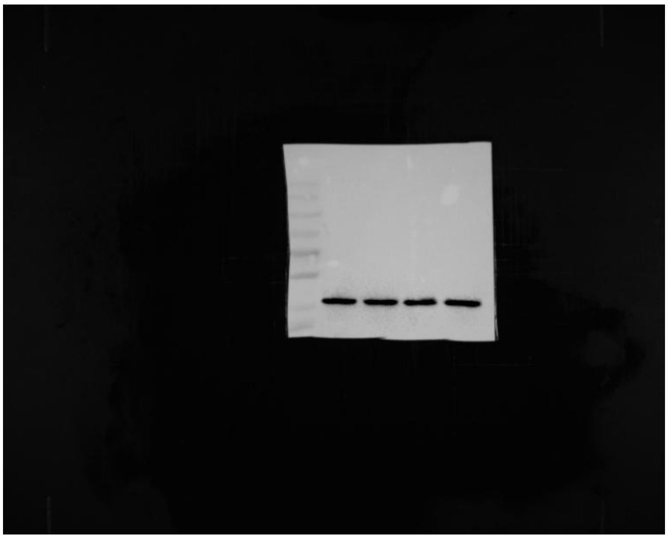

S-Figure 2A

LC3 II/I

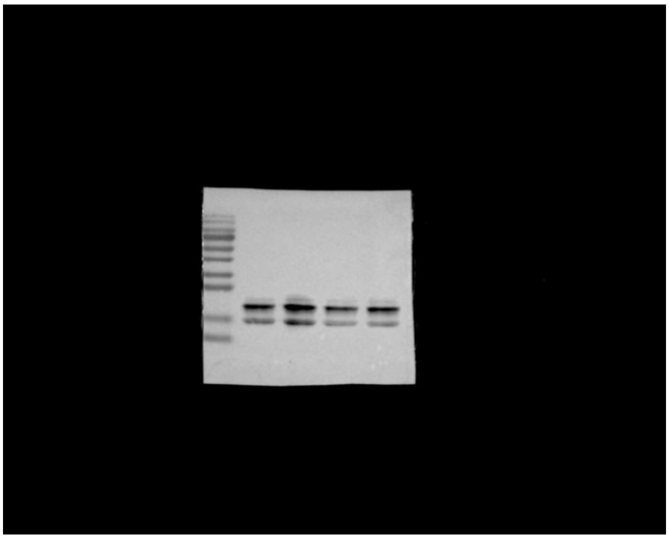

p62

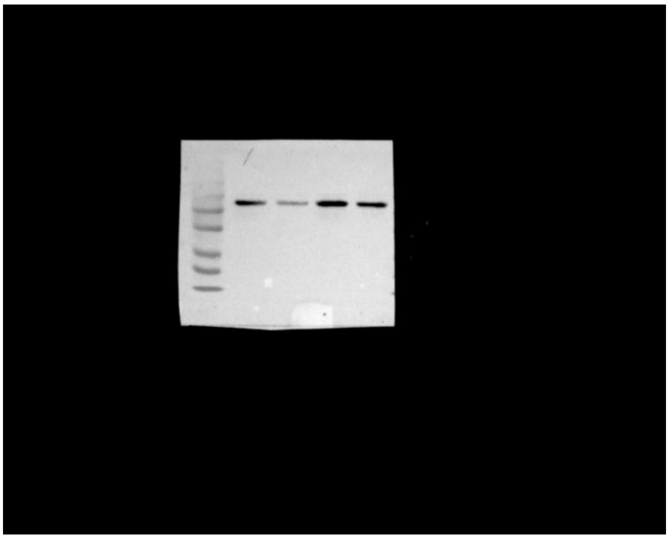

GAPDH

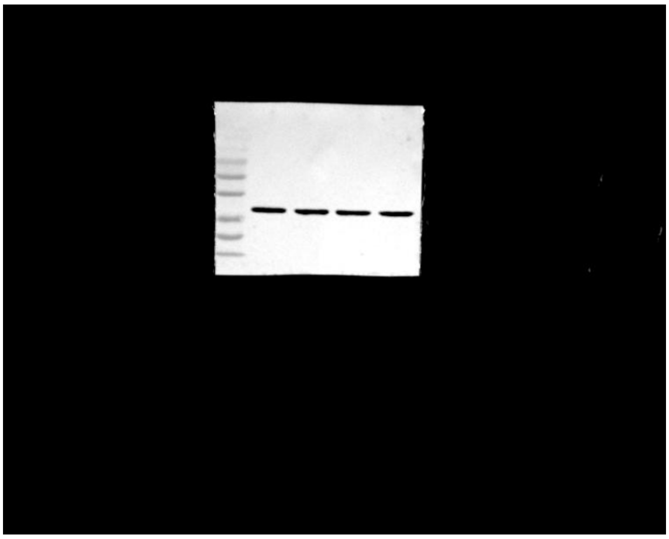

S-Figure 2B

p53

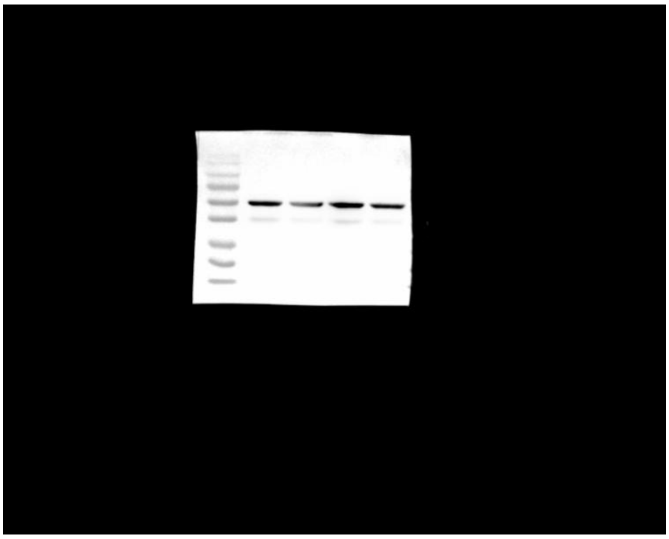

p21

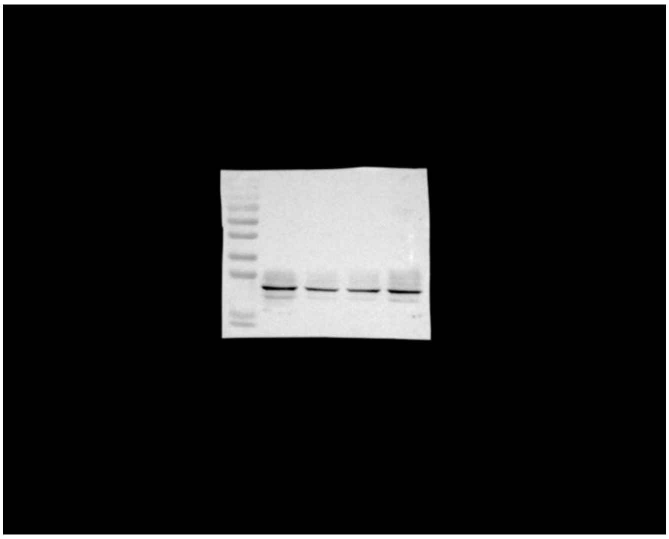

γ-H2AX

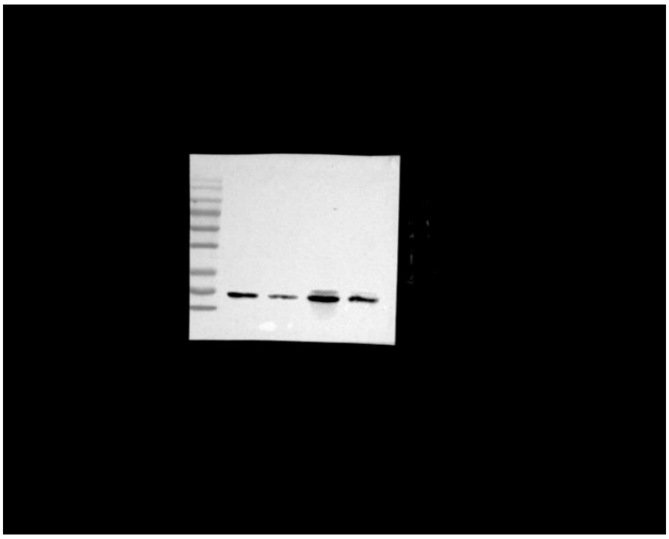

GAPDH

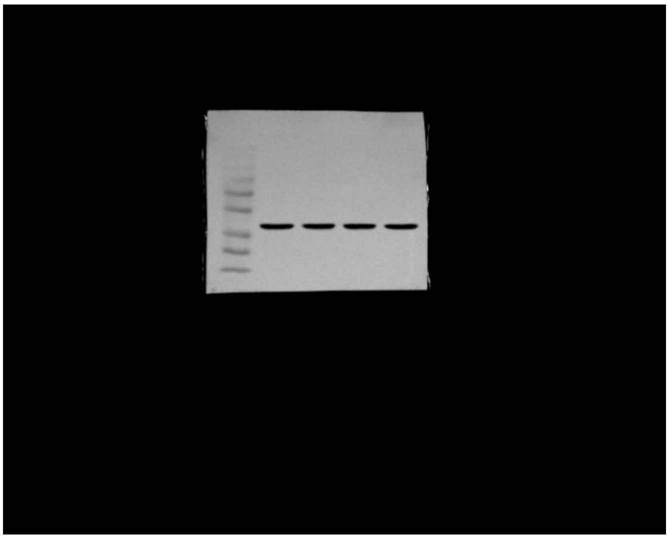

S-Figure 2E

Collagen I

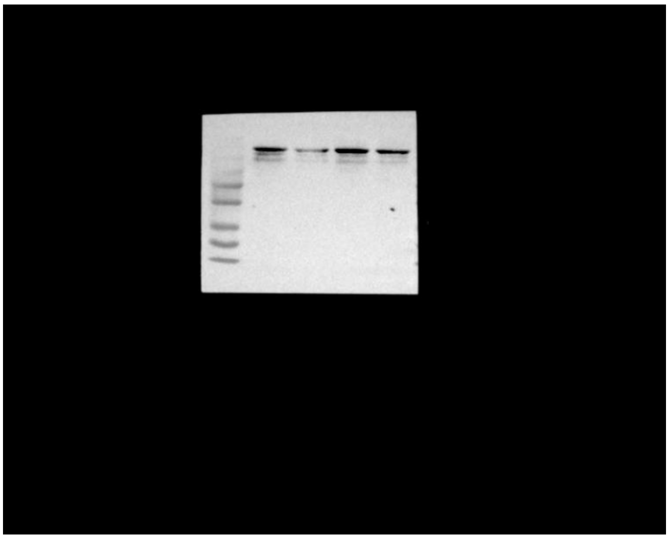

Collagen III

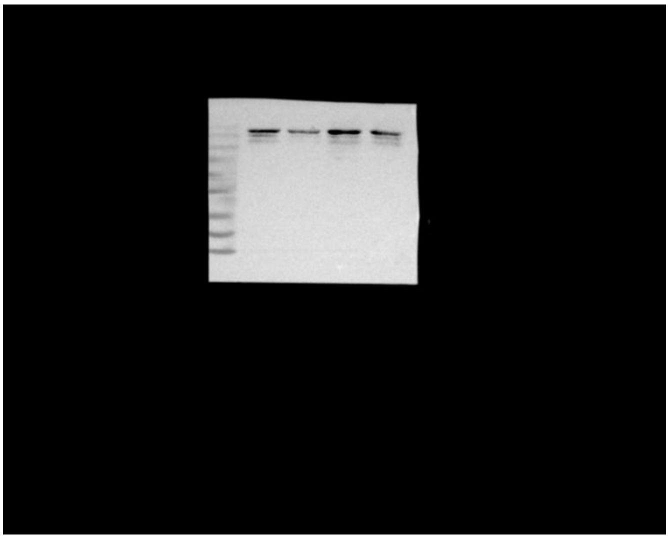

α-SMA

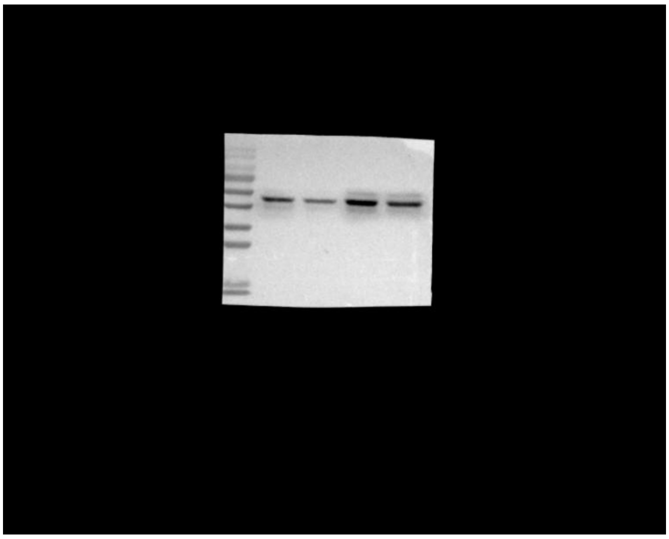

GAPDH

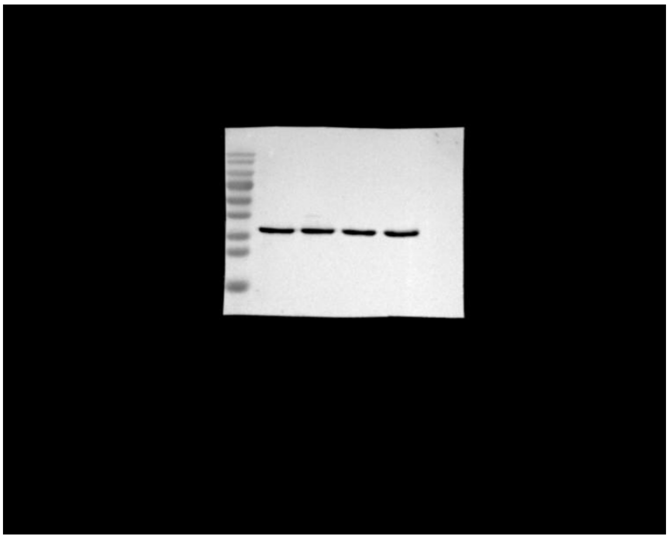

S-Figure 3A

AMPK

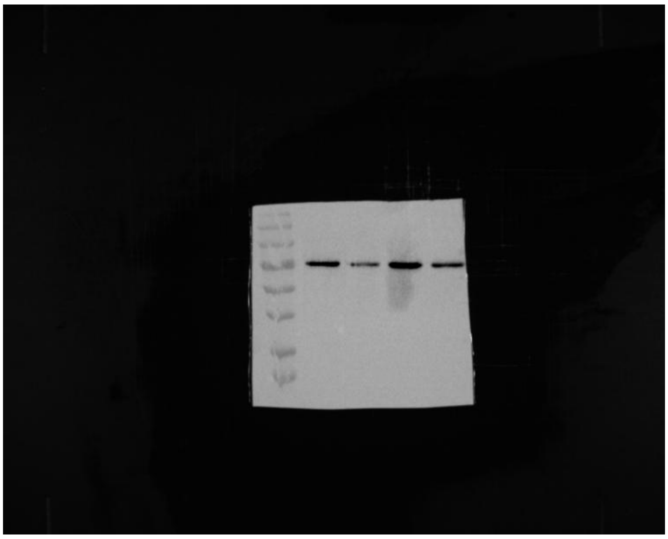

p-AMPK

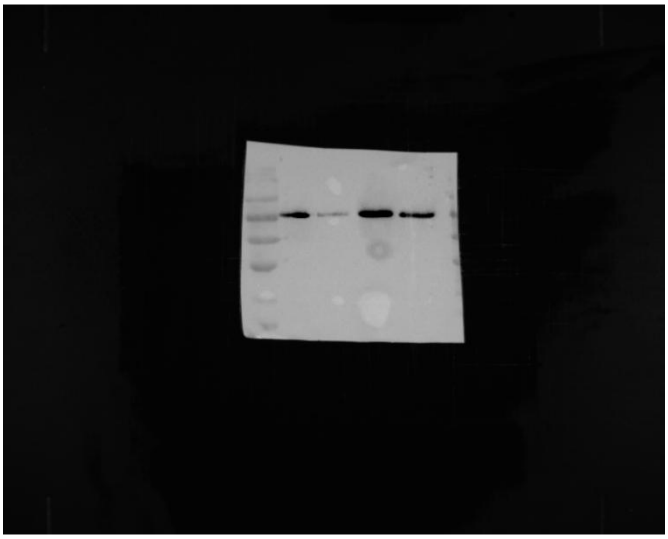

mTOR

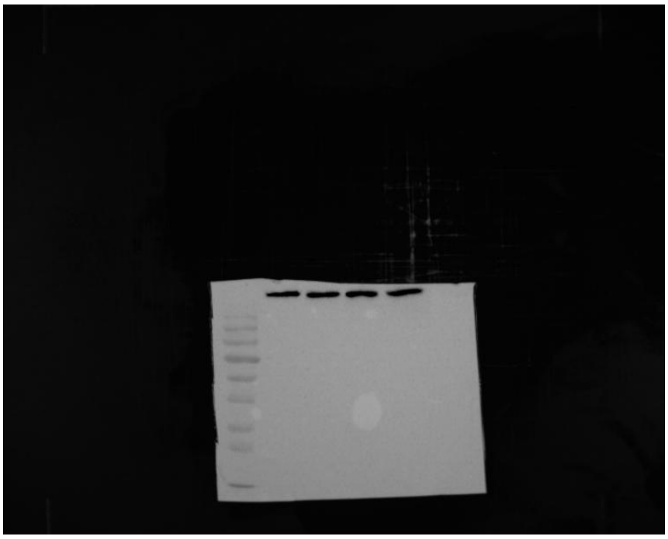

p-mTOR

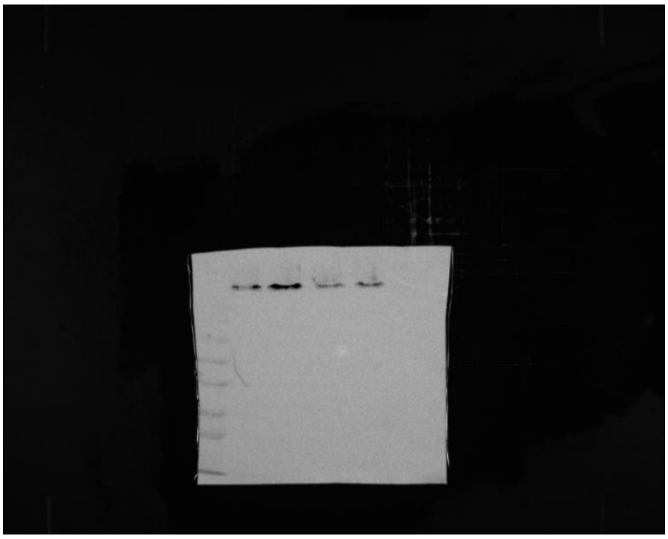

GAPDH

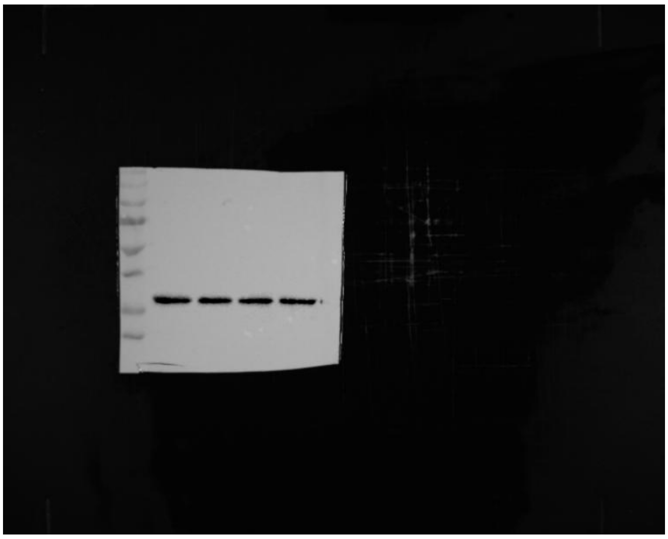

S-Figure 3B

LC3 II/I

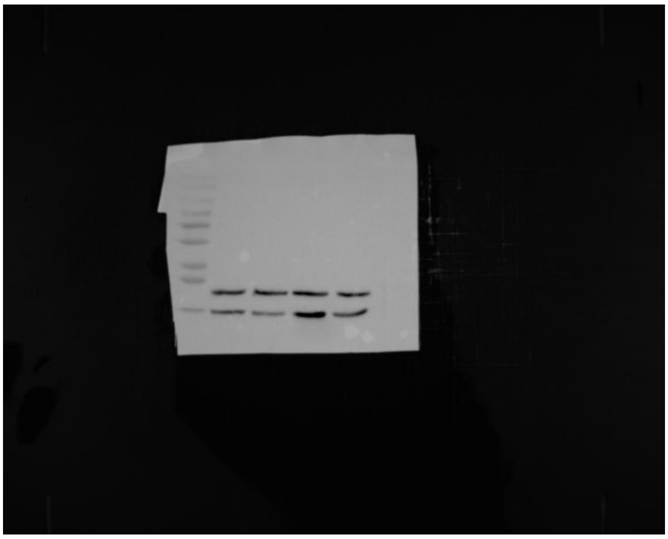

p62

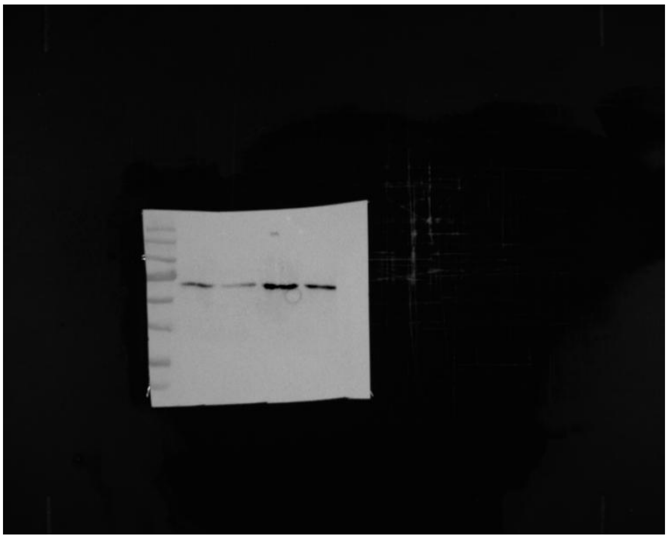

GAPDH

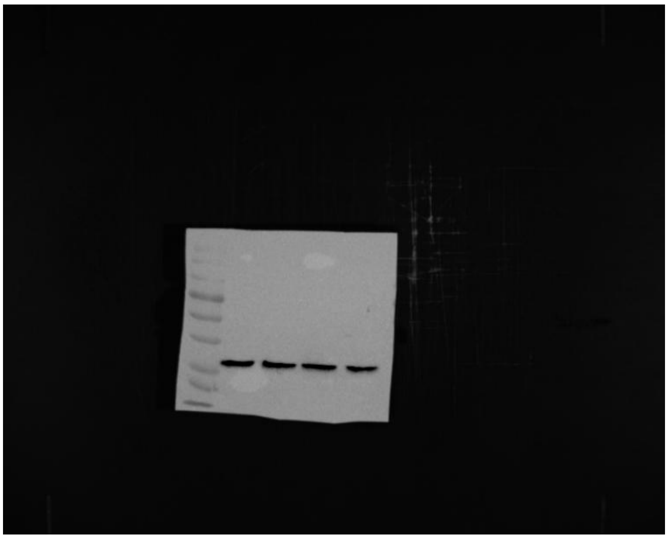

S-Figure 3C

p53

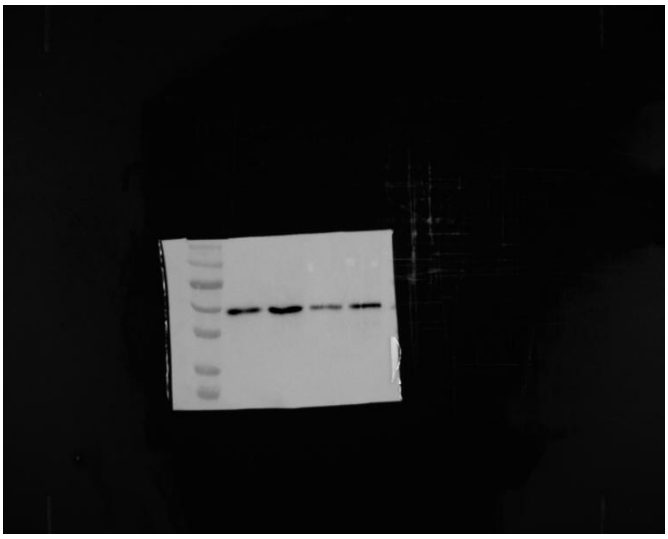

p21

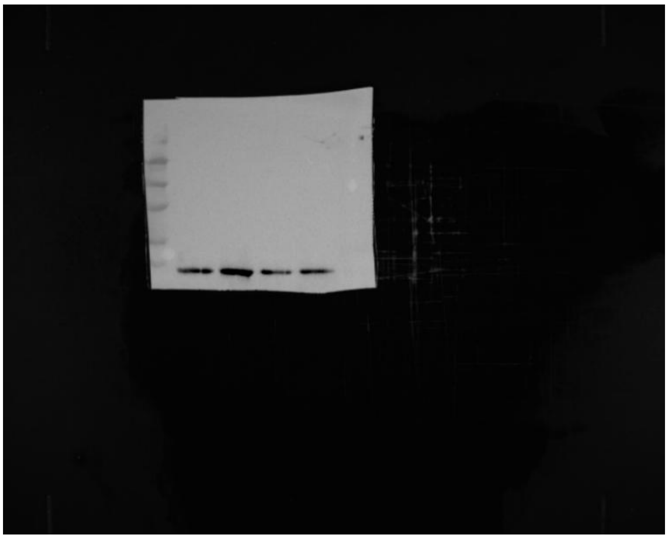

γ-H2AX

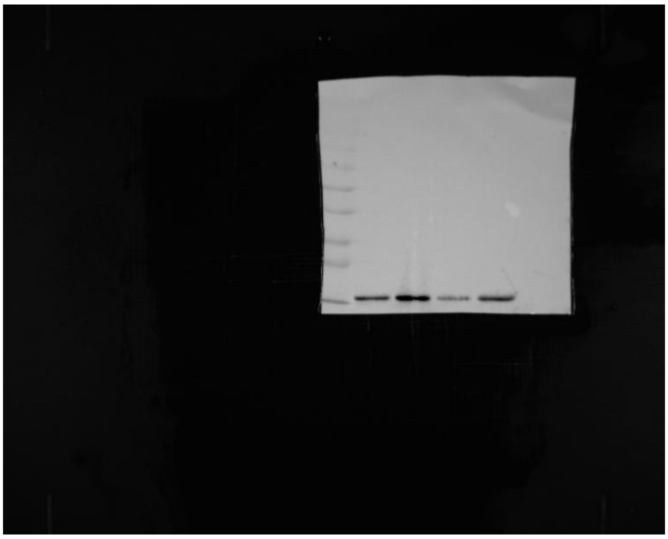

GAPDH

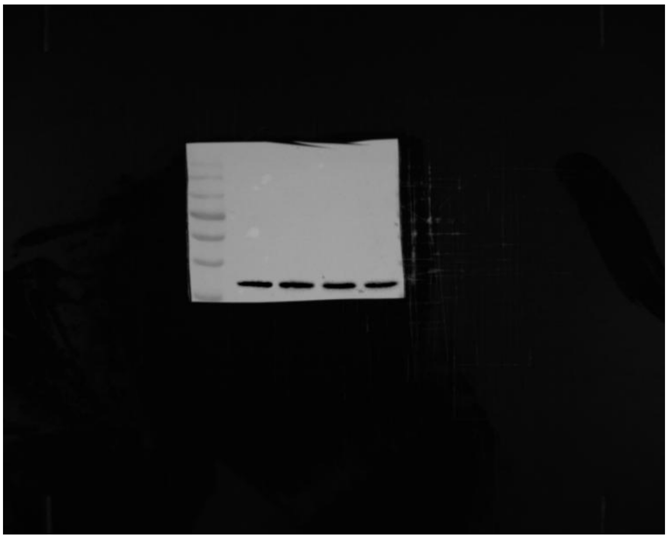

S-Figure 3E

Collagen I

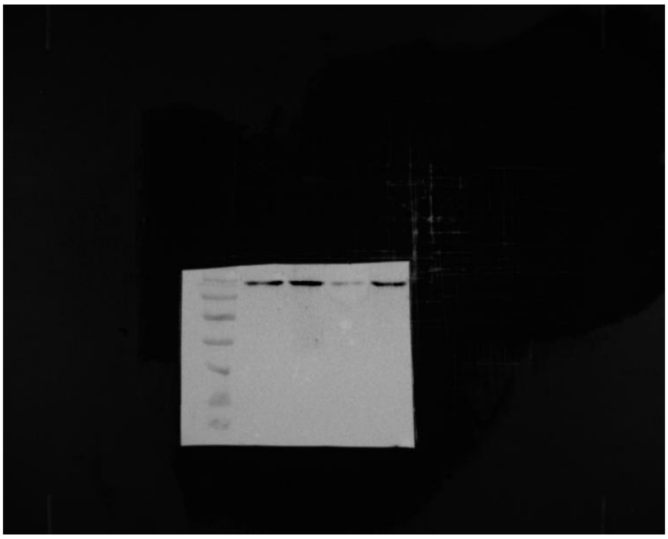

Collagen III

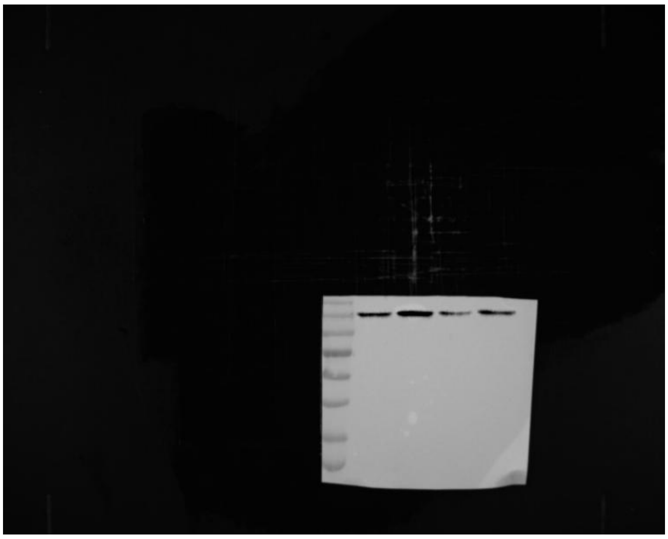

α-SMA

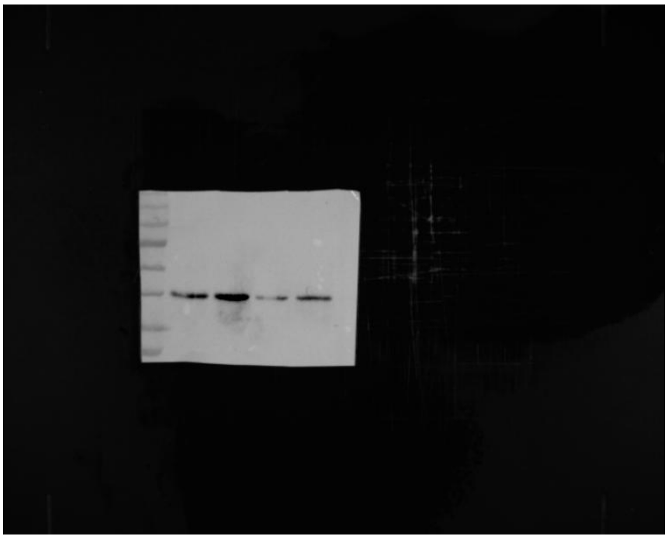

GAPDH

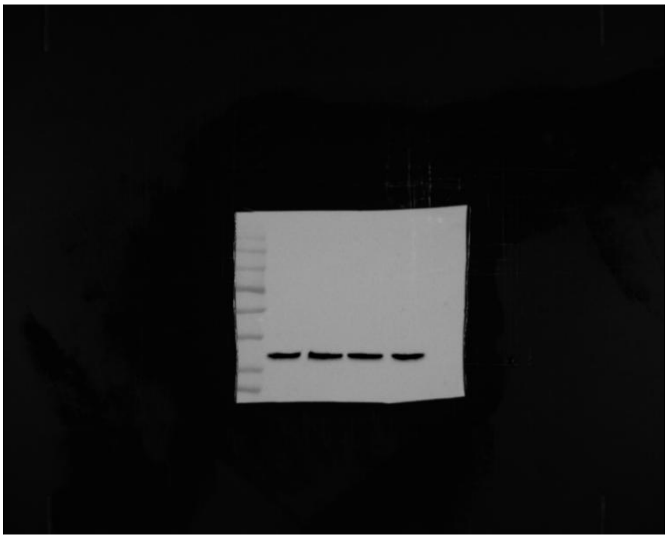

S-Figure 4A

AMPK

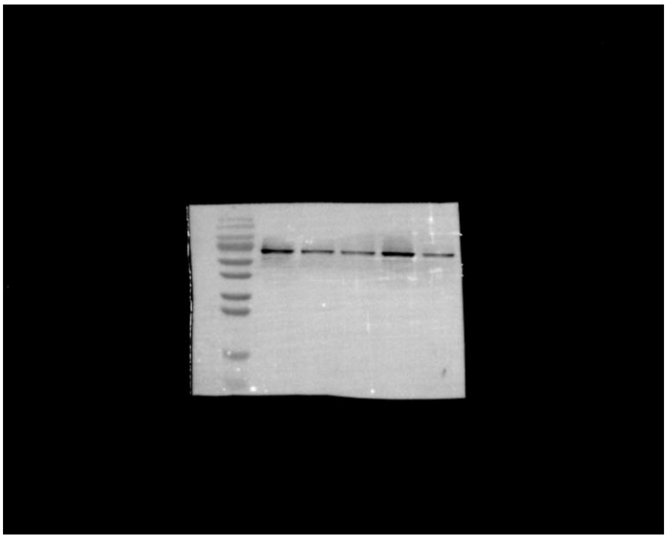

p-AMPK

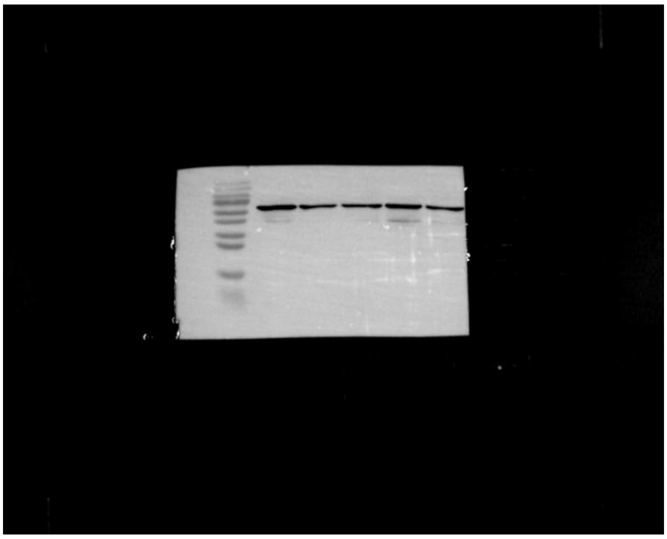

p-mTOR

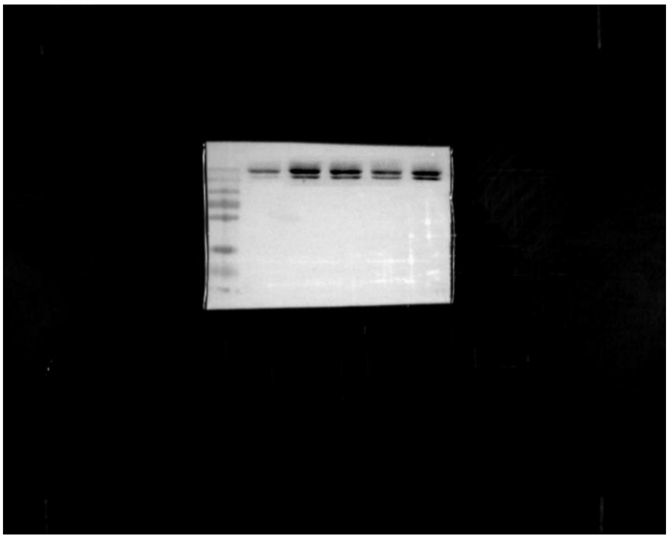

S-Figure 4B

LC3 II/I

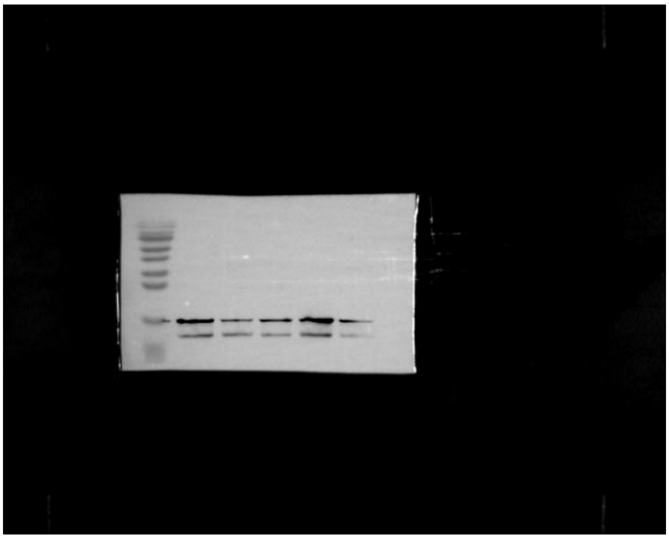

p62

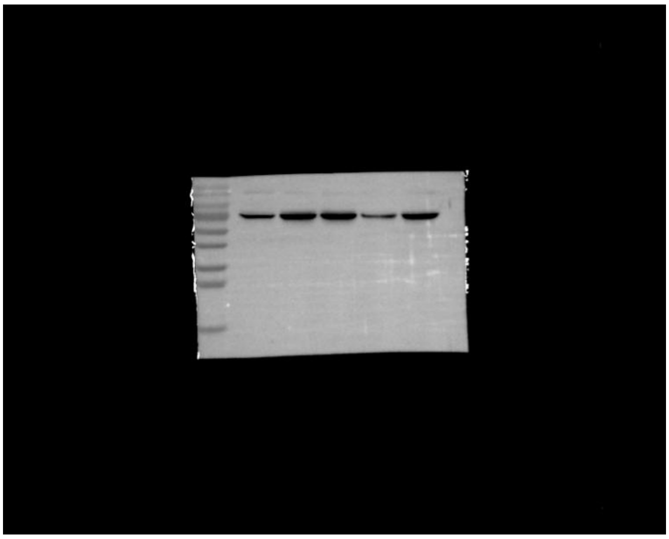

PINK1

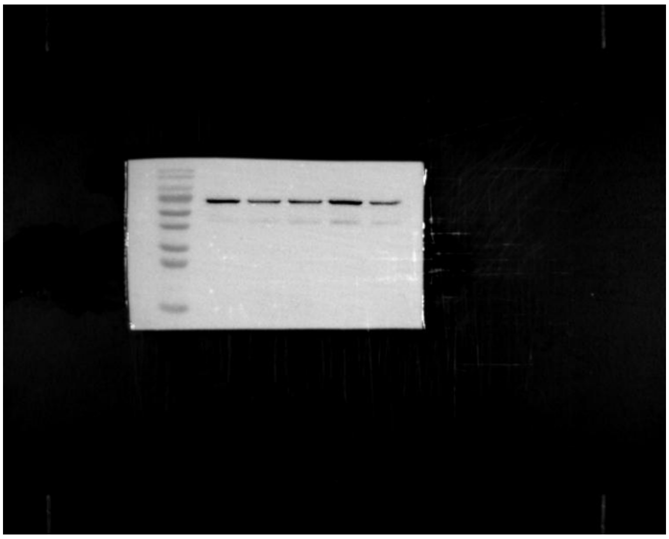

S-Figure 6A

SOGA1

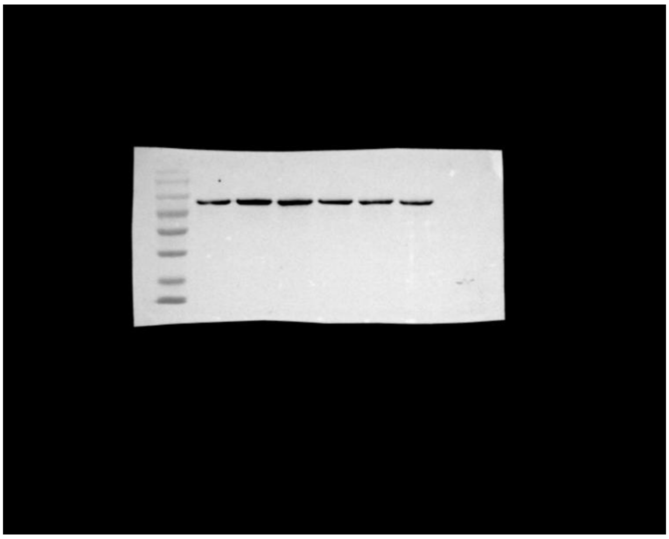

AMPK

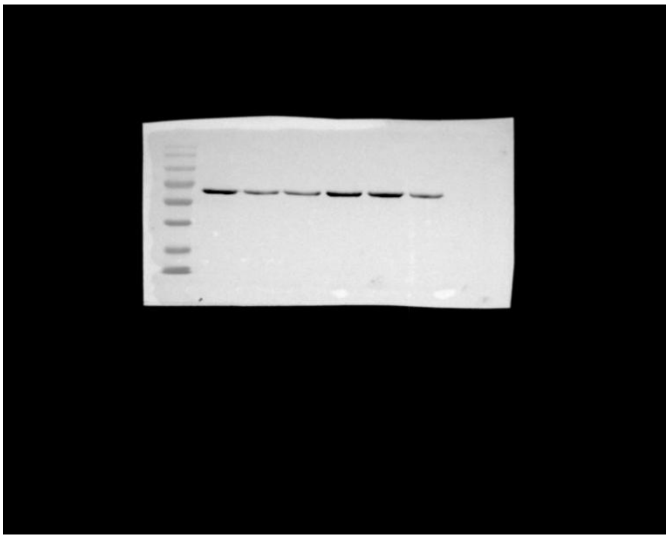

GAPDH

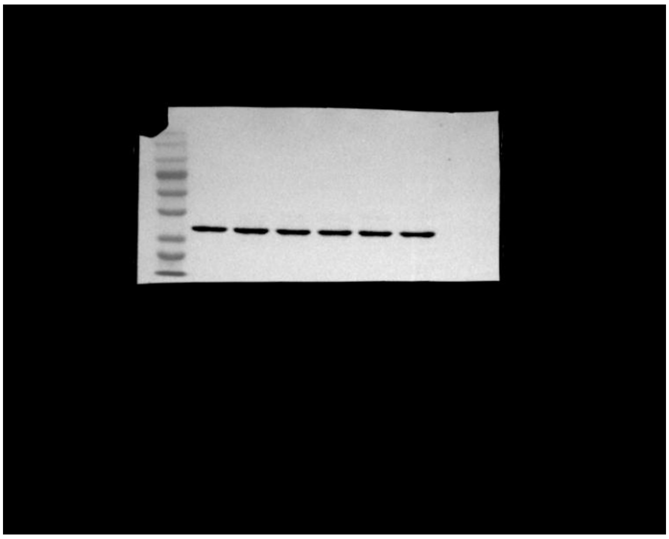

S-Figure 6B

p-AMPK

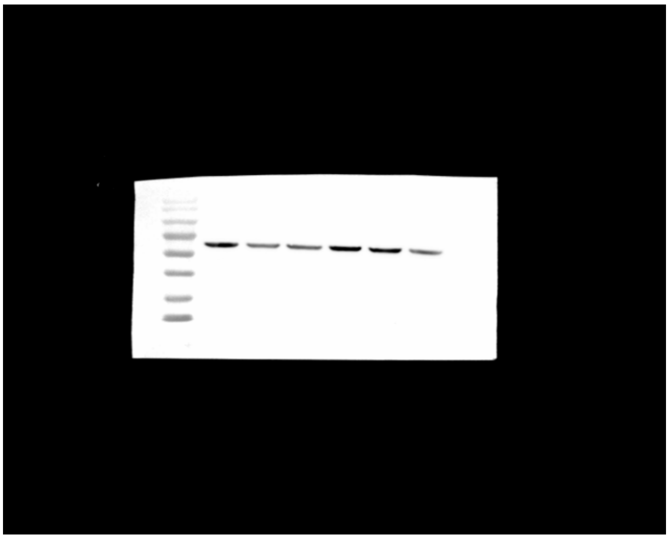

p-mROR

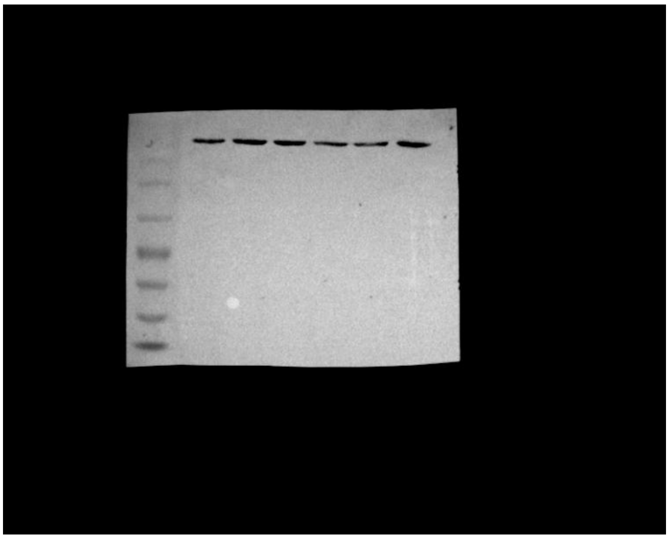

GAPDH

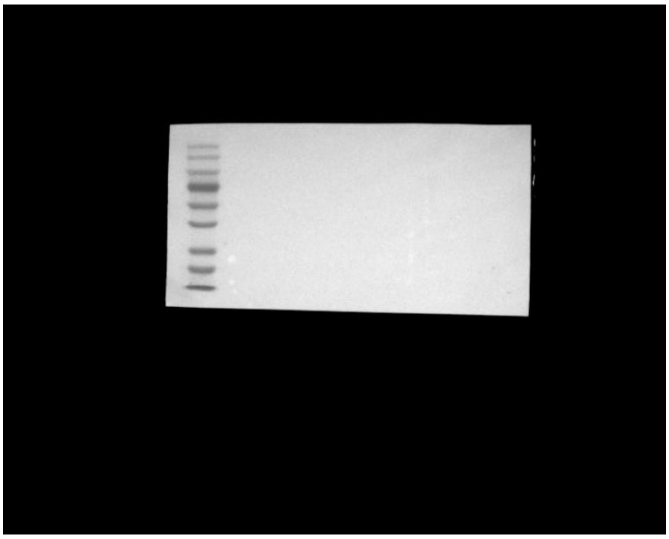

LC3 II/I

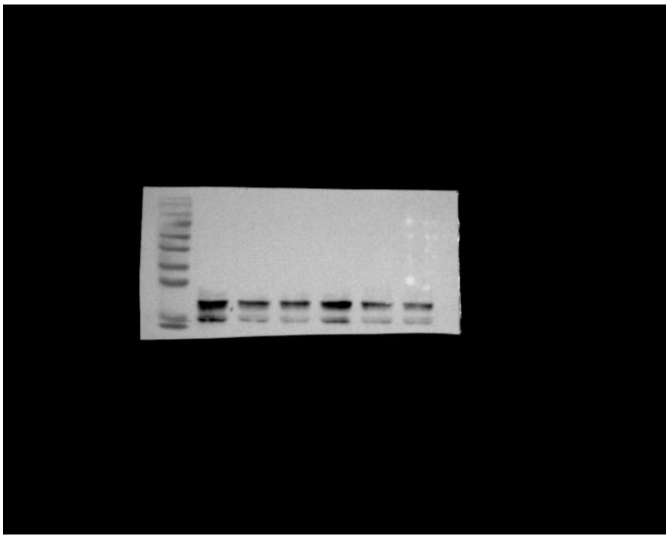

GAPDH

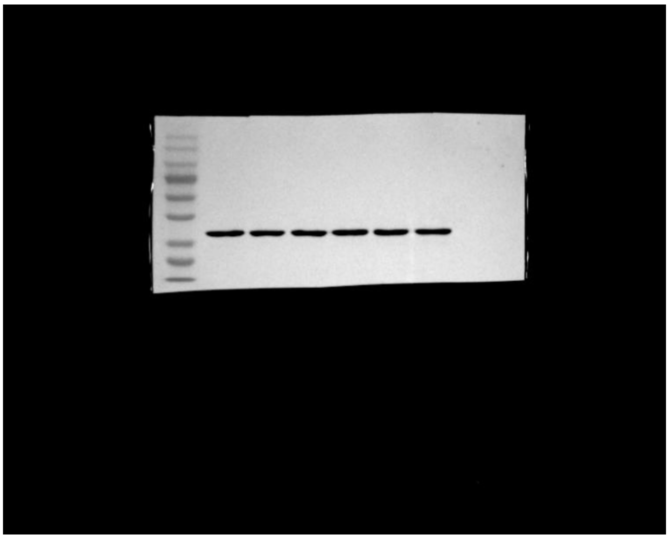

S-Figure 6F

p53

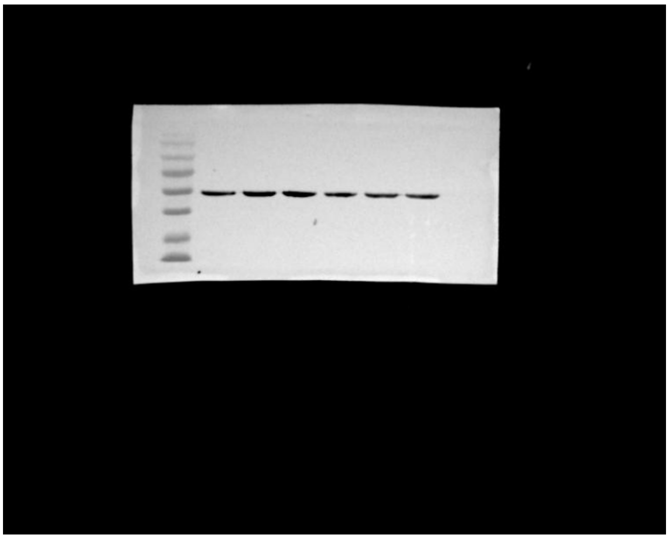

p21

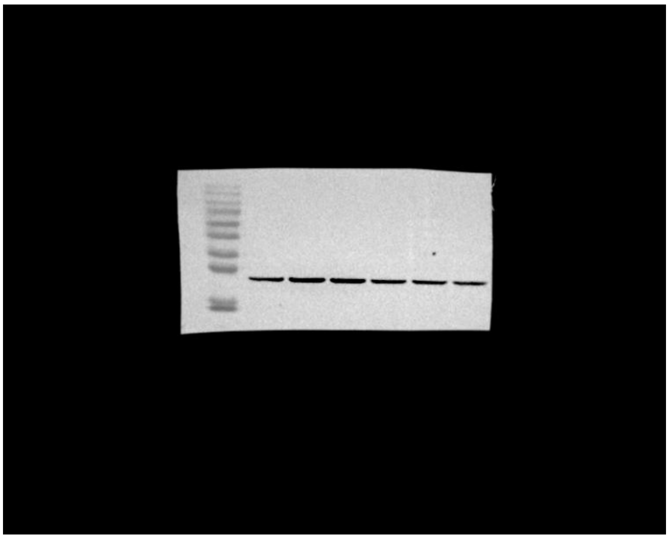

GAPDH

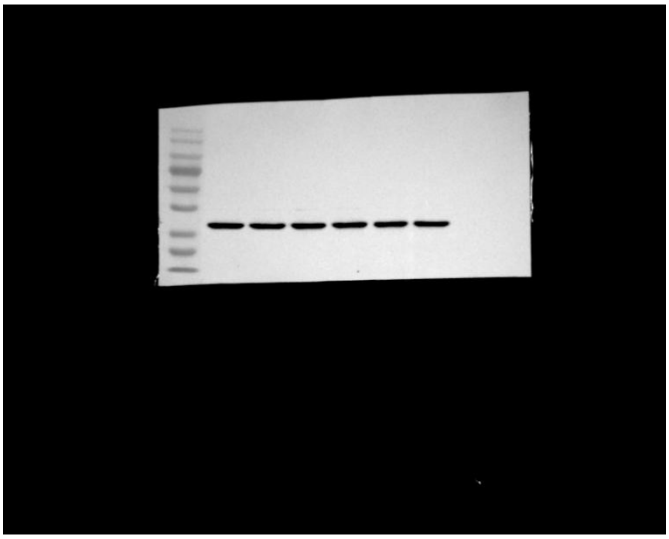

α-SMA

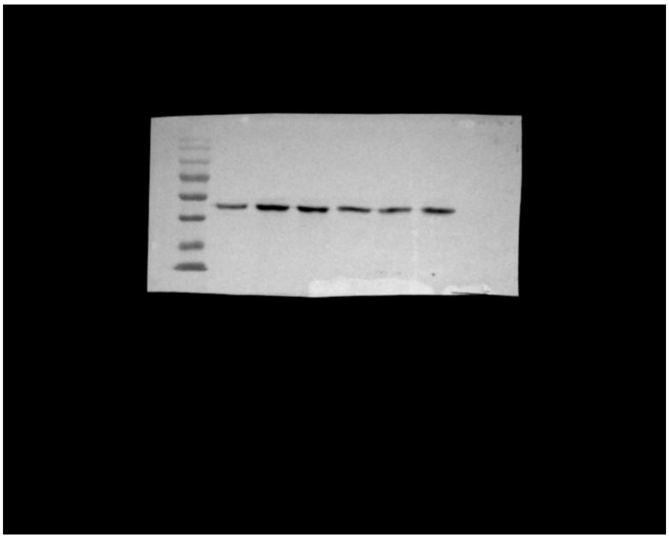

GAPDH

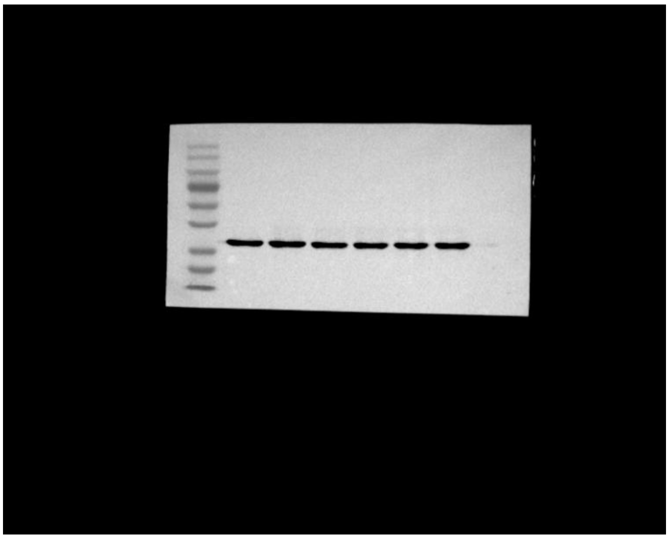

S-Figure 8A

SOGA1

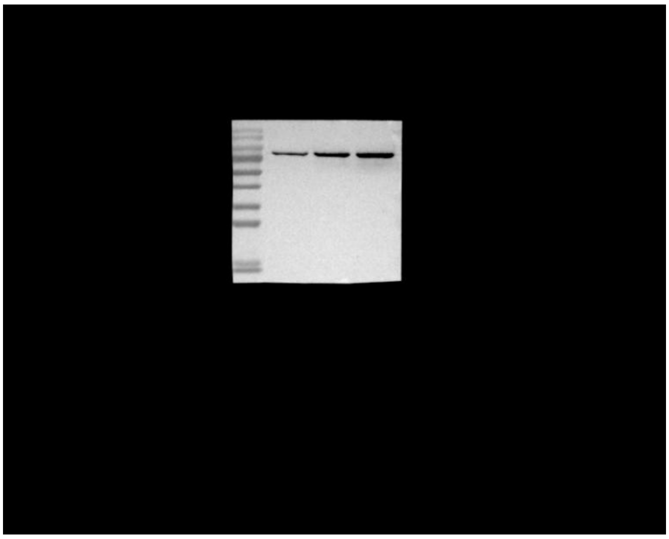

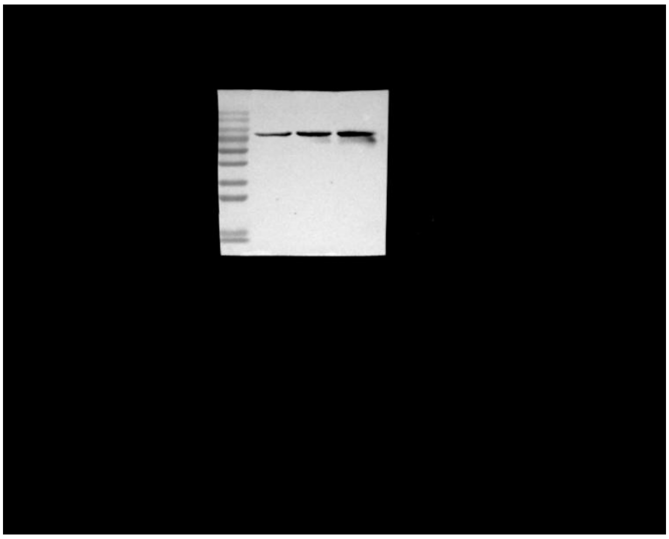

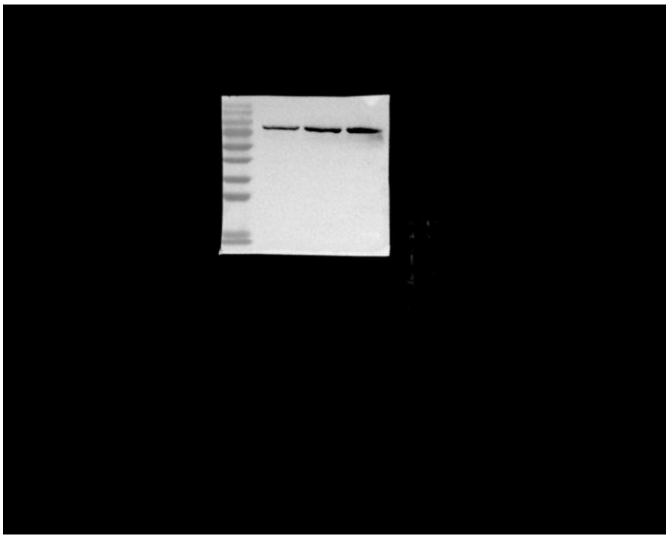

GAPDH

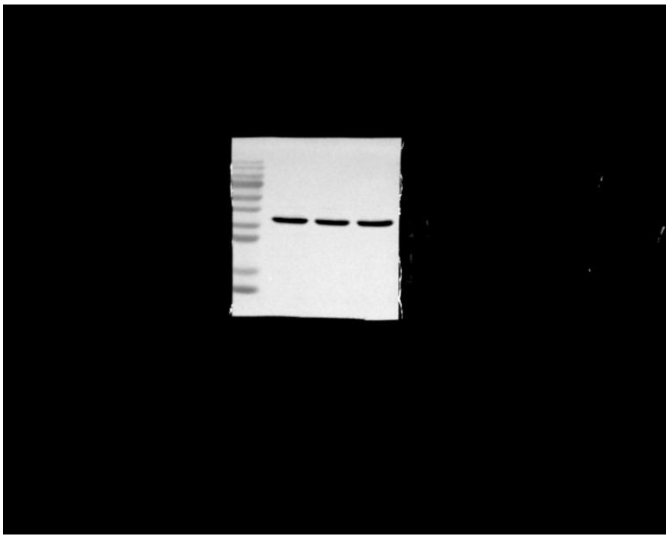

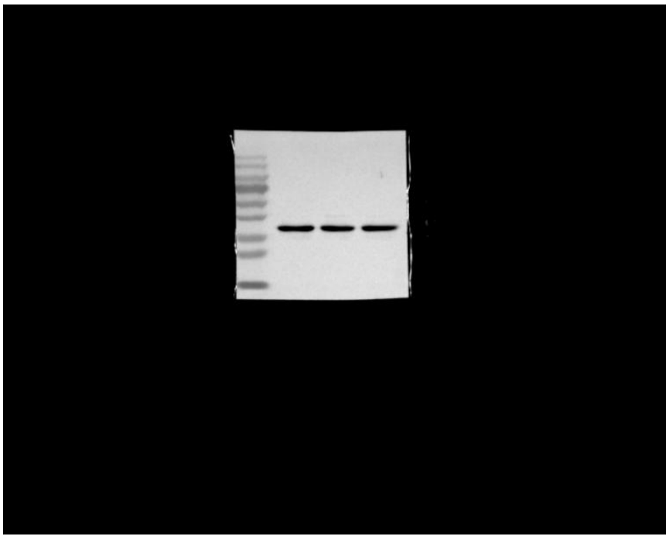

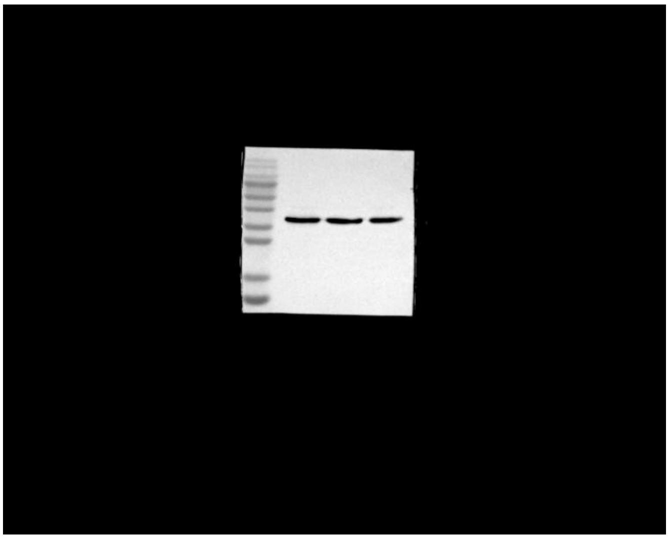

S-Figure 8B
SOGA1

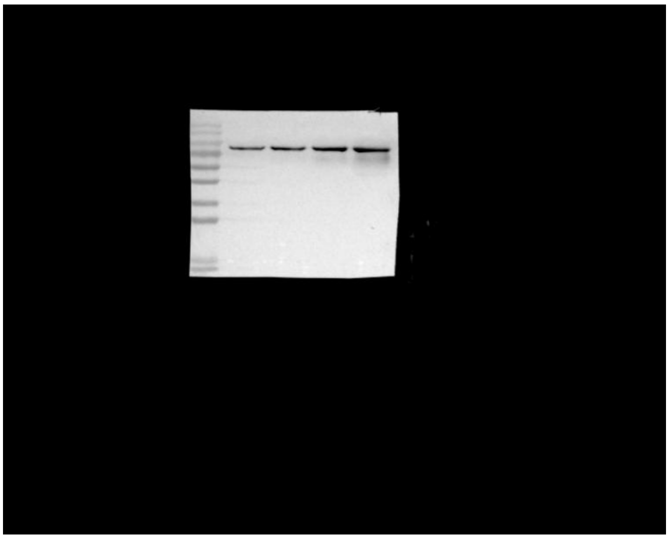

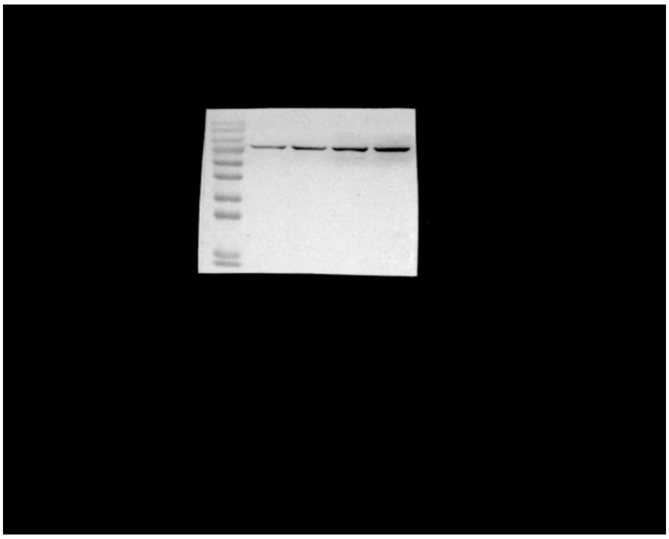

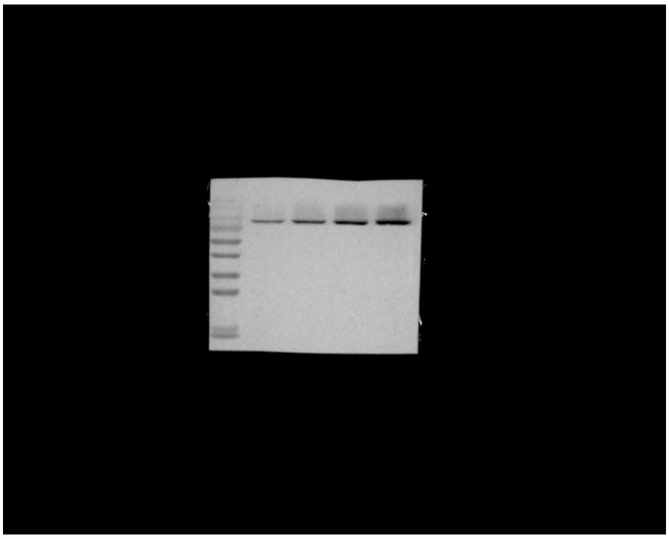

GAPDH

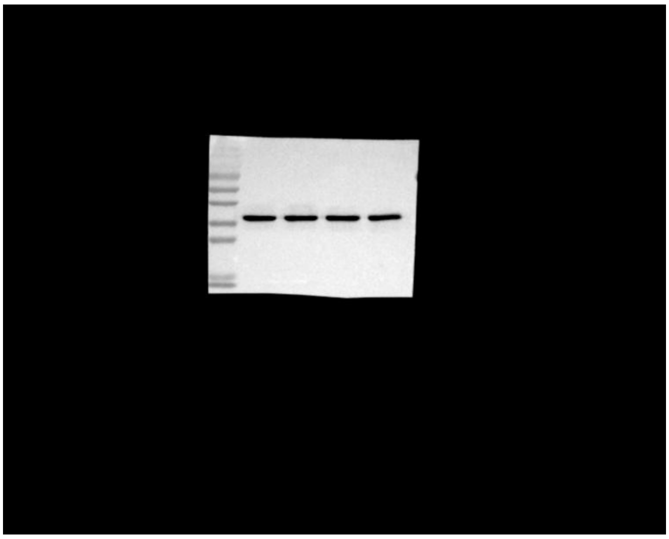

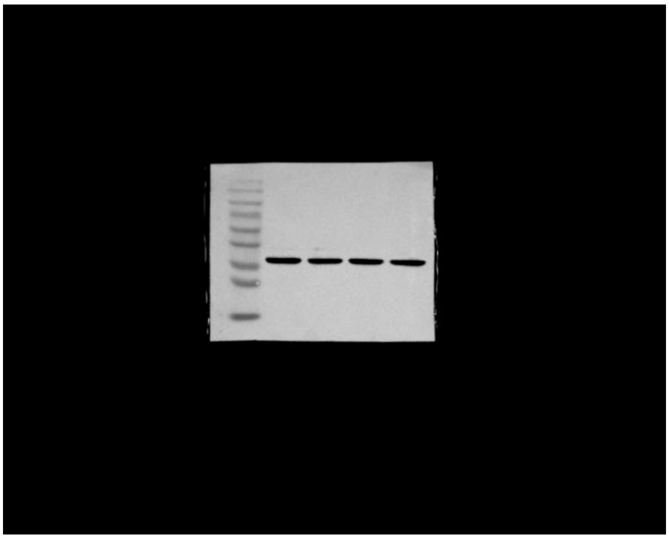

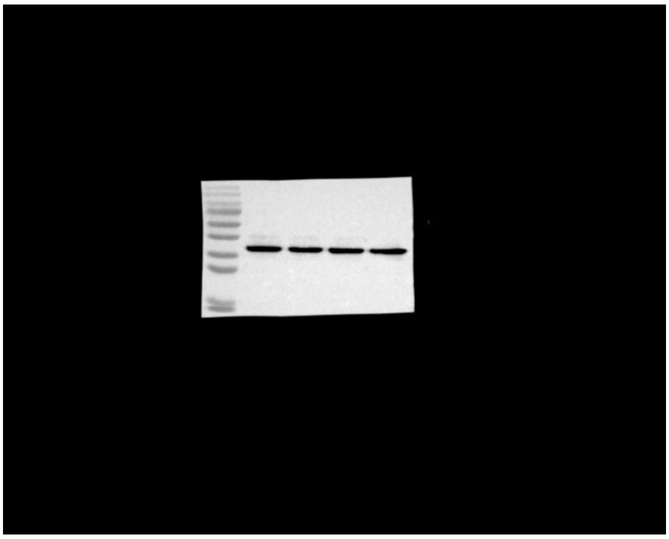

S-Figure 8C
SOGA1

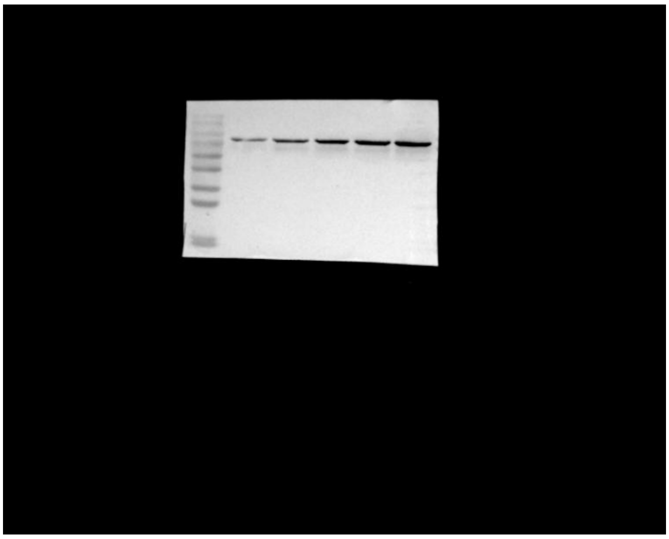

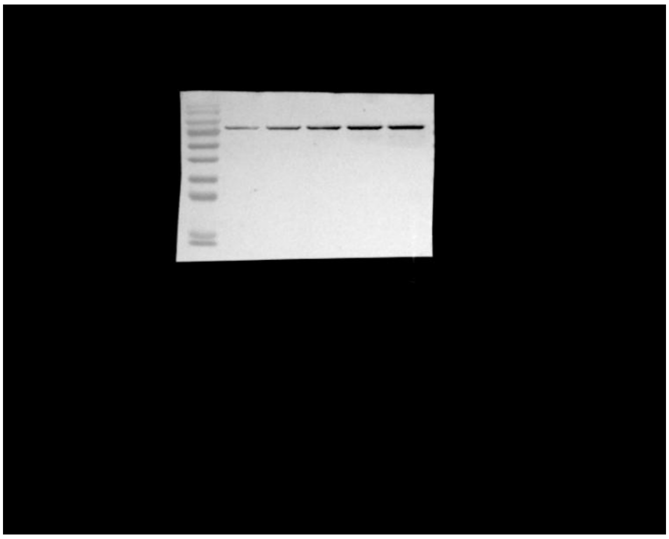

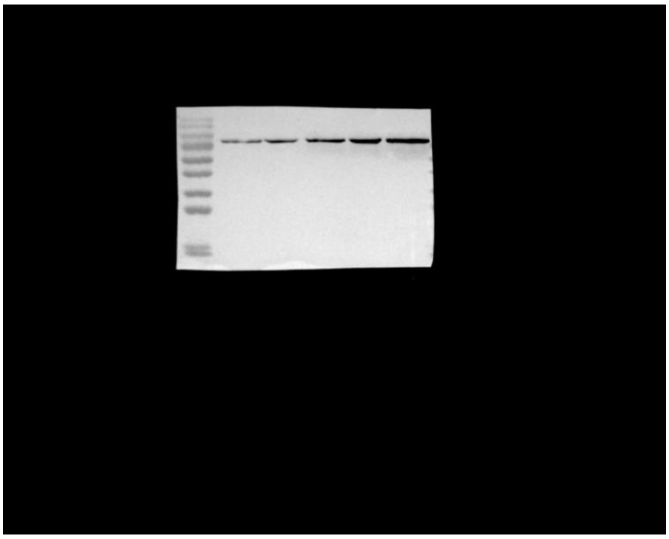

GAPDH

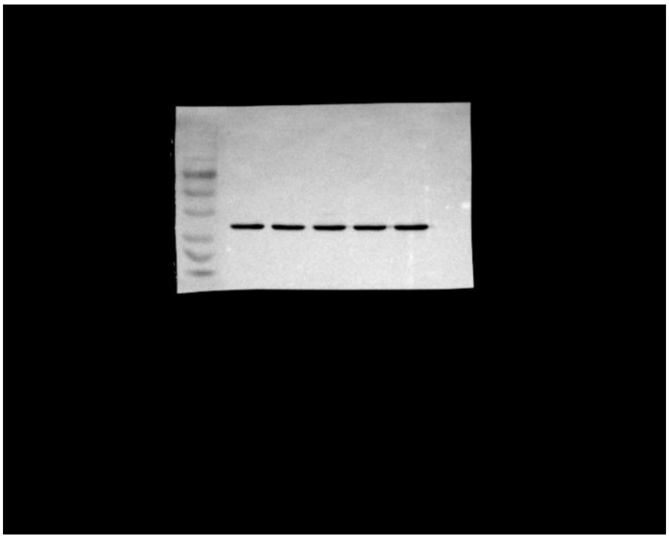

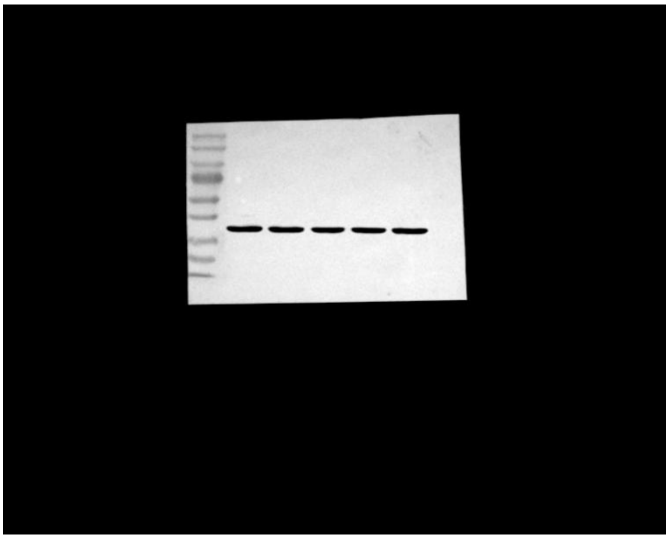

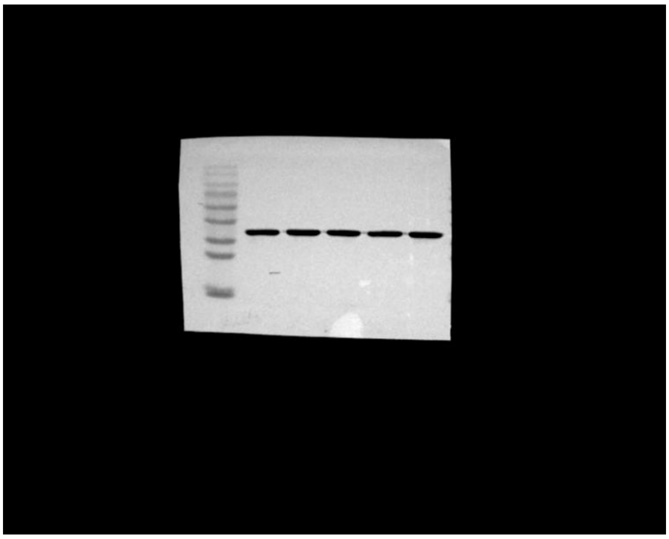

S-Figure 8D

TSG101

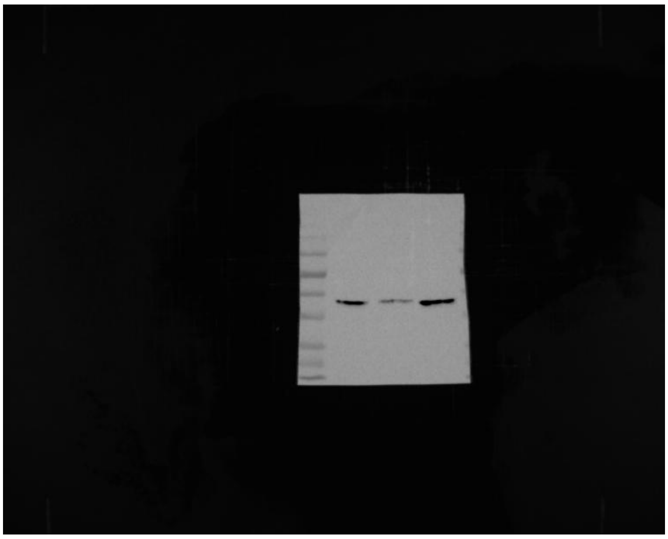

CD36

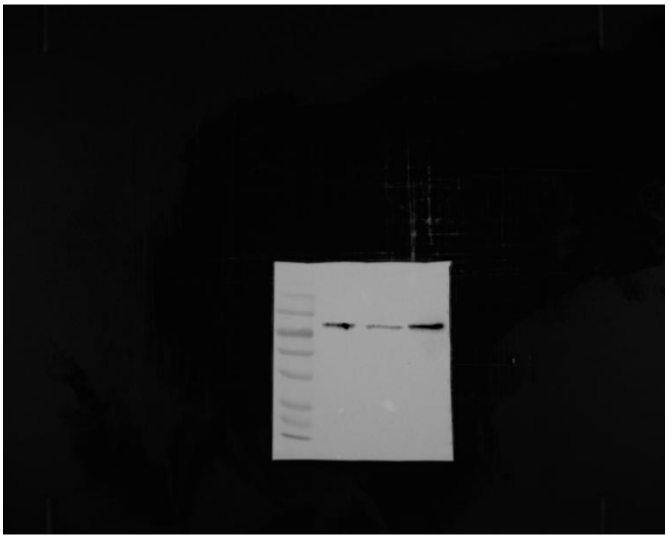

CD9

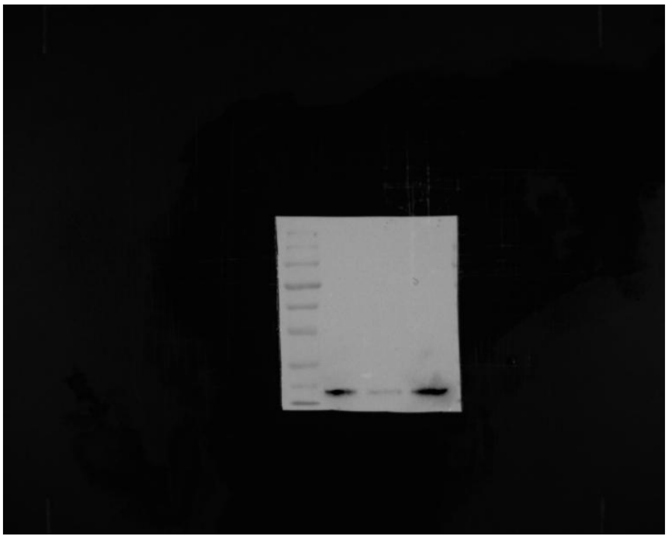

GAPDH

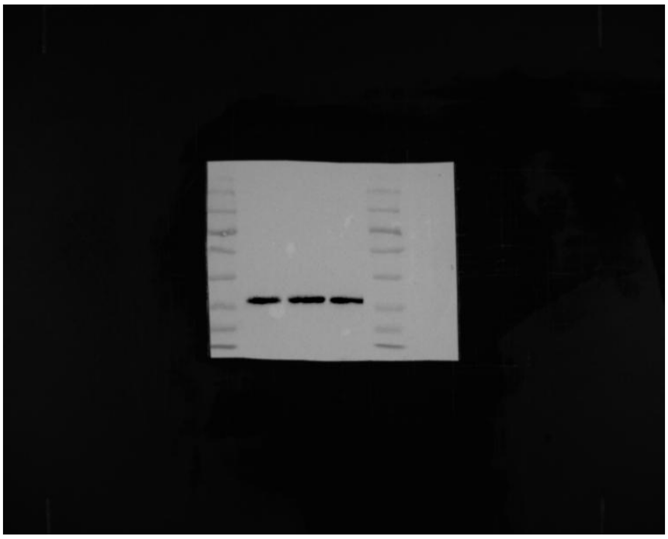

S-Figure 8E

SOGA

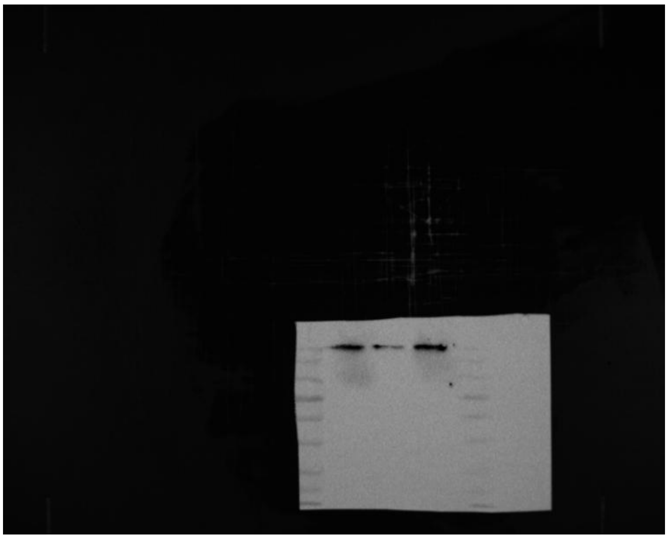

GAPDH

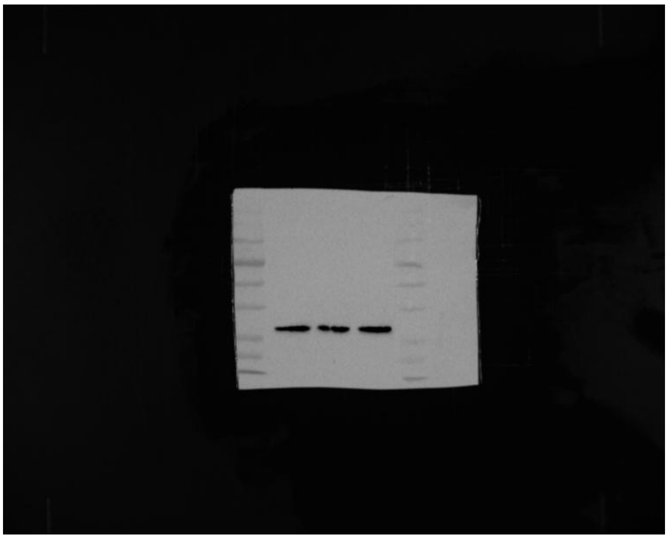

S-Figure 8F

SOGA1

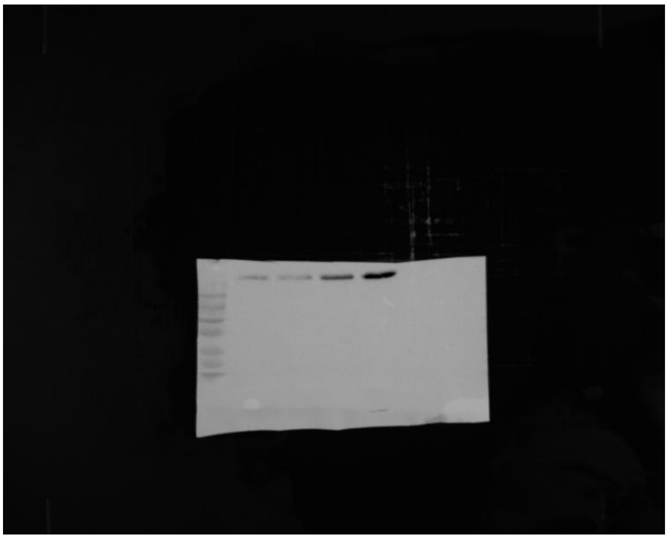

GAPDH

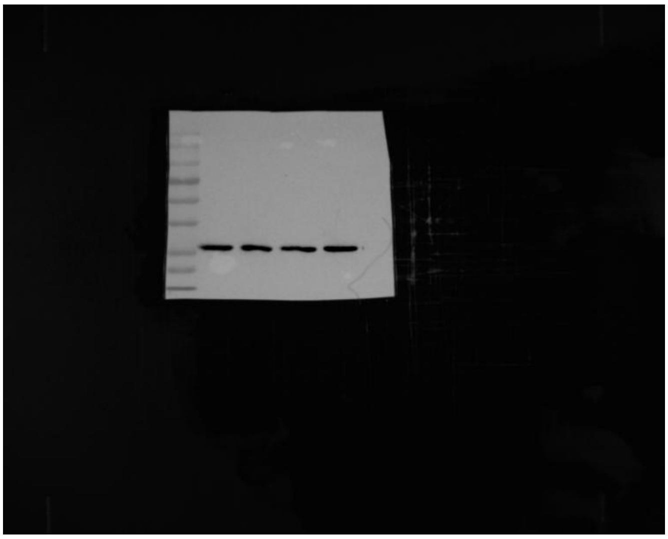

Supplement: Supplementary file 2 — Supplementary Material 2 [file 12896_2026_1162_MOESM2_ESM.pdf]
